# A SUMO-Dependent Protein Network Regulates Chromosome Congression during Oocyte Meiosis

**DOI:** 10.1016/j.molcel.2016.11.001

**Published:** 2017-01-05

**Authors:** Federico Pelisch, Triin Tammsalu, Bin Wang, Ellis G. Jaffray, Anton Gartner, Ronald T. Hay

**Affiliations:** 1 Centre for Gene Regulation and Expression, Sir James Black Centre, School of Life Sciences, University of Dundee, Dundee, DD1 5EH, UK

**Keywords:** SUMO, PIAS, GEI-17, meiosis, *C. elegans*, SUMO-interaction motif, kinesin, spindle, chromosomes, CRISPR

## Abstract

During *Caenorhabditis elegans* oocyte meiosis, a multi-protein ring complex (RC) localized between homologous chromosomes, promotes chromosome congression through the action of the chromokinesin KLP-19. While some RC components are known, the mechanism of RC assembly has remained obscure. We show that SUMO E3 ligase GEI-17/PIAS is required for KLP-19 recruitment to the RC, and proteomic analysis identified KLP-19 as a SUMO substrate in vivo. In vitro analysis revealed that KLP-19 is efficiently sumoylated in a GEI-17-dependent manner, while GEI-17 undergoes extensive auto-sumoylation. GEI-17 and another RC component, the kinase BUB-1, contain functional SUMO interaction motifs (SIMs), allowing them to recruit SUMO modified proteins, including KLP-19, into the RC. Thus, dynamic SUMO modification and the presence of SIMs in RC components generate a SUMO-SIM network that facilitates assembly of the RC. Our results highlight the importance of SUMO-SIM networks in regulating the assembly of dynamic protein complexes.

## Highlights

• SUMO conjugates accumulate in the ring complex within the midbivalent during meiosis
• Ring complex assembly requires active sumoylation
• The SUMO E3 ligase GEI-17/PIAS sumoylates the chromokinesin KLP-19 in vivo and in vitro
• Meiotic chromosome congression depends on sumoylation and non-covalent SUMO binding

## Graphical Abstract

**Figure:**
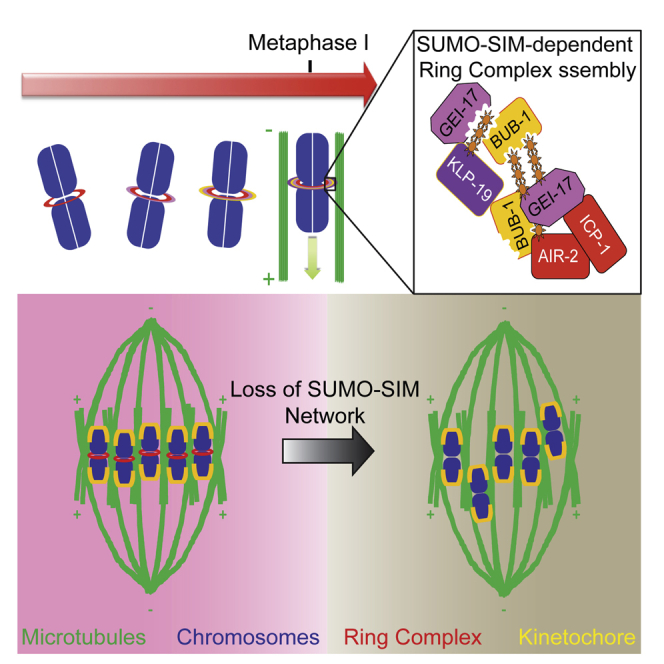

## Teaser

Pelisch et al. provide evidence that highly dynamic, coordinated, and spatially constrained sumoylation regulates chromosome congression during meiosis in *C. elegans* oocytes.

## Introduction

Meiosis is a specialized division in which a single round of DNA replication is followed by two consecutive segregation steps. Homologous chromosomes segregate in Meiosis I, while sister chromatids segregate in Meiosis II, giving rise to haploid gametes (Duro and Marston, 2015). In contrast to mitotic spindles, meiotic spindles in many animal species (including humans and nematodes) lack centrosomes (Dumont and Desai, 2012), and how these spindles are organized is poorly understood (Ohkura, 2015). As meiotic spindles vary across the animal kingdom, identification of common and unique mechanisms of spindle assembly and chromosome orientation, congression, and segregation will contribute to the fundamental understanding of these processes (Dumont and Desai, 2012, Severson et al., 2016). In *C. elegans* oocytes, chromosome movement along lateral microtubule bundles is facilitated by plus-end directed forces exerted by the kinesin KLP-19 (Powers et al., 2004, Wignall and Villeneuve, 2009), which requires the kinase BUB-1 for localization to the ring complex (RC). BUB-1, in turn, requires the chromosomal passenger complex (CPC) components AIR-2/Aurora B and ICP-1/INCENP for RC localization (Dumont et al., 2010, Wignall and Villeneuve, 2009). However, the mechanism of RC assembly has remained elusive.

The small ubiquitin-related modifier (SUMO) conjugation pathway is conserved in *C. elegans*, and it is composed of one SUMO ortholog, SMO-1 (hereafter, SUMO), an E1 activating enzyme, and the E2 conjugating enzyme (UBC-9). The specificity and dynamic nature of this modification is achieved by SUMO-specific E3 ligases and SUMO-specific isopeptidases (Flotho and Melchior, 2013, Gareau and Lima, 2010, Hay, 2007). In *C. elegans*, these include the Siz/PIAS-type SUMO E3 ligase GEI-17 (Kim and Michael, 2008, Pelisch et al., 2014) and the isopeptidases ULP-1, 2, 4, and 5 (Pelisch et al., 2014, Sapir et al., 2014, Tsur et al., 2015, Zhang et al., 2004). SUMO E3 ligases often target groups of functionally related proteins (Psakhye and Jentsch, 2012) and, once this modification has taken place, the presence of SIMs in PIAS type E3 ligases has the potential to prolong engagement on substrate(s) leading to a dramatic amplification of the signal. Thus, SUMO-SIM networks provide a means to rapidly and reversibly regulate protein interactions.

While sumoylation regulates chromosome synapsis during meiosis in budding yeast (Cahoon and Hawley, 2016), it is not known whether this is conserved in other organisms. We addressed this using the nematode *C. elegans,* as it provides an excellent model to study meiosis (Hillers et al., 2015). In nematodes, synaptonemal complex (SC) assembly is unperturbed in the absence of SUMO, while bivalent differentiation and SC disassembly are affected (Bhalla et al., 2008). Here, we show that highly dynamic, GEI-17-mediated SUMO conjugation during fertilization and non-covalent SUMO binding facilitates the assembly of a complex containing AIR-2/Aurora B, BUB-1, and the chromokinesin KLP-19. We identified GEI-17/PIAS as the key SUMO E3 ligase required for this complex to assemble and show that it is directly involved in SUMO modification of KLP-19. SIMs present in BUB-1 and GEI-17 allow them to bind SUMO-modified forms of KLP-19, providing a mechanism for SUMO-dependent RC assembly. These results highlight the requirement for spatially and temporally regulated SUMO modification during *C. elegans* oocyte meiosis and illustrate how post-translational modifications can regulate chromosome congression on acentrosomal spindles.

## Results

### SUMO in the *C. elegans* Germline

**Figure 1:**
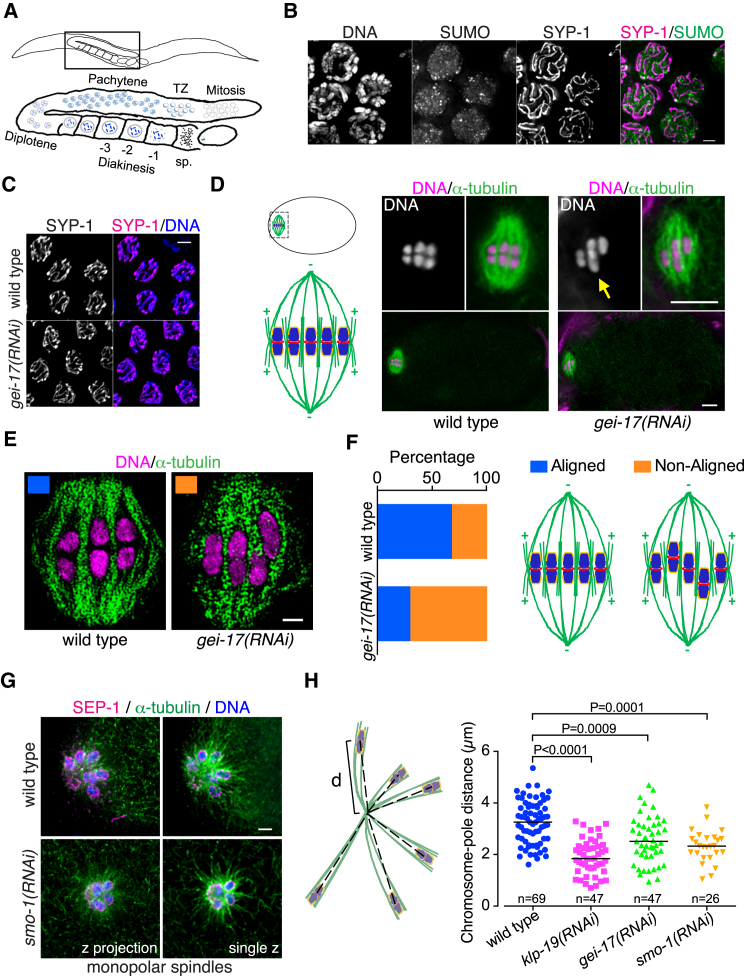
Sumoylation Is Required for Meiotic Chromosome Congression (A) Schematic of a worm highlighting the germline and the different stages of prophase. transition zone, TZ; spermatheca, sp. (B) Nuclei in the pachytene stage of meiosis showing SUMO localization in green and the synaptonemal complex component SYP-1 in magenta. The scale bar represents 2 μm. See also Figure S1B. (C) SYP-1 staining in pachytene nuclei from wild-type and *gei-17(RNAi)* worms. The scale bar represents 3 μm. See also Figure S1E. (D) The schematic shows a fertilized oocyte (top left) and highlights its anterior end and the meiotic spindle (bottom left). The metaphase I-arrested oocytes from *mat-1* wild-type or *gei-17(RNAi)* worms were stained for α-tubulin (green) and DNA (magenta) and widefield images are displayed. The yellow arrow points to a misaligned bivalent. The scale bar represents 5 μm. The bottom images display a view of the entire oocyte for each condition. (E) Same as in (F), but 3D-structured illumination (SIM) images were acquired using an OMX microscope. Note that the wild-type spindle in (E) is the same as the wild-type spindle in (D). The scale bar represents 1 μm. (F) Spindles were characterized as either “aligned” or “non-aligned” (at least one chromosome away from the metaphase plate) and results from 25 oocytes for each condition are shown as % of total. (G) Monopolar spindles arrested at metaphase were obtained from *klp-18(ok2519)* worms (KLP-18 is an essential protein to achieve bipolarity) in the presence of *emb-30(RNAi)*, either in the absence or presence of *smo-1(RNAi)*. α-tubulin is shown in green, DNA in blue, and SEP-1 (as a marker for metaphase) in magenta. The scale bar represents 2 μm. (H) The 3D distance (d) between the spindle pole and the middle of each bivalent was measured (number of measurements are shown for each condition as “n”), and the results are represented in a dot plot with the median shown as a black horizontal line. The results were analyzed with the Kruskal-Wallis test, followed by Dunn’s post-test.

While sumoylation plays a role in meiotic chromosome pairing during prophase in yeast, this is not conserved in nematodes, where synapsis occurs normally in the absence of SUMO (Bhalla et al., 2008). While SUMO knockout worms have severe germline defects (Broday et al., 2004), we found that in SUMO E3 ligase *gei-17* −*/*− worms, some oocytes mature in spite of the defective germline and accumulate the CPC protein ICP-1 in the midbivalent and the cohesin REC-8 between homologous chromosomes and sister chromatids (Figure S1A). As opposed to the SC components SYP-1 and HTP-3, SUMO displays a diffuse localization in pachytene nuclei (Figures 1A, 1B, and S1B). As expected (Bhalla et al., 2008), RNAi-mediated depletion of GEI-17 affected neither chromosome synapsis, as evidenced by SYP-1 staining (Figure 1C), nor crossover designation (Figures S1C and S1D). The diffuse SUMO signal in pachytene nuclei remained after GEI-17 depletion (Figure S1E), likely corresponding to unconjugated SUMO and/or SUMO conjugated by the action of other E3 ligase(s). Enzymes of the SUMO pathway do not co-localize with DNA during pachytene: UBC-9 co-localizes with the nuclear envelope, while GEI-17 accumulates on the inner side of the nuclear envelope (Figure S1F). The SUMO protease ULP-1 also localizes in the nuclear envelope (data not shown). These results show that meiosis can proceed through pachytene when GEI-17-mediated SUMO conjugation is compromised.

### Lack of Sumoylation Affects Chromosome Congression

Live imaging experiments using mCherry-H2B showed that in the absence of GEI-17, chromosomes often failed to align during metaphase I (Figure S2A). To robustly characterize this phenotype, we used an anaphase promoting complex temperature-sensitive allele: *emb-27* (Golden et al., 2000), focusing on the oocyte closest to the spermatheca to avoid indirect effects derived from prolonged arrest. In the absence of GEI-17, chromosome alignment was compromised, as evidenced by the presence of one or two chromosomes closer to one of the spindle poles (Figures 1D, yellow arrows, 1E, and 1F). This phenotype is reminiscent of *klp-19(RNAi)* (Wignall and Villeneuve, 2009), consistent with a plus-end-directed force defect. As monopolar spindles allow the contributions of plus- and minus-end-directed forces to be evaluated (Muscat et al., 2015, Wignall and Villeneuve, 2009), we generated metaphase I-arrested monopolar spindles. Metaphase I chromosomes localize close to the plus-end of microtubules (Figure 1G) (Wignall and Villeneuve, 2009), but knock down of SUMO, UBC-9, or GEI-17 reduced the chromosomes-to-pole distance (Figures 1G and 1H). Separase (SEP-1) staining was used to confim that oocytes were in metaphase I (Bembenek et al., 2007, Muscat et al., 2015). Thus chromosome congression fails in the absence of sumoylation due to a defect in plus-end directed forces.

### SUMO Localizes to a Ring-Shaped Structure during Meiosis

**Figure 2:**
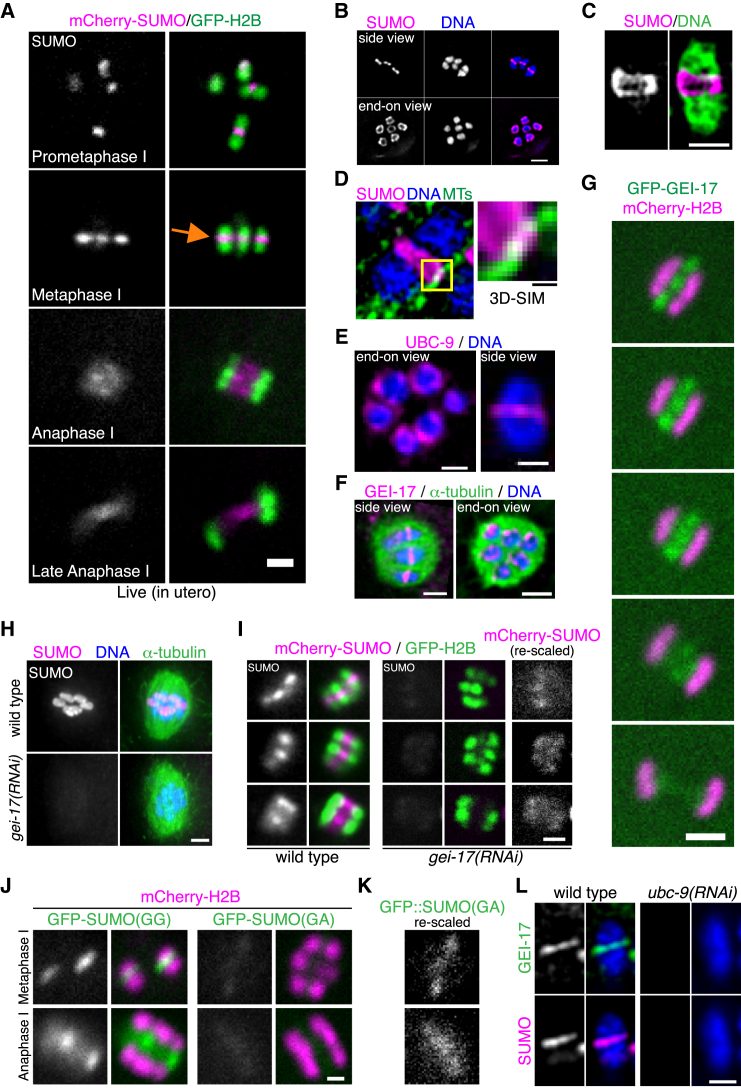
SUMO Conjugation during Meiosis (A) Meiosis I in worms expressing mCherry-SUMO/GFP-H2B was followed by in utero time lapse. The still images of the different stages are shown with SUMO colored in magenta and H2B in green. The scale bar represents 2 μm. See also Figure S2B. (B) The upper row displays a side view of the bivalents in metaphase I with SUMO shown in magenta and DNA in blue in the merged image. The lower row displays another set of bivalents in metaphase I from an end-on view. The scale bar represents 2 μm. (C) 3D-SIM image of a single bivalent stained with SUMO (magenta) and DNA (green) is shown. The scale bar represents 1 μm. See also Figure S2C. (D) 3D-SIM was used to analyzed a metaphase I meiotic spindle showing SUMO in magenta, α-tubulin in green, and DNA in blue. The area delimited by the yellow square is enlarged on the right. The scale bar represents 0.2 μm. See also Figure S2D. (E) The SUMO E2 conjugating enzyme UBC-9 also localizes to the ring-shaped midbivalent structure. An end-on view of the whole set of chromosomes is displayed on the left and a side view on a single bivalent is shown on the right. The scale bar represents 1 μm. (F) GEI-17 is shown in magenta, along with microtubules in green, and DNA in blue. The left image shows a side view of the spindle, while the right image displays an end-on view. The scale bar represents 2 μm. (G) The localization of GEI-17 was followed through anaphase I by tagging the endogenous protein with GFP (see Experimental Procedures). Meiosis was followed as explained in (A). The scale bar represents 2 μm. (H) Wild-type and *gei-17(RNAi)* embryos were fixed and stained with SUMO (magenta), DNA (blue), and α-tubulin (green). The scale bar represents 2 μm. (I) Worms expressing mCherry-SUMO and GFP-H2B were fed control (wild-type) or *gei-17(RNAi)* and oocyte meiosis was recorded in utero. The scale bar represents 2 μm. The mCherry signal from *gei-17(RNAi)* oocytes was re-scaled and shown on the right. (J) The conjugation dependency on SUMO localization was analyzed by comparing the localization of GFP-tagged SUMO(GG) and the non-conjugatable form SUMO(GA). The stills from live imaging experiments are shown for metaphase and anaphase from meiosis I. The scale bar represents 1 μm. (K) The GFP fluorescence from GFP-SUMO(GA) was re-scaled to enhance detection of the small amount of SUMO remaining. (L) GEI-17 recruitment to the midbivalent is UBC-9-dependent. The worms were fed control or *ubc-9(RNAi)* and stained for GEI-17 (green), SUMO (magenta), and DNA (blue). The channel corresponding to SUMO fluorescence was re-scaled as in (D). The scale bar represents 1 μm.

mCherry-SUMO strongly concentrates at the midbivalent in metaphase I and within sister chromatids in metaphase II (Figures 2A and S2B). During anaphase I and II, SUMO is detected between the segregating chromosomes and chromatids, respectively (Figures 2A and S2B) and becomes diffuse within the spindle in late anaphase (Figure 2A). Endogenous SUMO also concentrates in the midbivalent during metaphase I in a ring-shaped pattern (Figures 2B, 2C, and S2C) and partially co-localizes with microtubule bundles (Figures 2D and S2D). The same localization pattern was observed for UBC-9 (Figure 2E) and GEI-17 (Figure 2F). Endogenous, GFP-tagged GEI-17 localization is also dynamic being present in the midbivalent before localizing to the spindle midzone and then fading away by late anaphase (Figure 2G). This suggests that active sumoylation takes place within the RC and that one or more microtubule-associated proteins are SUMO substrates.

### SUMO Conjugation within the Ring Complex

Knock down of GEI-17 dramatically reduces SUMO localization at the midbivalent (Figures 2H and 2I), although residual SUMO remains at the midbivalent and spindle (Figure 2I; mCherry channel “re-scaled”). To test if conjugation is required for the formation of the midbivalent SUMO ring, we expressed a GFP-SUMO mutant with the C-terminal Gly-Gly sequence mutated to Gly-Ala [“GFP-SUMO(GA)”] to block conjugation to substrates (Pelisch et al., 2014). GFP-SUMO(GA) localization to the midbivalent is dramatically reduced (Figure 2J), and residual localization can only be observed by rescaling the GFP fluorescence (Figure 2K). Thus, conjugation is the primary determinant for SUMO localization in the RC during meiosis, although a role for non-covalent SUMO interactions is also suggested. Supporting this argument, knock down of the SUMO E2 enzyme, UBC-9, not only inhibits SUMO concentration in the midbivalent, but also abolishes recruitment of the E3 ligase GEI-17 (Figure 2L). Thus, SUMO conjugation is required for E3 ligase recruitment and for SUMO to concentrate on the ring.

### The Kinesin KLP-19 Is a Substrate for SUMO Modification and Exhibits SUMO-Dependent Localization

**Figure 3:**
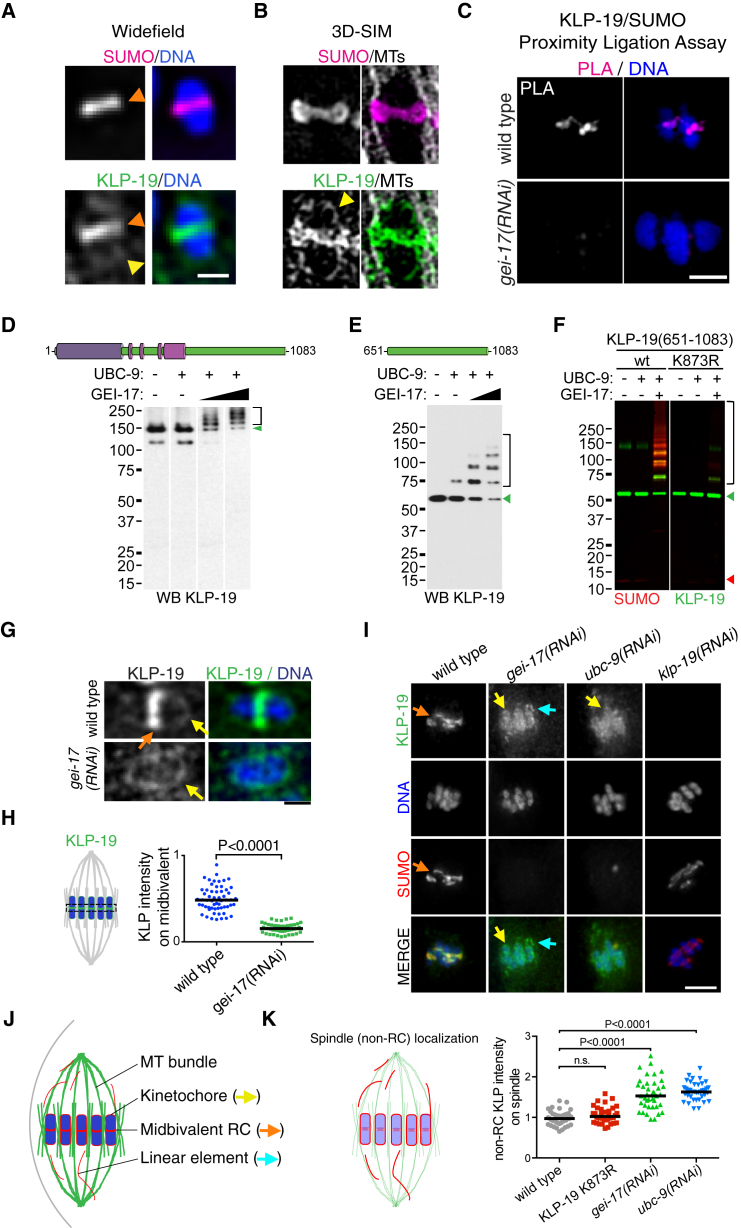
KLP-19 Is a SUMO Substrate and Its Ring Localization Depends on SUMO Conjugation (A) Widefield images of a single bivalent stained for SUMO (top) and KLP-19 (bottom). The yellow arrow points to the kinetochore, while the orange arrow marks the midbivalent. The scale bar represents 1 μm. (B) 3D-SIM images of a single bivalent stained for SUMO (magenta) or KLP-19 (green) and α-tubulin (gray). (C) Proximity ligation assay of the KLP-19-SUMO interaction in control and *gei-17(RNAi)* worms. The bivalents during the first meiotic division are shown. The scale bar represents 2 μm. (D) The cartoon depicts the domain architecture of KLP-19. The motor domain is shown in purple and the putative coiled-coil domains in pink. Full-length, recombinant KLP-19 was incubated in the presence of SUMO (20 μM), E1 enzyme (100 nM), UBC-9 (140 nM), and GEI-17 (12.5 and 25 nM). An aliquot of the reaction was run on an SDS-PAGE, and western blot was performed with an anti-KLP-19 antibody. The green arrowhead points to unmodified KLP-19, and the square bracket denotes SUMO-modified KLP-19. See also Figures S4A, S4B, S4D, and S4E. (E) In vitro SUMO conjugation reactions were performed with KLP-19(651–1,083) as substrate. The reactions were incubated in the presence of SUMO (20 μM), E1 enzyme (100 ng), UBC-9 (140 nM), and increasing amounts of GEI-17 (12.5 and 25 mM). The reactions were developed as in (A). The green arrowhead points to unmodified KLP-19, and the square bracket denotes SUMO-modified KLP-19. See also Figures S4A, S4C, S4D, and S4F. (F) Wild-type and K873R KLP-19(651–1,083) were subject to GEI-17-dependent in vitro SUMO conjugation, using the same conditions as above and 12.5 mM GEI-17. The reactions were then run on a gel, transferred to a membrane, and developed by two-color near-infrared western blotting (LICOR). KLP-19 is shown in green and SUMO in red. The red arrowhead indicates free SUMO; the green arrowhead points to unmodified KLP-19; and the square bracket denotes SUMO-modified KLP-19. See also Figures S3A–S3I, S4G, and S4H. (G) KLP-19 localization in the midbivalent is dependent on GEI-17. Metaphase I-arrested oocytes were stained for KLP-19, SUMO, and DNA as indicated. The yellow arrow points to the kinetochore, while the orange arrow marks the midbivalent. (H) Quantitation of the KLP-19 intensity in the midbivalent was analyzed using the Mann-Whitney test. The black lines indicate the median. (I) KLP-19 localization was analyzed after GEI-17 or UBC-9 depletion. An orange arrow signals KLP-19 on the midbivalent. The yellow arrow indicates kinetochore localization. The blue arrow indicates the linear elements (see main text for details). The scale bar represents 4 μm. See also Figure S5D. (J) Schematic depicting the procedure for quantifying KLP-19 residing outside the RC (“non-RC”). The microtubule bundles, kinetochores, midbivalents, and linear elements are shown next to the colored arrows used throughout the figure to highlight them. The whole spindle area was selected using α-tubulin as a guide, and the midbivalents were selected from the DNA channel. After substracting the background-corrected midbivalent signal to the background-corrected spindle signal, we obtained the non-RC KLP-19 intensity. (K) KLP-19 re-localizes to kinetochores and linear elements after UBC-9 or GEI-17 depletion. The data for non-RC KLP-19 intensity are shown as a dot plot, and the samples were compared using a Kruskal-Wallis test, followed by Dunn’s post-test. The black lines denote the medians.

To search for SUMO substrates during meiosis, we adapted to *C. elegans* a proteomics approach successfully employed in human cells (Tammsalu et al., 2014, Tammsalu et al., 2015). To identify sumoylation sites in vivo, we generated worms expressing His_6_-tagged SUMO with a Leu to Lys substitution preceding the C-terminal diGly motif (L88K) in the germline and early embryos (Figure S3A). After Ni-NTA purification and Lys-C digestion, the mutant SUMO leaves a GG remnant on substrate lysines that facilitates peptide enrichment with an anti-K-ε-GG antibody (Figure S3B) (Tammsalu et al., 2014, Tammsalu et al., 2015). Conjugation of SUMO(L88K) to substrates in vitro is indistinguishable from wild-type SUMO (Figures S3C and S3D) and is also conjugated in vivo (Figure S3E). Among the in vivo substrates, we obtained evidence for modification of KLP-19 within its C-terminal region (Figure S3F). Mass spectrometry analysis of in vitro sumoylated KLP-19 showed that Lys 873, contained in the peptide identified in vivo, is a SUMO modification site in KLP-19 (Figures S3G–S3I), although other lysines were identified as SUMO acceptors (Figure S7). SUMO and KLP-19 both localize within the midbivalent (Figures 3A and 3B, orange arrowhead), while a small fraction of KLP-19 also localizes to kinetochores (Figures 3A and 3B, yellow arrowhead). Juxtaposition of SUMO and KLP-19 in vivo was confirmed by proximity ligation assays (PLA) and is dependent on the SUMO E3 ligase GEI-17 (Figure 3C). To confirm that KLP-19 is a SUMO substrate, we performed in vitro sumoylation assays using bacterially expressed, purified proteins (Figures S4A–S4D). Full-length, untagged KLP-19 is efficiently modified by SUMO in a GEI-17-dependent manner (Figures 3D and S4E). As the identified SUMO modification site in vivo localizes within the C-terminal region of KLP-19, we performed in vitro sumoylation reactions using KLP-19(651–1,083), which excludes the motor and coiled-coil domains. KLP-19(651–1,083) is efficiently sumoylated in a GEI-17-dependent manner (Figures 3E and S4F), and we confirmed that the slower migrating species corresponded to SUMO-modified KLP-19 using two-color western blot (Figures 3F and S4G). Importantly, mutation of lysine 873 to arginine within KLP-19 (K873R) drastically reduced SUMO modification of KLP-19 (Figures 3F, S4G, and S4H). Co-depletion of GEI-17 and KLP-19 followed by quantification of the congression defect indicated that the two proteins act on the same genetic pathway (Figure S5A). Accumulation of KLP-19 in the RC is drastically reduced in the absence of GEI-17 (Figures 3G, orange arrow, and 3H), while the faint kinetochore signal is unaffected (Figure 3G, yellow arrows). GEI-17 or UBC-9 depletion revealed that in the absence of sumoylation, KLP-19 localized partially in kinetochores (Figure 3I, yellow arrows) and in thread-like structures that did not co-localize with microtubule bundles (Figure 3I, cyan arrows). Interestingly, kinetochore proteins have been shown to concentrate not only in the classical cup-shaped structures surrounding the bivalents, but also in the so-called “linear elements” within the spindle and the cell cortex (Figure 3J) (Dumont et al., 2010, Monen et al., 2005). KLP-19 is not only present in the linear elements of the spindle, but also at the cell cortex (Figure S5D, cyan arrows). While mutation of Lys 873 to Arg in KLP-19 did not significantly affect its localization, general perturbation of the SUMO conjugation pathway lead to significant re-localization of KLP-19 away from the RC toward kinetochores and linear elements. Consistent with KLP-19 being a major SUMO substrate, *klp-19(RNAi)* leads to reduced ring localized SUMO (Figures S5B and S5C). Thus, KLP-19 is a SUMO substrate and sumoylation controls KLP-19 recruitment to the RC in vivo.

### Sumoylation Is Essential for the Assembly of the Ring Complex

**Figure 4:**
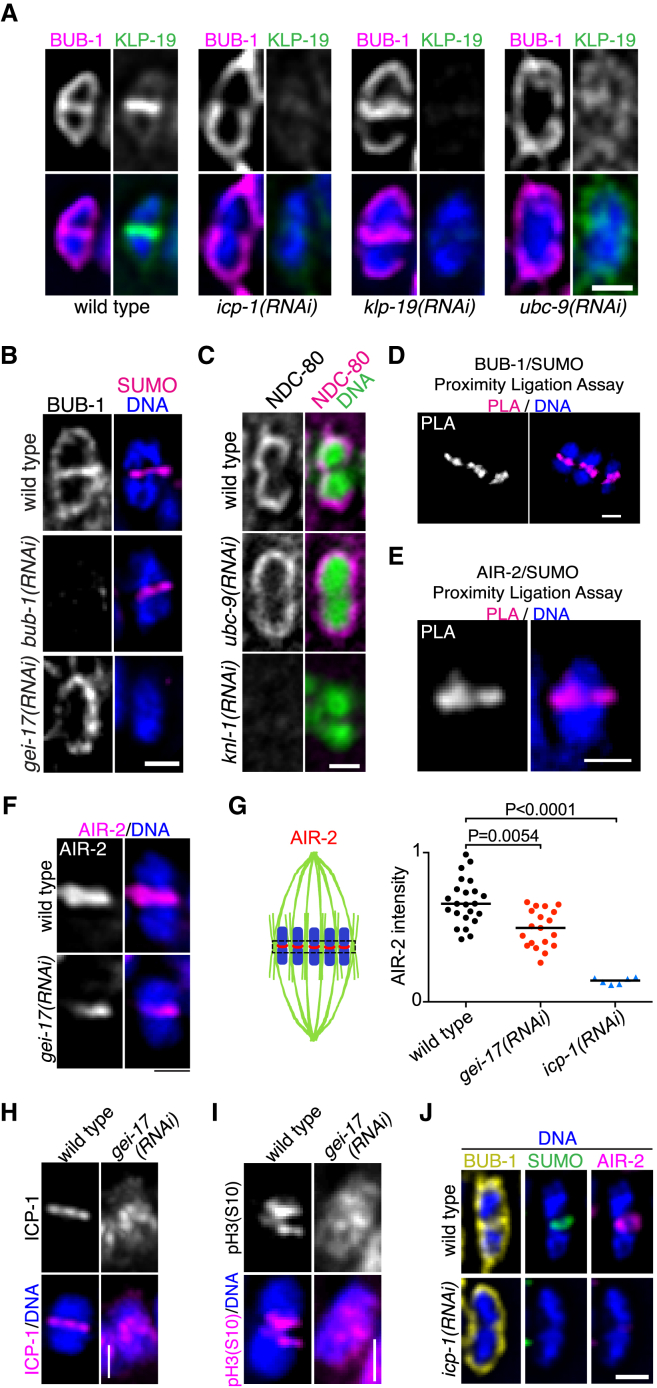
Ring Complex Assembly Depends on Sumoylation (A) BUB-1 and KLP-19 localization were analyzed in control (wild-type), *ubc-9(RNAi)*, *klp-19(RNAi)*, and *icp-1(RNAi)* in *emb-27* oocytes arrested at metaphase I. The scale bar represents 1 μm. See also Figures S6A and S6B. (B) The localization of BUB-1 and SUMO was analyzed as in (A) in control (wild-type), *bub-1(RNAi)*, and *gei-17(RNAi)* in *emb-27* oocytes arrested at metaphase I. The scale bar represents 1 μm. (C) Localization of the kinetochore component NDC-80 was analyzed in the presence of control RNAi (wild-type), *ubc-9(RNAi)*, or *knl-1(RNAi)* a known regulator of kinetochore assembly. The scale bar represents 1 μm. See also Figure S6C. (D) Proximity ligation assays were performed using BUB-1 and SUMO specific antibodies, and the PLA signal is shown in magenta and DNA in blue. The scale bar represents 1 μm. (E) Proximity ligation assays were performed using AIR-2 and SUMO specific antibodies, and the PLA signal is shown in magenta and DNA in blue. The scale bar represents 1 μm. (F) AIR-2 localization was in metaphase I-arrested oocytes from *emb-27* worms in the presence of control (wild-type) or *gei-17(RNAi)*. (G) AIR-2 fluorescence intensity in the midbivalent was quantified in wild-type, *gei-17(RNAi)*, and *icp-1(RNAi)* oocytes. The AIR-2 intensity is shown in the dot plot graph, and the samples were compared using a Kruskal-Wallis test, followed by Dunn’s post-test. The black lines denote the medians. (H) The absence of GEI-17 affects ICP-1/INCENP localization. ICP-1 localization was analyzed in wild-type or *gei-17(RNAi)* oocytes as above. The scale bar represents 1 μm. (I) The absence of GEI-17 affects phospho-Ser10-H3 localization. H3 phosphorylated on serine 10, known to be a CPC substrate, was analyzed in wild-type or *gei-17(RNAi)* oocytes as above. The scale bar represents 1 μm. (J) The CPC is required for ring assembly. Single bivalents co-stained for BUB-1 (yellow), SUMO (green), AIR-2 (magenta), and DNA (blue), either from wild-type or *icp-1(RNAi)* oocytes are shown. The scale bar represents 1 μm.

We then tested whether two other central RC components, BUB-1 and AIR-2, relied on sumoylation as a recruitment signal. As reported (Dumont et al., 2010, Wignall and Villeneuve, 2009), midbivalent localization of BUB-1 is dependent on ICP-1, but not on KLP-19 (Figure 4A). Depletion of *ubc-9* leads to a complete loss of BUB-1 at the midbivalent without affecting its kinetochore localization (Figures 4A, S6A, and S6B). In the absence of BUB-1, SUMO can still be detected in the midbivalent (Figure 4B). Sumoylation does not regulate kinetochore assembly as assessed by NDC-80 localization (Figures 4C and S6C). PLA assays show that SUMO is in close proximity to both BUB-1 (Figure 4D) and AIR-2 (Figure 4E) within the RC. In the absence of sumoylation, AIR-2 still localized between homologous chromosomes, although to a lesser extent (Figures 4F and 4G). Another CPC component, ICP-1/INCENP and the CPC substrate phospho-H3(S10), re-localize from the midbivalent to chromosomes in the absence of GEI-17 (Figures 4H and 4I). The midbivalent is also strongly stained with the anti-mitotic phospho-proteins MPM-2 antibody (Kitagawa and Rose, 1999), where it specifically recognizes the RC (Muscat et al., 2015). Depletion of GEI-17 leads to a dispersion of the MPM-2 signal, further showing that abolishing GEI-17-mediated sumoylation leads to RC disruption (Figure S6D). Additionally, completely disassembling the CPC by means of *icp-1(RNAi)* leads to the loss of the SUMO signal in the midbivalent (Figure 4J). Thus, the CPC and sumoylation are required for the RC to assemble, and we propose that this SUMO-dependent CPC assembly provides the basic platform for other components to associate with the RC. Initial or “seed” SUMO modification is thus CPC-dependent and likely to occur within the CPC itself, possibly with AIR-2 as a substrate.

### Acute, Germline-Specific Loss of GEI-17 Affects KLP-19 Recruitment to the Ring

**Figure 5:**
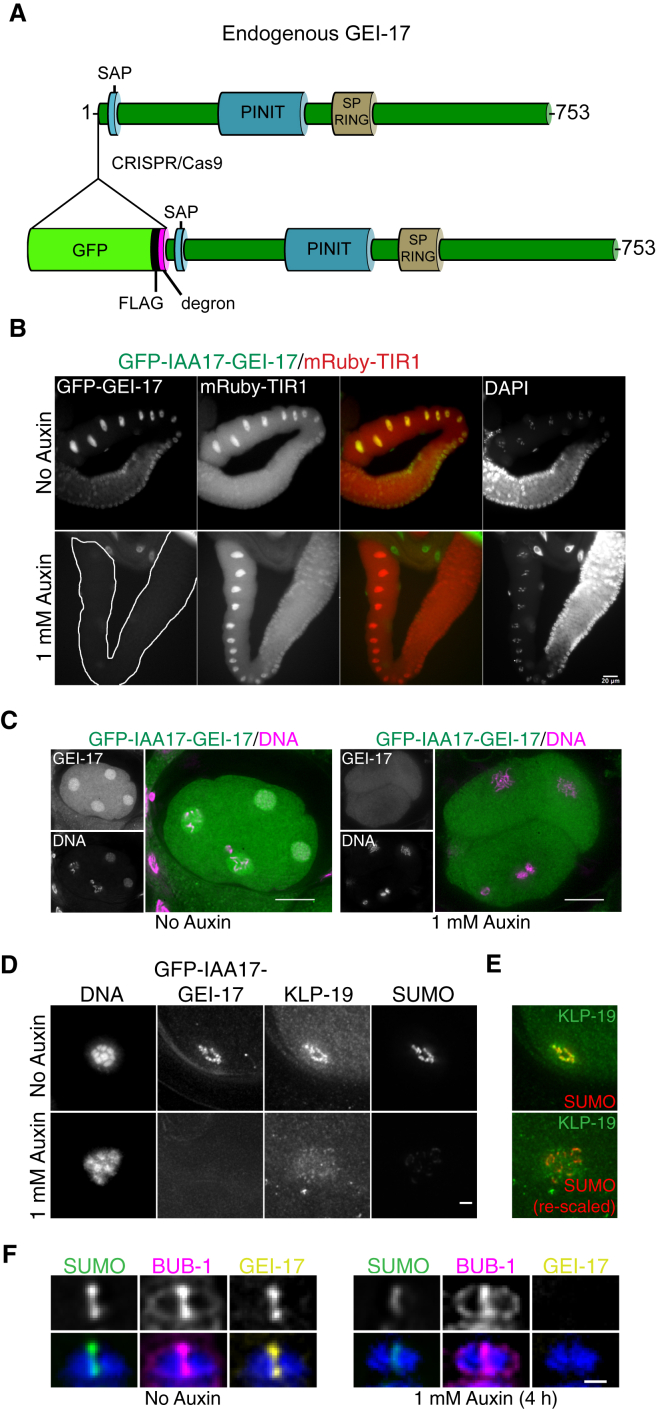
Tissue-Specific, Auxin-Induced Degradation of GEI-17 (A) Schematic of the generation of GFP-FLAG-degron-tagged endogenous GEI-17 by CRISPR/Cas9-mediated genome editing. The SAP and PINIT domains are highlighted, as well as the SP-RING. The numbering corresponds to GEI-17 isoform f (Uniprot Q94361-2). (B) Gonads from untreated or auxin-treated (1.5 hr) worms were dissected, fixed, and imaged for GFP (GEI-17) and mRuby (TIR1) fluorescence. The scale bar represents 20 μm. (C) Embryos from untreated or auxin-treated (1.5 hr) worms were fixed and imaged for GFP-GEI-17 (green) and DNA (magenta). The scale bar represents 10 μm. (D) Metaphase I oocytes from untreated or auxin-treated worms were fixed and imaged for GFP-GEI-17, KLP-19, and SUMO, along with DNA. Treatment with auxin for 4 hr is enough to reduce SUMO levels and also leads to a diffuse KLP-19 localization. (E) Re-scaling of the SUMO fluorescence shows that while some SUMO is still present, there is no specific co-localization with KLP-19. The scale bar represents 2 μm. (F) Metaphase I oocytes from untreated or auxin-treated worms were fixed and imaged for GFP-GEI-17 (yellow), BUB-1 (magenta), and SUMO (green), along with DNA. After treatment with auxin for 4 hr, BUB-1 is still present in the midbivalent. The scale bar represents 1 μm.

While we propose that SUMO affects KLP-19 directly, this interpretation is complicated by the fact that BUB-1, required for KLP-19 recruitment, is absent from the RC upon GEI-17 depletion. To overcome this, we used CRISPR/Cas9-mediated genome editing to tag the endogenous copies of *gei-17* with a fragment encoding a fusion between GFP, FLAG, and an auxin-responsive degron sequence (Zhang et al., 2015) (Figure 5A). Addition of auxin for 1.5 hr leads to the loss of the GFP-GEI-17 signal from the germline (Figure 5B) and embryo (Figure 5C) in worms specifically co-expressing mRuby-TIR1 (Zhang et al., 2015). Addition of auxin for 4 hr was sufficient to inhibit recruitment of KLP-19 to the midbivalent ring (Figures 5D and 5E) even though BUB-1 was still present (Figure 5F). However, re-scaling the SUMO channel fluorescence revealed that a small amount of SUMO could still be detected in ring-like structures, consistent with the persistence of other substrate(s) and/or non-covalent SUMO binding proteins (Figure 5E). Thus, sumoylation directly regulates KLP-19 recruitment to the midbivalent ring.

### Ring Components Are Recruited in a Stepwise Fashion from Diakinesis to Prometaphase

**Figure 6:**
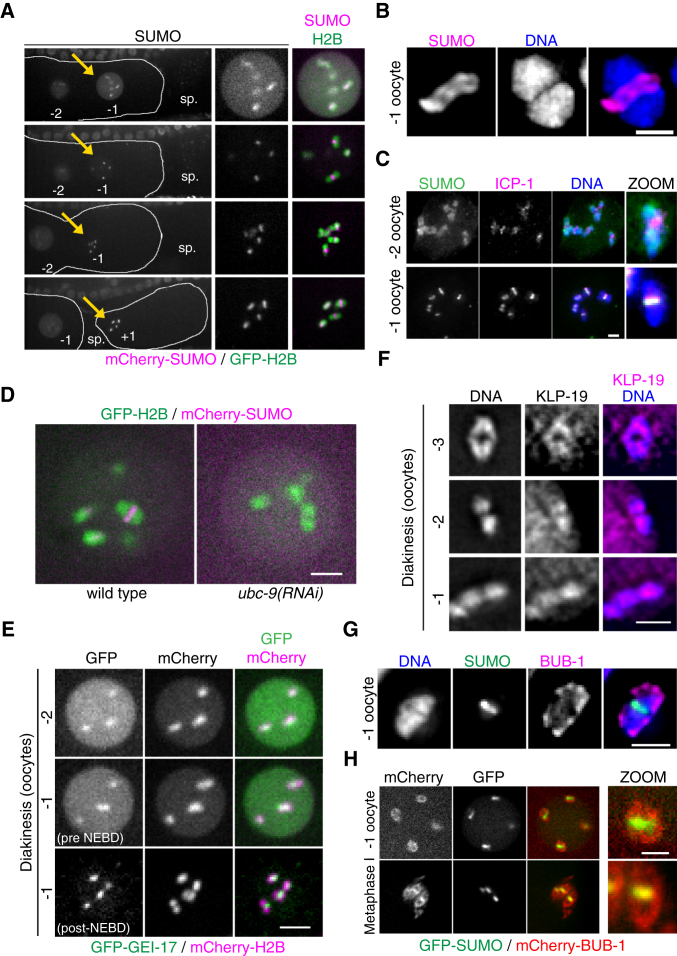
Ring Complex Assembly Starts during Diakinesis (A) An oocyte from a worm expressing mCherry-SUMO and GFP-H2B was recorded during fertilization. The yellow arrow in each image points to the oocyte that was followed. “−2” and “−1” stand for, the −2 and −1 oocytes, respectively; spermatheca, sp.; “+1” is the fertilized oocyte. (B) SUMO concentrates in the short axis of the bivalent (midbivalent). A bivalent within a −1 oocyte is shown with SUMO in magenta and DNA in blue. The scale bar represents 1 μm. (C) The CPC component ICP-1 (magenta) localizes to the midbivalent as early as the −2 oocyte, as opposed to SUMO (green) that only concentrates in the midbivalent in the −1 oocyte. The scale bar represents 2 μm. (D) SUMO concentration in the midbivalent in early oocytes is dependent on UBC-9. The −1 oocyte was followed as in (A) in wild-type or *ubc-9(RNAi)* worms. An image after NEBD is shown. The scale bar represents 2 μm. (E) GEI-17 concentrates on the midbivalent after oocyte NEBD. Worms expressing endogenous GEI-17 tagged with a GFP-FLAG-degron cassette together with mCherry-H2B were analyzed as in (A) and (E). The scale bar represents 5 μm. (F) KLP-19 (magenta) localization during diakinesis was analyzed by immunostaining of dissected gonads. The single bivalents from the three most mature oocytes are shown. The scale bar represents 2 μm. (G) BUB-1 is first recruited to the kinetochores. BUB-1 (magenta) localization along with that of SUMO (green) was analyzed by immunostaining of dissected gonads. A single bivalent from the −1 oocyte is shown. The scale bar represents 2 μm. (H) An oocyte from worms expressing GFP-SUMO and mCherry-BUB-1 was followed as in (A). In the upper image, the −1 oocyte has gone through NEBD (as judged by the SUMO staining), while the lower image shows bivalents in metaphase of meiosis I. The scale bar represents 1 μm.

Live imaging and immunostaining revealed that SUMO shifted from diffusely chromosomal to the midbivalent concomitant with oocyte NEBD (Figures 6A and 6B). Whereas ICP-1 was detected at the midbivalent in the −2 oocyte (Bishop and Schumacher, 2002, Schumacher et al., 1998), SUMO only concentrated in the midbivalent in the −1 oocyte (Figure 6C). This concentration of SUMO was dependent on conjugation as it was abrogated by *ubc-9(RNAi)* (Figure 6D). GEI-17 was also recruited to the midbivalent in post-NEBD oocytes, suggesting that GEI-17-mediated sumoylation within the ring was initiated during fertilization (Figure 6E) and precedes KLP-19 concentration in the midbivalent (Figure 6F) (Powers et al., 2004). In fixed −1 oocytes, BUB-1 is predominantly localized in kinetochores (Figure 6G), while live imaging on oocytes expressing mCherry-BUB-1 and GFP-SUMO showed that BUB-1 was initially recruited to the kinetochore, while SUMO was already concentrated in the midbivalent (Figure 6H, top image). By metaphase I, however, BUB-1 has been recruited to the RC (Figure 6H, bottom image). Thus SUMO modification of RC components precedes BUB recruitment to the midbivalent. These results show that the RC is assembled in a stepwise manner with assembly initiated prior to fertilization.

### BUB-1 Interacts with SUMO-Modified KLP-19 and GEI-17

**Figure 7:**
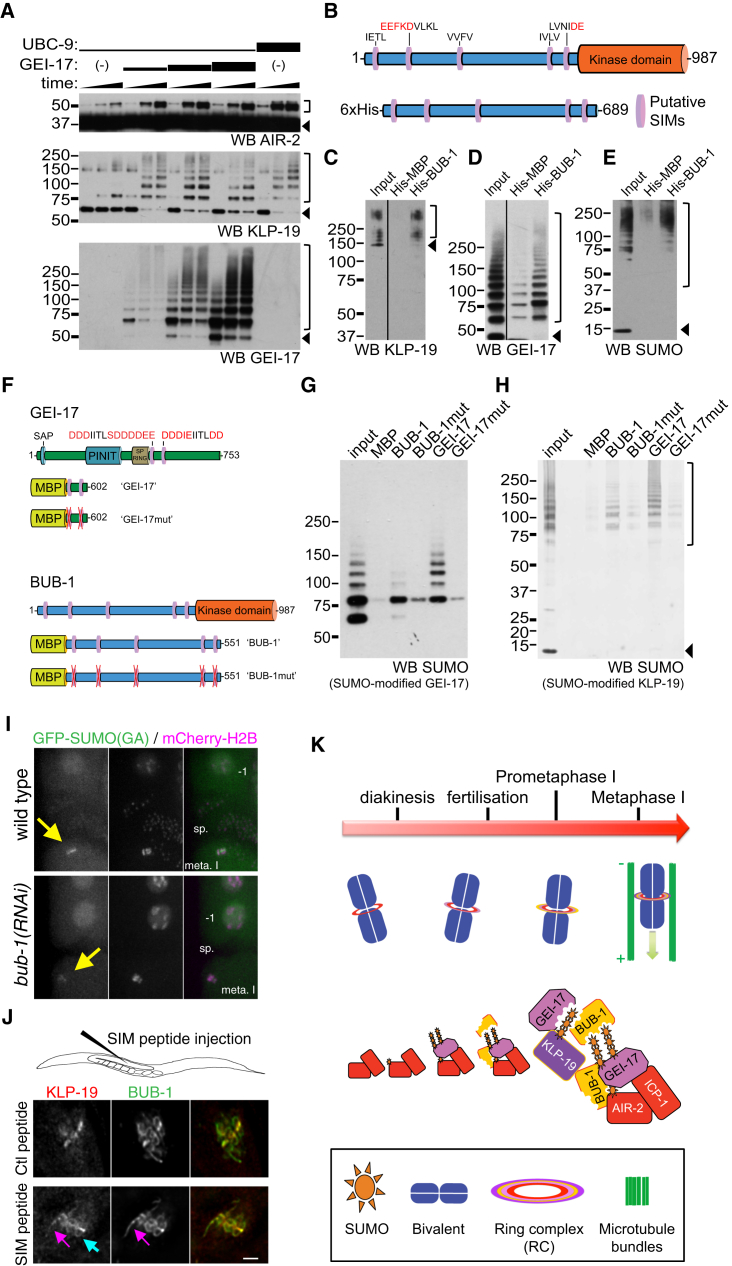
BUB-1 Binds to SUMO-Modified KLP-19 and GEI-17 (A) Reconstituted in vitro sumoylation reactions were performed as described in Experimental Procedures. While AIR-2 is modified by a single SUMO (doublet corresponds to two mono-sumoylated forms; Pelisch et al., 2014), KLP-19 and GEI-17 undergo multiple modifications. The arrowheads point to unmodified substrates, while open brackets indicate the SUMO-modified substrate. (B) Schematic of BUB-1 with its C-terminal kinase domain (top) and the hexahistidine-tagged BUB-1 fragment used for the pull-down assays shown in images (C)–(E). (C) 6xHis-MBP (“His-MBP”, control) and 6xHis-BUB-1(1–689) (“His-BUB-1”) were used in Ni-NTA pull-down assays using SUMO-modified full-length KLP-19 as input. The binding reactions were run on SDS-PAGE, and western blotting was performed with an antiKLP-19 specific antibody. The arrowhead points to the position of unmodified KLP-19, and the square bracket show the bands corresponding to SUMO-modified KLP-19. (D) Same as (C), but using SUMO-modified GEI-17 as input material. In this case, a GEI-17 specific antibody was used. The arrowhead points to the position of unmodified GEI-17, and the square bracket show the bands corresponding to SUMO-modified GEI-17. (E) Pull-down assays were performed using GEI-17-mediated SUMO-modified full-length KLP-19 as input. The reactions were analyzed as above using a SUMO-specific antibody. The arrowhead points to the position of unconjugated SUMO, and the square bracket indicates the position of conjugated SUMO (to KLP-19 and GEI-17). (F) Schematic of the GEI-17 and BUB-1 fragments containing the putative SIM motifs used in the pull-down experiments. (G) SUMO-modified GEI-17 was used for pull-down assays with MBP-tagged BUB-1, BUB-1mut, GEI-17, and GEI-17mut. Input and pulled-down materia were analyzed by western blot with an anti-SUMO antibody. (H) SUMO-modified KLP-19 was used for pull-down assays with MBP-tagged BUB-1, BUB-1mut, GEI-17, and GEI-17mut. Input and pulled-down materia were analyzed by western blot with an anti-SUMO antibody. The arrowhead indicates the presence of free SUMO, and the square bracket denotes SUMO-conjugated KLP-19(651–1,083). (I) Meiosis was followed in utero in control (wild-type) or *bub-1(RNAi)* worms expressing GFP-SUMO(GA) and mCherry-tagged H2B. The oocytes at metaphase I are pointed out by a yellow arrow. “sp.” denotes the location of the spermatheca; “meta. I” indicates the location of the oocyte at metaphase I; and “−1” shows the location of the maturing oocyte closest to the spermatheca before fertilization, the −1 oocyte. (J) *emb-27* worms were injected with a SIM-containing or a control peptide, and KLP-19 and BUB-1 localization was assessed in metaphase I-arrested oocytes. The pink arrow points to a linear element, and the blue arrow points to a cup-shaped kinetochore. The scale bar represents 2 μm. (K) Proposed model for SUMO-mediated control of chromosome congression.

The observation that a mutant SUMO incapable of conjugating to substrates partially localizes to the RC points to the existence of non-covalent SUMO interactions occurring within the RC. Having shown that KLP-19, AIR-2, and GEI-17 are conjugated to SUMO (Figure 7A), we searched for an RC component that could interact non-covalently with SUMO. An obvious candidate is the SUMO E3 ligase GEI-17, whose yeast and mammalian orthologs have SIMs (Jentsch and Psakhye, 2013). Another candidate is BUB-1, a protein that localizes in kinetochores and the RC and is essential for KLP-19 recruitment to the RC. Inspection of BUB-1 amino acid sequence revealed putative SIMs, mostly concentrated outside of the C-terminal kinase domain (Figure 7B). We expressed the fragment (1–689) containing the putative SIMs with a His_6_ tag to perform pull-down assays (Figure 7B). BUB-1(1–689) preferentially interacts with SUMO-modified KLP-19 (Figure 7C) and GEI-17 (Figure 7D). When analyzing binding reactions with a SUMO antibody, it was apparent that BUB-1 binds high molecular weight SUMO conjugates, but not free SUMO (Figure 7E). We then tested the functionality of the putative SIMs in BUB-1 and GEI-17 using MBP-fusion proteins (Figure 7F) in pull-down assays with sumoylated GEI-17 or KLP-19(651–1,083). While BUB-1(2–551) readily interacted with SUMO-modified GEI-17 and SUMO-modified KLP-19, mutation of all five putative SIMs abolished this interaction (Figures 7G and 7H). Additionally, a fragment containing the two predicted high-affinity SIMs in GEI-17 (aa 423–602 in isoform f), pulled down higher molecular weight forms of both sumoylated GEI-17 and sumoylated KLP-19 and these interactions were strictly SIM-dependent (Figures 7G and 7H). Thus, both SUMO substrates and non-covalent SUMO binders co-exist within the ring. If BUB-1 is important for non-covalent SUMO binding in vivo, then depletion of BUB-1 is predicted to affect non-covalent SUMO recruitment to the RC. To test this, we used a mutant version of SUMO that cannot conjugate to substrate proteins (SUMO(GA), see Figures 2J and 2K) and thus provides a readout for non-covalent SUMO interactions. Consistent with BUB-1 interacting non-covalently with SUMO/sumoylated proteins, GFP-SUMO(GA) recruitment to the RC during meiosis I was greatly diminished in the absence of BUB-1 (Figure 7I, yellow arrows). We then sought to test whether KLP-19 localization in the RC is SIM dependent. To this end, we injected a SIM containing peptide (Bruderer et al., 2011) (or a control peptide) into the gonads of *emb-27* worms and metaphase I-arrested oocytes were analyzed 24 hr later (Figure 7J). While SIM injection did not dramatically affect KLP-19 recruitment to the RC, an increase in KLP-19 in kinetochores and linear elements was observed (Figure 7J). This result, while smaller in magnitude, resembles the effect obtained after knocking down either GEI-17 or UBC-9 (Figures 3I–3K). Thus, we conclude that both covalent SUMO conjugation and non-covalent SUMO interaction contribute to stable RC assembly. Notably, the dynamic and reversible nature of these interactions guarantees that the ring can be easily disassembled, an event required as anaphase progresses.

## Discussion

We provide evidence that SUMO modification plays an important role during female meiotic chromosome congression in *C. elegans* by regulating RC assembly (Figure 7K). AIR-2/Aurora B and ICP-1/INCENP localize to the midbivalent during diakinesis in a SUMO-independent manner, providing the basic platform of the RC. The SUMO E3 ligase GEI-17 then joins the complex during oocyte nuclear envelope breakdown and triggers SUMO conjugation, likely of AIR-2 (and/or other yet to be identified RC components). Then BUB-1, which is already in kinetochores, joins the RC in a GEI-17-dependent manner. Finally, the kinesin KLP-19 is modified by SUMO in a GEI-17-dependent manner and is recruited to the midbivalent RC in a sumoylation-dependent fashion. As the SIMs in both GEI-17 and BUB-1 allow them to interact with SUMO-modified GEI-17 and KLP-19 (and likely other SUMO-modified proteins), they act as central players in the formation of this meiosis-specific SUMO-SIM network.

Both SUMO conjugation and non-covalent SUMO interaction are required for proper RC assembly. Indeed, when sumoylation is inhibited by UBC-9 or GEI-17 depletion, KLP-19 “diffuses away” from the RC and displays a localization pattern characteristic of outer kinetochore proteins, localizing in cup-shaped structures surrounding the bivalents and in linear elements in the spindle and cell cortex (Dumont et al., 2010, Monen et al., 2005). In this model, SUMO could be key in regulating the partitioning of proteins between the RC and other neighboring structures.

Many aspects of the RC function during meiosis remain to be elucidated. This structure is functioning as a signaling hub, where phospho-proteins, as well as SUMO-modified proteins concentrate. The enzymes that catalyze these modifications, like the kinases AIR-2/Aurora B and BUB-1 and the SUMO E3 ligase GEI-17, localize within the RC themselves. This suggests that active protein modification takes place within the RC. The RC was shown to disassemble during anaphase, so future studies are needed to address the role of SUMO proteases in RC disassembly. Interestingly, it was recently reported that protein phosphatase 1 recruitment by the nucleoporin MEL-28 directs outer kinetochore disassembly, an event required for proper meiotic chromosome segregation (Hattersley et al., 2016). We propose that PTMs, and interactions among them, will be key regulators of the highly dynamic changes that take place within the meiotic spindle.

A remarkable feature of the RC is that within a 30-min period, it undergoes two cycles of assembly/disassembly linked to two waves of SUMO modification/deconjugation that are regulated with exquisite precision both temporally and spatially. During oocyte nuclear envelope breakdown, RC becomes SUMO modified and an assembly feedforward cycle starts. However, SUMO is removed and the RC disassembles during anaphase I, and this is followed by SUMO conjugation/RC assembly during prometaphase II and SUMO deconjugation/RC disassembly during anaphase II. However, this is not the end of the dynamic behavior, as we have shown that SUMO modification/deconjugation takes place during the first embryonic mitotic division (Pelisch et al., 2014). The ability of SUMO to function as a reversible molecular “glue” satisfies the need for this rapid assembly-disassembly cycles. Since the introduction of the protein group sumoylation concept (Psakhye and Jentsch, 2012), SUMO-mediated RC assembly provides the first example of a specialized complex within a multi-cellular organism assembled as a SUMO-SIM network under physiological conditions. Just as a balance of forces exists between kinesin-driven plus-end movement and dynein-mediated minus end forces (Muscat et al., 2015), a similar equilibrium may be mediated by SUMO E3 ligases and SUMO proteases. Indeed, the SUMO protease ULP-1 localizes to the RC (data not shown). In this context, while E3 activity prevails until metaphase, SUMO proteases are likely to predominate during anaphase, leading to ring disassembly. While it remains to be shown what signal(s) regulate the balance between E3 and protease activities, the presence of both E3 ligases and SUMO proteases would facilitate the assembly/disassembly cycles.

In budding yeast, SUMO co-localizes with the synaptonemal complex during pachytene and plays a role in chromosome synapsis (Cahoon and Hawley, 2016). However, SUMO is not essential for SC assembly in early pachytene in nematodes (Bhalla et al., 2008) and localizes mainly at the midbivalent, where key regulators of chromosome congression (like KLP-19) reside. As in nematodes, it has been shown in rat spermatocytes that SUMO does not co-localize with the synaptonemal complex during pachytene (Rogers et al., 2004), while depletion of SUMO or UBC9 caused abnormal spindle organization, and led to chromosome misalignment, segregation defects, and aneuploidy in rat oocytes (Yuan et al., 2014). Additionally, SUMO-1 concentrates in spindle poles and between segregating chromosomes in anaphase I, while SUMO-2/3 co-localized with condensed chromatin in mouse oocytes (Wang et al., 2010). This localization pattern is reminiscent of that of SUMO-1 and SUMO-2/3 during mitosis (Zhang et al., 2008), and the only SUMO isoform in nematodes displays a combination of these localization patterns during the first embryonic mitotic division (Pelisch et al., 2014). Many SUMO substrates are involved in chromatin structure and function (Cubeñas-Potts and Matunis, 2013), and KIF4A, the human ortholog of KLP-19, has been identified as a SUMO substrate in mitotic chromatin (Cubeñas-Potts et al., 2015). These observations support the notion that while precise mechanisms that guarantee proper chromosome orientation, congression, and segregation might differ between meiosis and mitosis and also among species, SUMO is likely a key contributor to a timely and accurate regulation of protein interactions within narrow spatial and temporal windows. In line with this, AIR-2/Aurora B shifts its localization from chromatin to the spindle midzone during mitotic anaphase and this transition is dependent on the SUMO protease ULP-4 (Pelisch et al., 2014). SUMO-SIM networks are likely to predominate when the equilibrium of a protein between two or more cell structures/protein complexes is subject to a fast dynamic regulation. Overall, we have provided evidence that highly dynamic, coordinated, and spatially constrained sumoylation regulates chromosome congression during meiosis in *C. elegans* oocytes.

## Experimental Procedures

### Worms

*C. elegans* strains were maintained according to standard procedures (Brenner, 1974). The strains used are listed in Supplemental Experimental Procedures. For RNAi treatment, bacterial clones expressing dsRNAs were obtained from a commercial library (Kamath and Ahringer, 2003).

### CRISPR/Cas9

GEI-17 fused to GFP-FLAG-degron was generated by CRISPR (Dickinson et al., 2015). The degron sequence consisted of the 44-amino acid (aa) fragment of the *Arabidopsis thaliana* IAA17 protein (Morawska and Ulrich, 2013, Zhang et al., 2015).

### Auxin Treatment

Auxin (IAA, Sigma-Aldrich) was used at 1 mM final concentration in standard NGM plates.

### Antibodies

Antibodies against SMO-1, GEI-17, and UBC-9 were reported previously (Pelisch and Hay, 2016, Pelisch et al., 2014). AIR-2 and ICP-1 peptide antibodies were produced and affinity purified using previously described peptides (Burrows et al., 2006, Schumacher et al., 1998). Anti-KLP-19 serum (Powers et al., 2004) was subject to protein-A purification before use.

### In Utero Embryo Live Imaging

Worms were picked into a solution of tricaine (0.1%) and tetramisole (0.01%), pipetted onto a 4% agar pad, covered with a coverslip, and imaged with a spinning-disk confocal microscope (MAG Biosystems) mounted on a microscope (IX81; Olympus) with a 100 × /1.45 Plan Apochromat oil immersion lens (Olympus), a camera (Cascade II; Photometrics), spinning-disk head (CSU-X1; Yokogawa Electric), and MetaMorph software (Molecular Devices).

### Immunostaining

Immunofluorescence analysis was performed essentially as described (Pelisch and Hay, 2016, Pelisch et al., 2014).

### Duolink In Situ Proximity Ligation Assay

Proximity ligation assays were performed as described (Pelisch and Hay, 2016, Pelisch et al., 2014).

### Analysis of Mass Spectrometry Data

Raw mass spectrometry (MS) files were analyzed using MaxQuant software package (version 1.3.0.5) (Cox and Mann, 2008) and peak lists were searched with an integrated Andromeda search engine (Cox et al., 2011) against an entire *C. elegans* UniProtKB proteome.

### Statistical Analysis

The different tests used throughout the study are detailed in Supplemental Experimental Procedures.

## Author Contributions

F.P. conceived the project; designed, performed, and interpreted experiments; and wrote the manuscript. T.T. performed the diGly peptide purification experiments and mass spectrometry analysis. B.W. performed injections. E.G.J. purified recombinant proteins for pull-down assays. A.G. interpreted experiments and revised the manuscript. R.T.H. designed and interpreted experiments, supervised the project, and wrote the manuscript.

## Supplemental Information

Document S1. Supplemental Experimental Procedures and Figures S1–S7

Document S2. Article plus Supplemental Information
