# Supplementary material for: A SUMO-Dependent Protein Network Regulates Chromosome Congression during Oocyte Meiosis
Source: Mol Cell. 2017 Jan 5;65(1):66–77. doi: 10.1016/j.molcel.2016.11.001 (PMC5222697; doi:10.1016/j.molcel.2016.11.001)
Supplement: Document S1. Supplemental Experimental Procedures and Figures S1–S7 [file mmc1.pdf]

Molecular Cell, Volume *65*

Supplemental Information

A SUMO-Dependent Protein Network Regulates Chromosome Congression during Oocyte Meiosis

Federico Pelisch, Triin Tammsalu, Bin Wang, Ellis G. Jaffray, Anton Gartner, and Ronald T. Hay

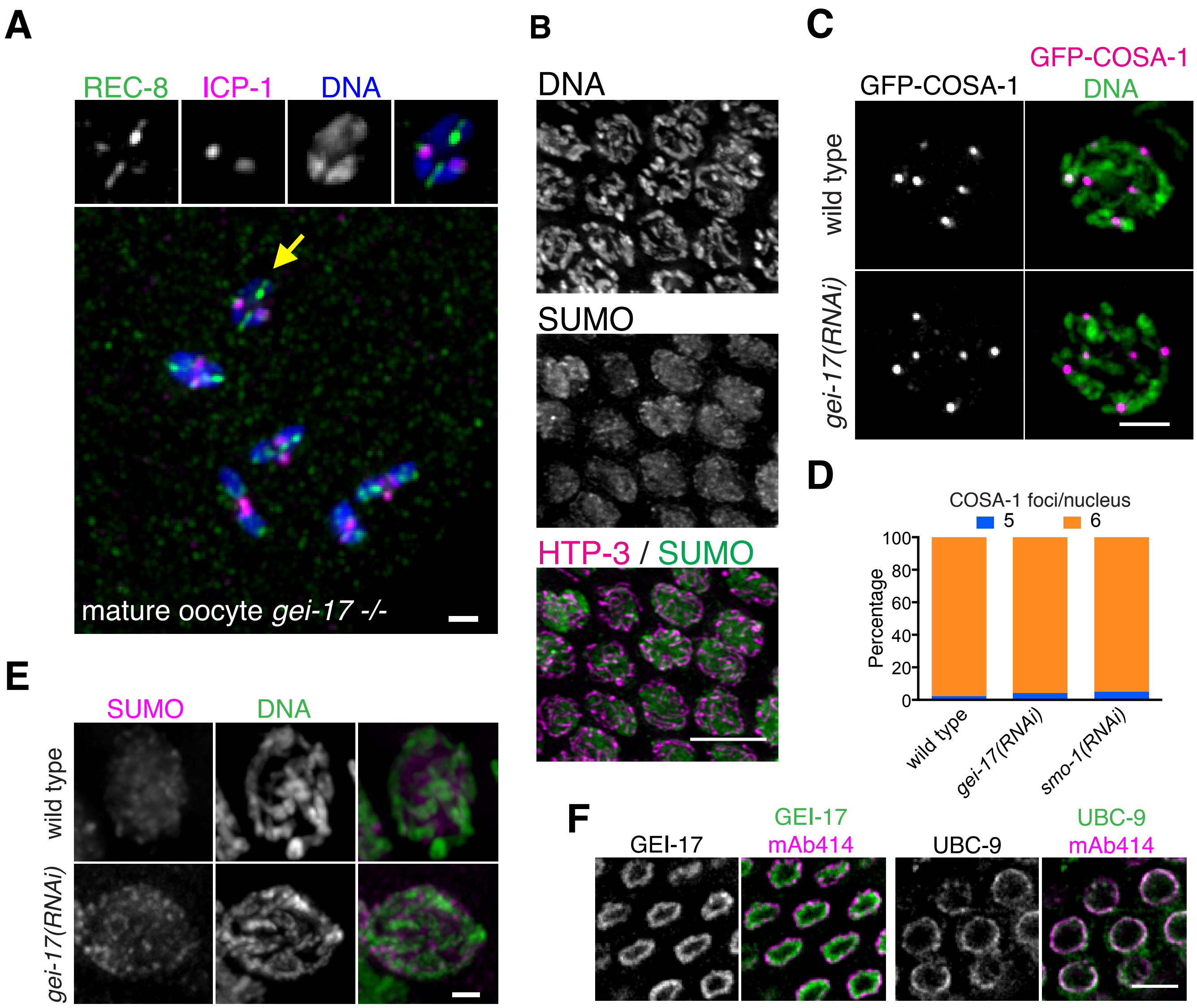
A
REC-8
ICP-1
DNA
mature oocyte gei-17 -/-
B
DNA
SUMO
HTP-3 / SUMO
C
GFP-COSA-1
GFP-COSA-1
DNA
wild type
gei-17(RNAi)
D
COSA-1 foci/nucleus
5
6
Percentage
100
80
60
40
20
0
wild type
gei-17(RNAi)
smo-1(RNAi)
E
SUMO
DNA
wild type
gei-17(RNAi)
F
GEI-17
GEI-17
mAb414
UBC-9
UBC-9
mAb414

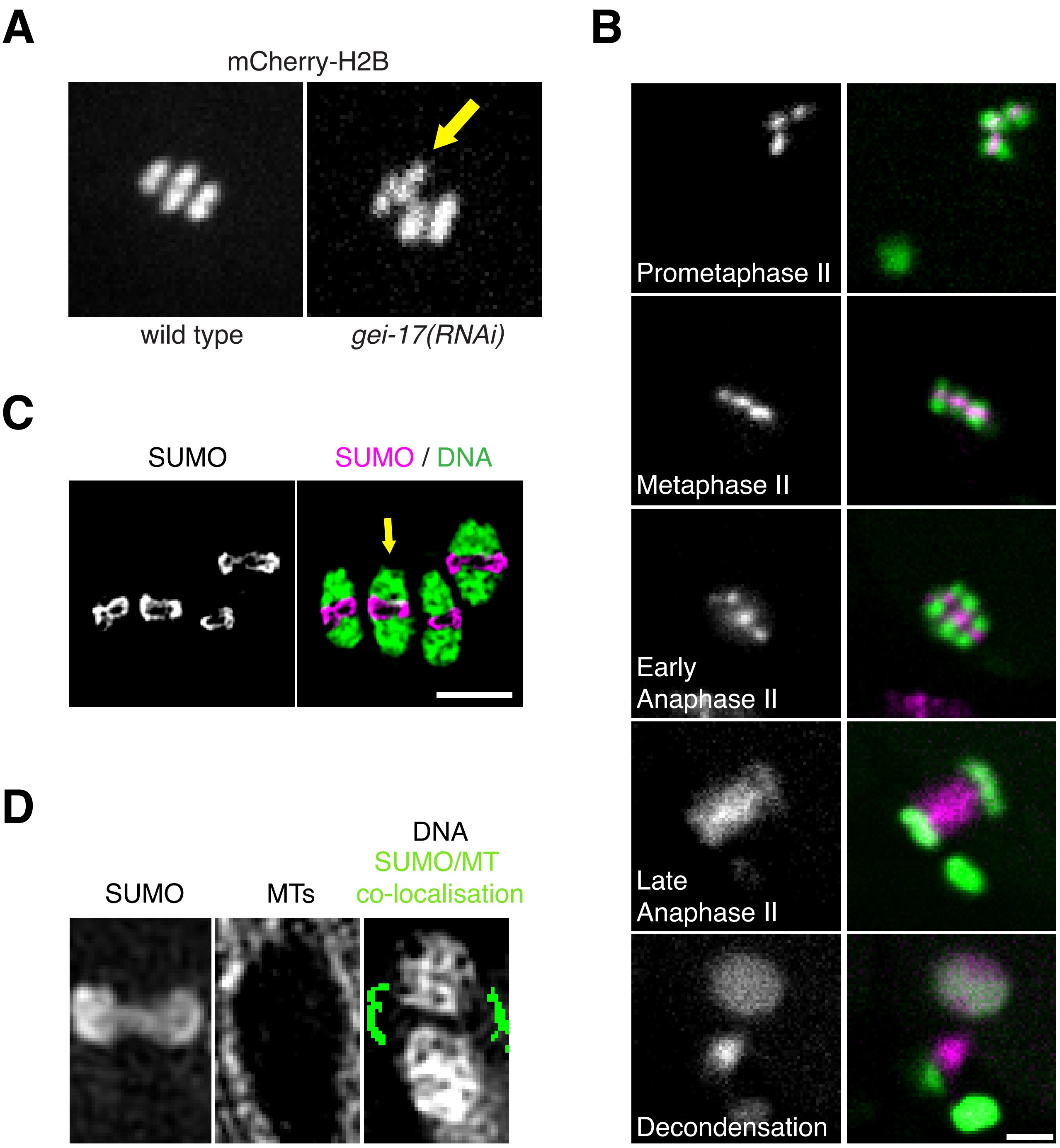
A
mCherry-H2B
wild type
gei-17(RNAi)
B
Prometaphase II
Metaphase II
Early
Anaphase II
Late
Anaphase II
Decondensation
C
SUMO
SUMO / DNA
D
SUMO
MTs
DNA
SUMO/MT
co-localisation

Pelisch et al. Figure S3

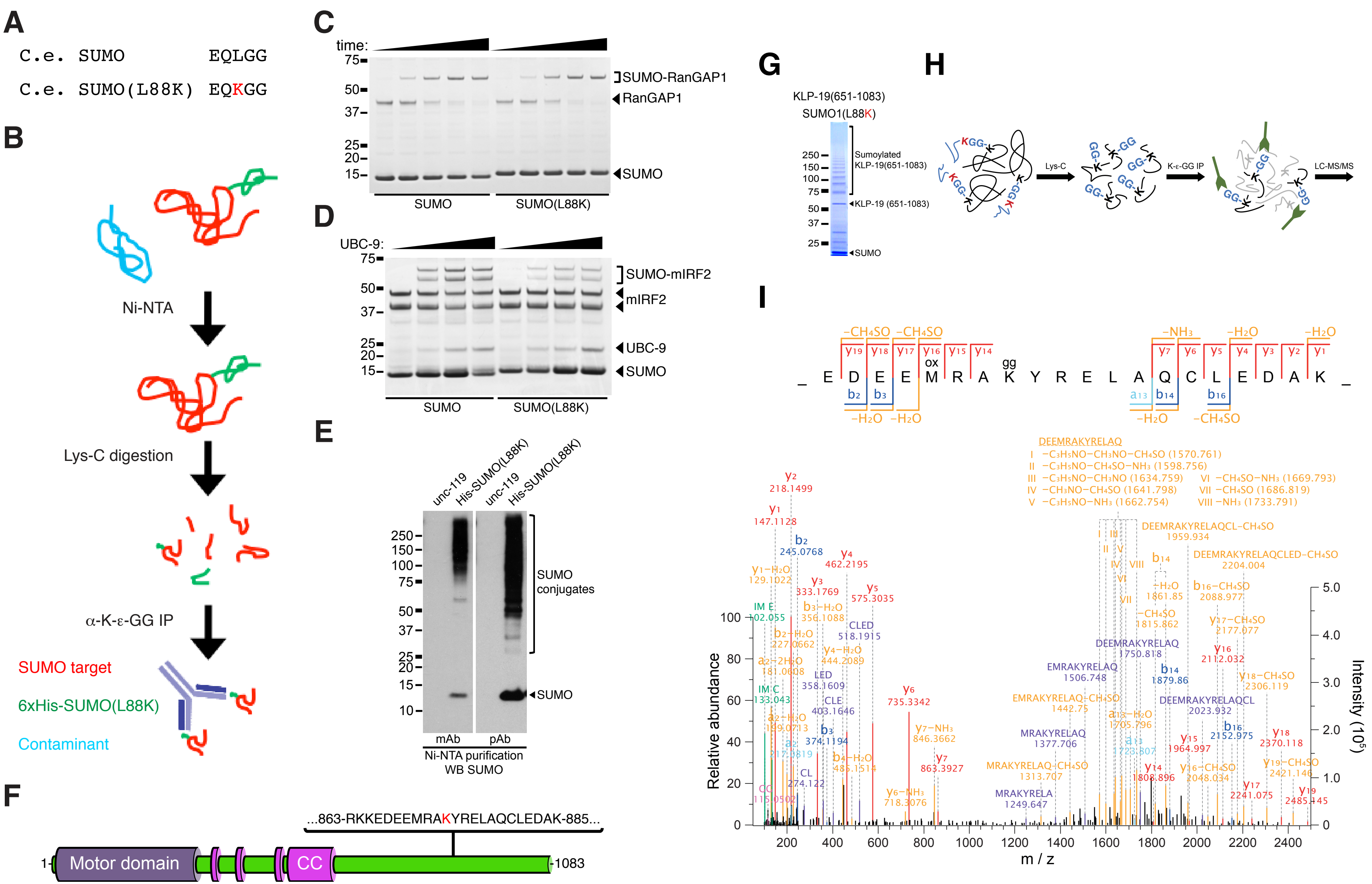

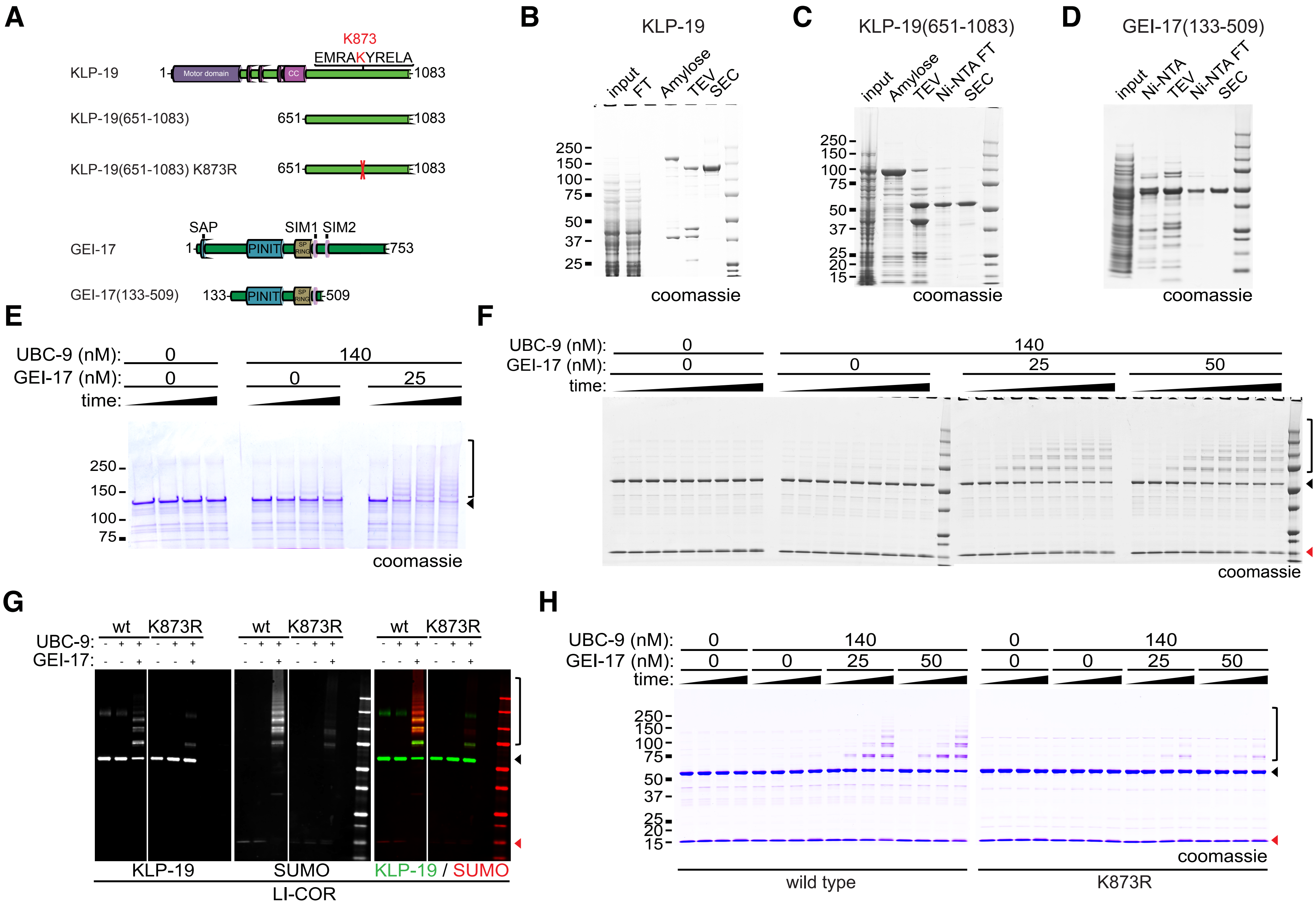
A
K873
EMRAKYRELA
KLP-19
Motor domain
CC
1083
KLP-19(651-1083)
651
KLP-19(651-1083) K873R
SAP
SIM1 SIM2
GEI-17
PINIT
753
GEI-17(133-509)
133
509
B
KLP-19
input FT Amylose TEV SEC
coomassie
C
KLP-19(651-1083)
input Amylose TEV Ni-NTA FT SEC
D
GEI-17(133-509)
input Ni-NTA TEV Ni-NTA FT SEC
E
UBC-9 (nM):
GEI-17 (nM):
time:
F
G
wt K873R
UBC-9:
GEI-17:
KLP-19
SUMO
KLP-19 / SUMO
LI-COR
H
wild type
K873R

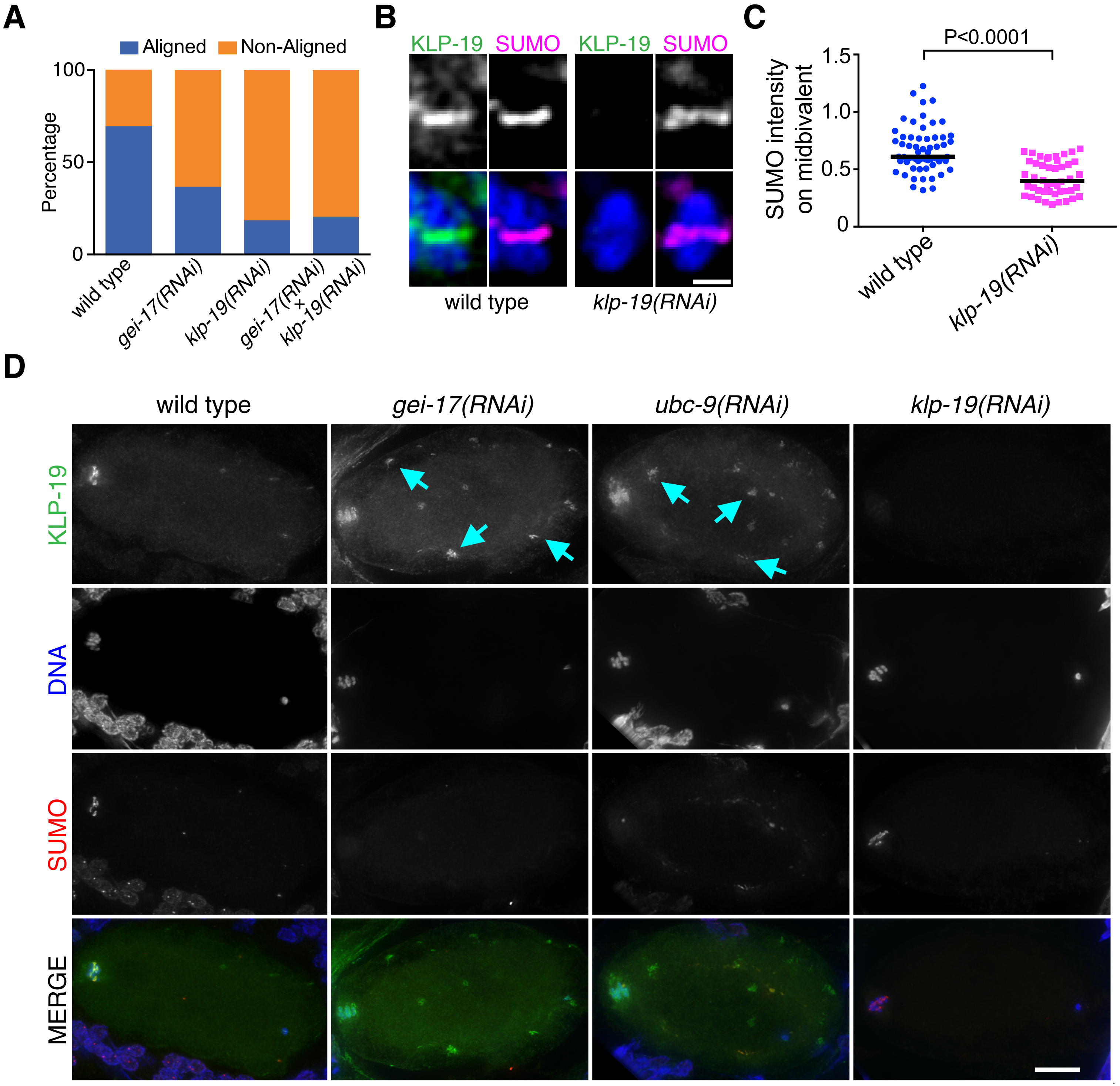
A
Aligned
Non-Aligned
100
50
0
Percentage
wild type
gei-17(RNAi)
klp-19(RNAi)
gei-17(RNAi) x klp-19(RNAi)
B
KLP-19
SUMO
KLP-19
SUMO
wild type
klp-19(RNAi)
C
P<0.0001
1.5
1.0
0.5
0
SUMO intensity on midbivalent
wild type
klp-19(RNAi)
D
wild type
gei-17(RNAi)
ubc-9(RNAi)
klp-19(RNAi)
KLP-19
DNA
SUMO
MERGE

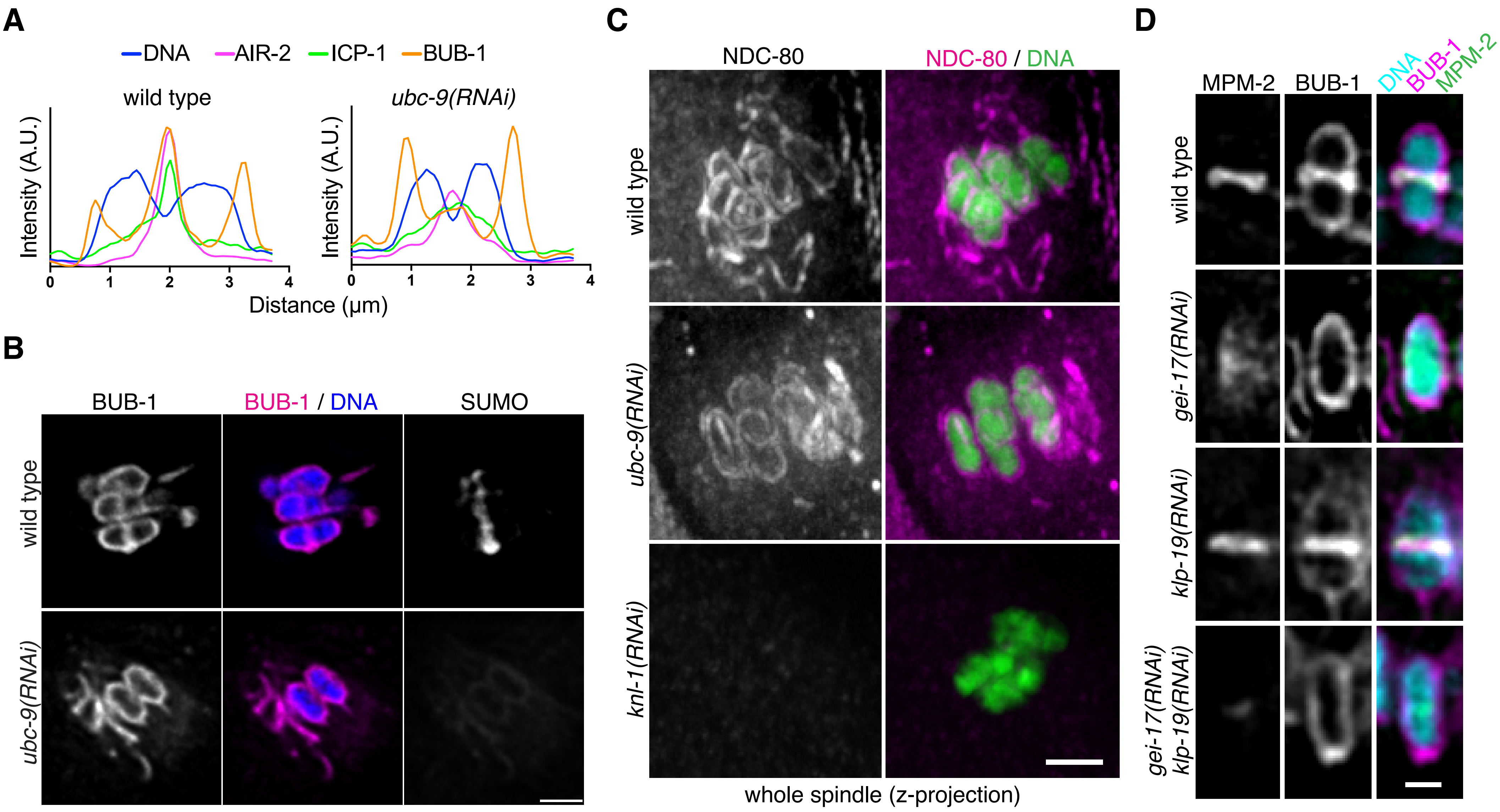
A
DNA
AIR-2
ICP-1
BUB-1
wild type
ubc-9(RNAi)
Intensity (A.U.)
Distance (µm)
0
1
2
3
4
B
BUB-1
BUB-1 / DNA
SUMO
wild type
ubc-9(RNAi)
C
NDC-80
NDC-80 / DNA
wild type
ubc-9(RNAi)
knl-1(RNAi)
whole spindle (z-projection)
D
MPM-2
BUB-1
DNA
BUB-1
MPM-2
wild type
gei-17(RNAi)
klp-19(RNAi)
gei-17(RNAi)
klp-19(RNAi)

Pelisch et al. Figure S7

SMO-1

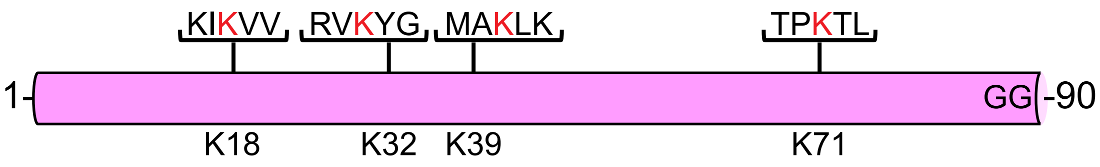


```
1-    MADDAAQAGD    NAEYIKIKVV    GQDSNEVHFR    VKYGTSMAKL
41-   KKSYADRTGV    AVNSLRFLFD    GRRINDDDTP    KTLEMEDDDV
81-   IEVYQEQLGG    F
```

GEI-17 (isoform f)

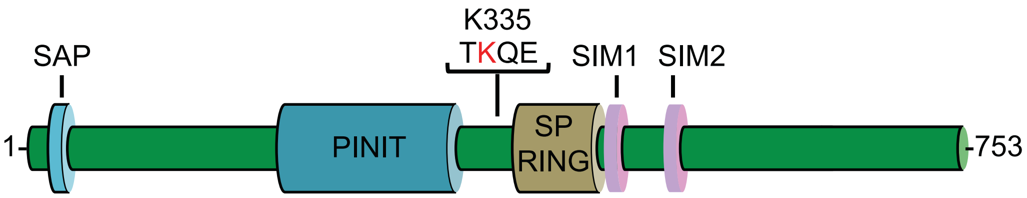


```
133-   QQMMASHHSH    LQQQHHPSTP    KKMYADNFEP    LPLPFYDVIS
173-   VLLKPVELHS    SDSPTLKQTK    QLQFPFLLTA    EHISKISYRA
213-   DVTPLPRYEL    QLRFFNLTEP    VQGPQKDDFP    LNCYARVDDS
253-   VVQLPNVIPT    NKTNAEPKRP    SRPVNITSNM    NRYKKEHTVA
293-   VEWLADKRVW    AAGVYFVHRV    NSDILFKRLN    QNVSRHRSLE
333-   VTKQEVIKKL    SGGEDDIAMD    RLNISLLDPL    CKTRMTTPSR
373-   CQDCTHLQCF    DLLSYLMMNE    KKPTWQCPVC    SSNCPYDRLI
413-   VDDYFLDMLA    KVDKNTTEVE    LKEDGSYDVI    KEEAFCISDD
453-   DDDDVVPATV    NGTASCSSTN    GNGLANEAAK    KKPADDDIIT
493-   LSDDDDEELN    RGIMNSL
```

KLP-19

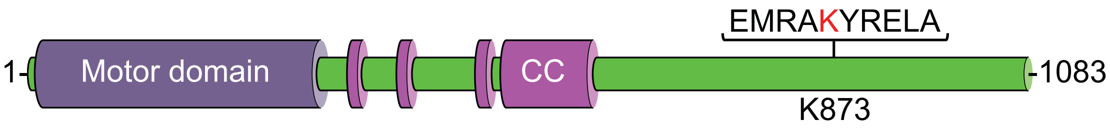


```
651-    VAIQKRMTDQ    KLTVLQMRLT    EANRANKTLR    ELNLKRANRK
691-    SSPTNASALQ    NMIEEELEHE    MCAQRSHWLC    EDLRRQRHDL
731-    MQNINTVESM    KFEGGKRRRI    SASADPNVSV    VIEGEEEFEV
771-    KRQKELTFLR    ASLETLNEEI    KDSLRNETIA    GNEERANSRW
811-    EKVPAEMRPA    FEAVYAQAVA    HIRKEIELEF    KLARTKSEFT
851-    AKIASKASHE    EKRKKEDEEM    RAKYRELAQC    LEDAKSGLHE
891-    KIAFLLCLIK    ENRVDENAIQ    QFESLKNQFC    DVEQKVKKAS
931-    RRKTTNFMGG    LTPKPELQRN    ERARRAVKYY    GNVVNSEDVT
971-    MDDSRHQKRK    DHSLLAVEMN    RTTDDNVKRR    VAMSPIKCDD
1011-   DTRLTEEDED    IENEAMNNAT    FVKDSFNSAT    IVLDDSQPSP
1051-   SNSTFVIGAA    PTSEADGVPP    IKRKSRRTDL    GPL
```

Supplemental Figure Legends

Figure S1 (related to Figure 1). *C. elegans* sumoylation pathway in pachytene.
A. An oocyte from a *gei-17 -/-* was stained with ICP-1 in magenta, REC-8 in green, and DNA in blue. The bottom panel corresponds to a zoomed image of the bivalent marked with a yellow arrow. Scale bar, 1 µm.
B. Nuclei in the pachytene stage of meiosis showing SUMO localisation in green and HTP-3 in magenta. Scale bar, 5 µm.
C. GFP-COSA-1 foci in late pachytene were counted from dissected gonads of wild type and *gei-17(RNAi)* worms. GFP-COSA-1 is shown in magenta and DNA in green. Scale bar, 2 µm.
D. Quantitation of COSA-1 foci in late pachytene in wild type, *gei-17(RNAi)*, or *smo-1(RNAi)* worms.
E. SUMO localisation pattern in pachytene nuclei was assessed in control ('wild type') and GEI-17-depleted worms ['*gei-17(RNAi)*']. Scale bar, 1 µm.
F. GEI-17 and UBC-9 localisation in pachytene nuclei was assessed by immunofluorescence. The nuclear pore complex marker mAb414 was used to co-stain the nuclear envelope. Scale bar, 5 µm.

Figure S2 (related to Figure 2).
A. Metaphase I images of oocytes expressing mCherry-H2B, either from wild type or *gei-17(RNAi)* worms. The yellow arrow points to one misaligned bivalent.
B. Meiosis II in worms expressing mCherry-SUMO/GFP-H2B was followed by in utero time lapse. Still images of the different stages are shown with SUMO coloured in magenta and H2B in green. Scale bar, 2 µm.
C. The image corresponds to a bigger field of view to that showed in Figure 2C. The bivalent shown in the Figure 2C is indicated by a yellow arrow. Scale bar, 2 µm.
D. 3D-SIM image of a meiosis I spindle region showing staining for SUMO, microtubules ('MTs') and their co-localisation, highlighted in green in the panel on the right.

Figure S3 (related to Figure 3). Mass spectrometry-based SUMO site identification strategy, setup, and results.
A. The C-terminus of the nematode processed SUMO is indicated, with the Leu to Lys substitution highlighted in red.
B. A schematic of the protocol used for the identification of sumoylation sites in vivo. Briefly, worms expressing 6xHis-SUMO(L88K) are lysed and a denaturing Ni-NTA purification is performed. After Lys-C digestion, GG-K-modified peptides are immunoprecipitated using an anti-K-e-GG specific antibody, before analysis by mass spectrometry.
C. A time course analysis of RanGAP1 sumoylation using wild type and L88K mutant SUMO. Proteins were visualised by coomassie staining.
D. mIRF2 sumoylation with wild type and L88K mutant SUMO was analysed using increasing UBC-9 concentrations.
E. Hexahistidine-tagged SUMO(L88K) is conjugated to substrates in vivo. Worms expressing 6xHis-SUMO(L88K) were lysed denaturing Ni-NTA chromatography was performed. Eluted material was analysed by western blotting using a monoclonal ('mAb') or a polyclonal ('pAb') anti-SUMO antibodies.
F. Diagram of KLP-19 highlighting the motor domain, the predicted coiled-coil domains (in pink, 'CC'), and the peptide identified as SUMO modified in vivo.
G. Recombinant KLP-19(651-1083) was SUMO modified in vitro and an aliquot of the reaction was run on an SDS-PAGE and analysed by coomassie staining.
H. Diagram of the workflow for identifying SUMO modification sites from in vitro reactions.
I. KLP-19 is sumoylated in vitro. A representative annotated MS/MS spectrum of diGly remnant-containing peptide of KLP-19 (Lysine 873 is modified). Fragment ions extending from the N-terminus of the peptide are named as b- (in dark blue) or a-ions (in light blue), and the individual a-b ion pairs have a mass difference of a carbonyl group (C=O, 27.995 Da). C-terminal y-ions of the peptide are illustrated in red. Peptide internal fragments, ions with a loss of neutral molecule(s) or immonium ions are reported in purple, yellow or green, respectively. Ions diagnostic for diGly modification are shown in pink. This annotated MS/MS spectrum has an Andromeda score of 104.86 with a posterior error probability of 5.63 x 10-9.

Figure S4 (related to Figure 3). Detailed in vitro analysis of KLP-19 sumoylation.

A. Schematic of the different KLP-19 and GEI-17 proteins and fragments used for in vitro SUMO modification assays.
B. Summary of the purification process of full-length KLP-19. The protein was expressed as a 6xHis-MBP fusion with a TEV protease site between the tag and KLP-19, and purified using amylose beads. After TEV cleavage, the protein was purified by size exclusion chromatography using a Superose 6 column. Input, soluble lysate; FT, flow-through of the amylose column; Amylose, maltose-eluted material; TEV, sample after TEV cleavage; SEC, sample after size exclusion chromatography.
C. Summary of the purification process of KLP-19(651-1083). The protein was expressed as a 6xHis-MBP fusion with a TEV protease site between the tag and KLP-19, and purified using amylose beads. After TEV cleavage, the 6xHis-MBP tag and the 6xHis-TEV were removed using a Ni-NTA column. The flow through (FT, containing KLP-19(651-1083)) was further purified by size exclusion chromatography using a Superdex 200 column. Input, soluble lysate; Amylose, maltose-eluted material; TEV, sample after TEV cleavage; Ni-NTA FT, Ni-NTA column flow-through of TEV treated sample; SEC, sample after size exclusion chromatography.
D. Summary of the purification process of GEI-17(133-509). The protein was expressed as a 6xHis fusion with a TEV protease site between the tag and GEI-17, and purified using Ni-NTA beads. After TEV cleavage, the 6xHis tag and the 6xHis-TEV were removed using a Ni-NTA column. The flow through (FT, containing untagged GEI-17) was further purified by size exclusion chromatography using a Superdex 200 column. Input, soluble lysate; Ni-NTA, imidazole-eluted material; TEV, sample after TEV cleavage; Ni-NTA FT, Ni-NTA column flow-through of TEV treated sample; SEC, sample after size exclusion chromatography.
E. KLP-19 was subject to in vitro sumoylation. Reactions contained 1 μM KLP-19, 20 μM SUMO, and the indicated amounts of UBC-9 and GEI-17 and were incubated for 0, 30, 60, and 120 minutes.
F. KLP-19(651-1083) was subject to in vitro sumoylation. Reactions contained 6 μM KLP-19, 20 μM SUMO, and the indicated amounts of UBC-9 and GEI-17 and were incubated for 0, 15, 30, 45, 60, 75, 90, 105, and 120 minutes. Red arrowhead, free SUMO. Black arrowhead, unmodified KLP-19. Square bracket, SUMO-modified KLP-19.
G. KLP-19(651-1083) and KLP-19(651-1083) K873R were subject to in vitro sumoylation. Reactions contained 3 μM KLP-19, 20 μM SUMO, 140 nM UBC-9, and 12.5 nM GEI-17. Reactions were incubated for 60 minutes and analysed by two-color western blotting using a LI-COR system. Red arrowhead, free SUMO. Black arrowhead, unmodified KLP-19. Square bracket, SUMO-modified KLP-19. Note that this is the same blot shown in Figure 3F, showing the individual channels in addition to the merged image.
H. KLP-19(651-1083) and KLP-19(651-1083) K873R were subject to in vitro sumoylation. Reactions contained 6 μM KLP-19, 20 μM SUMO, and the indicated amounts of UBC-9 and GEI-17 and were incubated for 0, 15, 30, and 60 minutes. Red arrowhead, free SUMO. Black arrowhead, unmodified KLP-19. Square bracket, SUMO-modified KLP-19.

Figure S5 (related to Figure 3).
A. Spindles were characterised as either 'aligned' or 'non-aligned' (at least one chromosome away from the metaphase plate) and results from wild type, *gei-17(RNAi)*, *klp-19(RNAi)*, and *gei-17(RNAi)* + *klp-19(RNAi)* oocytes are shown as % of total.
B. Metaphase I-arrested wild type or *klp-19(RNAi)* oocytes were stained for KLP-19, SUMO, and DNA as indicated.
C. Quantitation of the SUMO signal form the experiment shown in B. Results were analysed by a Mann-Whitney test and black lines indicate the median.
D. Whole oocyte corresponding to the spindle images shown in Figure 3I. The cyan arrows point to the cortical linear elements.

Figure S6 (related to Figure 4). RC but not kinetochores are affected in the absence of sumoylation.
A. Representative intensity profiles of single bivalents from wild type or *ubc-9(RNAi)* oocytes that stained with alexa-labelled AIR-2, ICP-1, and BUB-1 antibodies.
B. Single z slice from wild type or *ubc-9(RNAi)* oocytes stained for BUB-1 (magenta) and DNA (blue). Scale bar, 2 μm.
C. Localisation of the kinetochore component NDC-80 was analysed in the presence of control RNAi ('wild type'), *ubc-9(RNAi)*, or *knl-1(RNAi)*, a known regulator of kinetochore assembly. The images correspond to whole spindle, z-projections of the single bivalents shown in Figure 4C. Scale bar, 2 μm.

D. MPM-2 reactive antigens and BUB-1 localisation were analysed in control ('wild type'), *gei-17(RNAi)*, *klp-19(RNAi)*, *and gei-17(RNAi) + klp-19(RNAi)* in metaphase I-arrested oocytes. Scale bar, 1 μm.

Figure S7 (Related to Figure 3, see main text). SUMO conjugation sites identified in SUMO, GEI-17, and KLP-19.
Lysines found to be SUMO modified in GEI-17, KLP-19, or SUMO itself during in vitro assays.

Supplemental Experimental Procedures

Worms. *C. elegans* strains were maintained according to standard procedures (Brenner, 1974). Where indicated, transgenic worms were generated by particle bombardment (Praitis et al., 2001). To generate the GFP-tagged fusion protein, the respective full-length cDNAs were amplified from N2 worms and cloned into PIE-1 regulatory element (Wallenfang and Seydoux, 2000) in a pIC26 vector (Cheeseman and Desai, 2005). The complete set of strains used in the present paper is listed below. Please note that plasmid names in FGP strains have been corrected and differ from the ones used in previous papers (Pelisch and Hay, 2016; Pelisch et al., 2014).

Strain	Genotype	
FGP1	*fgpIs20[pFGP79; Ppie-1 mCherry::smo-1(GG) unc-119(+)], unc-119 (ed3)*	FGP Lab
FGP2	*fgpIs21[pFGP80; Ppie-1 mCherry::smo-1(GA) unc-119(+)], unc-119 (ed3)*	FGP Lab
FGP3	*fgpIs23[pFGP78; pie-1/GFP-TEV-S-Tag::smo-1(GG) unc-119(+)], unc-119 (ed3)*	FGP Lab
FGP4	*fgpIs24[pFGP77; pie-1/GFP-TEV-S-Tag::smo-1(GA) unc-119(+)], unc-119 (ed3)*	FGP Lab
FGP8	*ruIs32 [pie-1::GFP::H2B + unc-119(+)], fgpIs20[pFGP79; Ppie-1 mCherry::smo-1(GG) unc-119(+)], unc-119 (ed3)*	FGP Lab
FGP17	*fgpIs37[pFGP60; Psmo-1::6xHis::smo-1(L88K,GG)::smo-1 3'UTR unc-119(+)], unc-119 (ed3)*	this study
FGP9	*fgpIs23[pFGP78; pie-1/GFP-TEV-S-Tag::smo-1(GG) unc-119(+)], unc-119 (ed3); ltIs37 [pAA64; pie-1p::mCherry::his-58 + unc-119(+)]*	this study
FGP10	*fgpIs24[pFGP77; pie-1/GFP-TEV-S-Tag::smo-1(GA) unc-119(+)], unc-119 (ed3); ltIs37 [pAA64; pie-1p::mCherry::his-58 + unc-119(+)]*	this study
EU630	*air-2(or207) I*	CGC
HY604	*mat-1(ye121) I*	CGC
GG48	*emb-27(g48) II*	CGC
OD987	*ltSi264[pOD1949/pTK011; Pbub-1::Bub1 reencoded::mCherry; cb-unc-119(+)]II;unc-119(ed3)III*	OD Lab
VC1915	*klp-18(ok2519) IV/nT1 [qIs51] (IV;V)*	CGC
OD987	*ltSi264[pOD1949/pTK011; Pbub-1::Bub1 reencoded::mCherry; cb-unc-119(+)]II;unc-119(ed3)III*	OD lab
FGP24	*ltSi264[pOD1949/pTK011; Pbub-1::Bub1 reencoded::mCherry; cb-unc-119(+)]II;unc-119(ed3)III; ruIs32 [pie-1::GFP::H2B + unc-119(+)], unc-119 (ed3)*	this study
FGP26	*ltSi264[pOD1949/pTK011; Pbub-1::Bub1 reencoded::mCherry; cb-unc-119(+)]II;unc-119(ed3)III; fgpIs23[pFGP78; pie-1/GFP-TEV-S-Tag::smo-1(GG) unc-119(+)], unc-119 (ed3)*	this study
FGP28	*gei-17(fgp1[GFP::FLAG::degron::loxP::gei-17])*	this study
FGP29	*gei-17(fgp1[GFP::FLAG::degron::loxP::gei-17]); ieSi38[Psun-1::TIR1::mRuby::sun-1 3'UTR,cb-unc-119(+)] IV*	this study
FGP30	*gei-17(fgp1[GFP::FLAG::degron::loxP::gei-17]); ltIs37 [pAA64; pie-1p::mCherry::his-58 + unc-119(+)]*	this study
FGP36	*klp-19(fgp3[klp-19 A2618>G, A2623>C, A2625>T, T2629>C, G2631>T, T2634>C, A2637>G, T2640>C, G2643>C, A2646>G])*	this study

Generation of GFP-FLAG-degron-GEI-17. GEI-17 fused to GFP-FLAG-degron was generated by CRISPR exactly as described (Dickinson et al., 2015), using a pDD282-based plasmid as a repair template generated by Gibson assembly (NEBuilder HiFi DNA Assembly Master Mix, New England Biolabs). We targeted the N-terminus of GEI-17 as this is the same in all the GEI-17 isoforms. Two gRNAs were used (5'-GTCGTTTCGAGACACAGCGG-3' and 5'-GAGACACAGCGGAGGATCGG-3'). Both sequences target PAM motifs upstream of the initial ATG codon, thus in order to avoid Cas9 cleavage on the repaired locus, we made a G>A mutation in the –15 position, relative to the ATG within the *gei-17* promoter region. Injections and screening were performed by Knudra Transgenics. The degron sequence consisted of the 44-aa fragment of the Arabidopsis thaliana IAA17 protein (Morawska and Ulrich, 2013; Zhang et al., 2015). Sequences of all plasmids are available upon request.

Generation of KLP-19 K873R. Point mutation in the endogenous *klp-19* locus was achieved through ALT-R CRISPR-Cas9 system (IDT), with a protocol provided by Simone Köhler and Abby Dernburg. Cas9 protein was produced in-house or purchased from PNA Bio. Cas9 (30 μM) was incubated for 5 min with a 1:1 mixture of crRNA:tracrRNA (30 μM). The injection mix also contained the repair template (100 ng/μl), and marker plasmids pCFJ90 (2.5 ng/μl) and pCFJ104 (5 ng/μl). gRNA sequence was 5'-CAGAGAGUUGGCUCAAUGUC-3', and the repair template sequence (ssDNA, Ultramer oligo, IDT) was the following:

```
aagttggcgcgaacaaagtccgagttcacggcgaaaattgcttcgaaagccagccacgaggagaagagaaagaaggagga
tgaggagatgagagcaaGatacCgTgagCtTgcCcaGtgCctCgaGgatgccaagtctggattgcatgaaaagatcgctt
tcctcctgtgcttgatcaaggaaaatcgggtc
```

G: point mutation to generate an Arg in position 873.

X: Synonymous mutations to avoid Cas9 cleavage of repaired sequence.

X: Synonymous mutations to generate an Xho I site for screening (in Leu 881 and Asp 882).

Primers klp-19screenfwd: 5'-gagacgtatatctgcgag-3' and klp-19screenrev: 5'-cctcccatgaagtttgtc-3' were used for screening and sequencing.

Auxin treatment. Auxin (IAA, Sigma-Aldrich I5148) was used at 1 mM final concentration in standard NGM plates, unless otherwise noted. All plates for auxin treatment were prepared, allowed to dry for 2 days and a lawn of concentrated OP50 bacteria was seeded, as auxin inhibits bacterial growth. For auxin treatment, worms were placed on auxin-containing plates for the indicated time.

RNAi. Bacterial (HT115) clones expressing dsRNA for feeding strains were obtained from a commercial library (Kamath and Ahringer, 2003). Bacteria were grown at 37°C to OD_{600} = 1 and then spread on nematode growth media plates supplemented with 1 mM IPTG and incubated for 24 h at 20°C. For most experiments, L4 worms were then added to plates and fed for 24–32 h at 20°C, before analysis. For experiments involving APC alleles, worms were shifted to 25°C for 5-7 h before fixing or imaging. For experiments with monopolar spindles, L1 *klp-18(ok2519)* worms were fed with bacteria expressing *emb-30(RNAi)* for 3 days at 15°C and then changed to plates containing *emb-30(RNAi)* plus control or SUMO pathway RNAi (1:1 mix) for a further 2 days at 15°C.

Peptide injection. The experiment in Figure 7J was performed by injecting either a SIM consensus peptide (VDVIDLTIEEDE) (Bruderer et al., 2011) or a control peptide (YGSFQDSVSMREDC), both at 700 μM, into the gonads of *emb-27* young adults. After 24 recovery at 15°C, worms were shifted to 25°C for 5 h to induce metaphase I arrest. Then, worms were processed for embryo immunofluorescence using FITC-labelled anti-α-tubulin (DM1A, Abcam, ab64503), Alexa 594-labelled anti-KLP-19, and Alexa 647-labelled anti-BUB-1 antibodies.

Antibodies. Antibodies against SMO-1, GEI-17, and UBC-9 were reported previously (Pelisch et al., 2014). An anti-SMO-1 monoclonal antibody is available from the Developmental Studies Hybridoma Bank (DSHB, http://dshb.biology.uiowa.edu/SUMO-6F2). The All sera were adsorbed with HT115 bacterial lysate and affinity purified with the antigenic peptide/protein (except for KLP-19, see below). AIR-2 (CQKIEKEASLRNH) and ICP-1 (VKVKKRGSSAVWK) peptide antibodies were produced and affinity purified by Moravian Biotech using previously described peptides (Burrows et al., 2006; Schumacher et al., 1998). Anti-KLP-19 serum (Powers et al., 2004) was subject to protein A purification before use. The resulting antibody was used at 2 μg/ml. A monoclonal antibody recognising alpha-tubulin was used at 2.5 μg/ml (clone DM1A, Sigma). Anti-BUB-1 (Oegema et al., 2001) and anti SEP-1 (Bembenek et al., 2007) antibodies were used at 0.5 μg/ml. A complete list of the antibodies used in the present study is included below:

antibody	species	WB	IF
UBC-9 (full length)	Sheep	1 μg/ml	10 μg/ml
GEI-17 (aa 133-509)	Rabbit	1 μg/ml	10 μg/ml
ICP-1 (aa 591-604)	Rabbit	N/A	1 μg/ml
AIR-2 (aa 294-305)	Rabbit	1 μg/ml	1 μg/ml
pH3 (Merck-Millipore, #06-570)	Rabbit	N/A	0.2 μg/ml
MPM-2 (Thermo 0.T.181)	Mouse	N/A	2 μg/ml
NDC-80 (Novus Biologicals, #42000002)	Rabbit	N/A	1 μg/ml
SMO-1 (full length)	Mouse	1 μg/ml	1 μg/ml
SMO-1 (full length)	Sheep	1 μg/ml	1 μg/ml
Tubulin (DM1A, Sigma-Aldrich #T6199)	Mouse	N/A	2.5 μg/ml
Tubulin (DM1A, FITC, Abcam #ab64503)	Mouse		
SYP-1	Rabbit	N/A	1/100
REC-8	Mouse	N/A	1/100
HTP-3 (Monique Zetka)	Rabbit	N/A	1/100
HCP-6 (Barbara Meyer)	Rabbit	N/A	5 μg/ml
KLP-19 (Susan Stromme)	Rabbit	1 μg/ml	2 μg/ml
BUB-1 (Tony Hyman)	Rabbit	N/A	0.5 μg/ml
SEP-1 (Andy Golden)	Rabbit	N/A	0.5 μg/ml

Primary antibody labelling. For all experiments involving fluorescence intensity measurements, antibodies were labelled with Alexa fluorophores. The APEX Alexa Fluor labelling kits (Thermo Scientific) were used and antibodies

were labelled with Alexa 488, Alexa 594, and Alexa 647, following the manufacturer's indications. Antibodies were buffer exchanged to PBS using Zeba™ Spin Desalting Columns (Thermo Scientific) and were stored in small aliquots at -20°C in PBS containing 10% glycerol. Labelled antibodies were used at 1-5 μg/ml for immunofluorescence.

In utero embryo live imaging. For live imaging, GFP- or mCherry-expressing worms were picked into a solution of tricaine (0.1%) and tetramisole (0.01%), and incubated for 20 min. Worms were then pipetted onto a 4% agar pad, covered with a coverslip, and imaged with a spinning-disk confocal microscope (MAG Biosystems) mounted on a microscope (IX81; Olympus) with a 100×/1.45 Plan Apochromat oil immersion lens (Olympus), a camera (Cascade II; Photometrics), spinning-disk head (CSU-X1; Yokogawa Electric Corporation), and MetaMorph software (Molecular Devices). Image stacks were obtained at 1.5 μm z-steps and 15- or 30-s intervals using 2x2 binning.

Immunostaining. Worms were placed on 4 μl of M9 worm buffer in a poly-D-lysine (Sigma, P1024)-coated slide and a 24x24-cm coverslip was gently laid on top. Once the worms extruded the embryos, slides were placed on a metal block on dry ice for >10 min. The coverslip was then flicked off with a scalpel blade, and the samples were fixed in methanol at −20°C for 30 min (except for GFP, where the methanol treatment lasted for 5 min). Embryos were stained using standard procedures. Secondary antibodies were anti–sheep, anti-mouse, or anti-rabbit conjugated to Alexa Fluor 488, Alexa Fluor 594, and Alexa Fluor 647 (1:1,000, Thermo Scientific). DNA was visualized with Hoechst 33258 (Thermo Scientific, 1.5 μg/ml final concentration in PBS, 0.05% Tween-20) or DAPI (Sigma Aldrich, 1 μg/ml final concentration in PBS, 0.05% Tween-20). Embryos were mounted in ProLong Diamond antifade mountant (Thermo Scientific).

Plasmids. *C. elegans smo-1*, *ubc-9, gei-17, and air-2* cDNAs were cloned in the pHISTEV30a vector that includes an N-terminal hexahistidine tag followed by a TEV protease recognition site (Pelisch and Hay, 2016; Pelisch et al., 2014). Full length KLP-19 and fragments were codon-optimised for expression in *E. Coli* (Genscript) and sub-cloned in the pLou3 vector containing a 6xHis-MBP tag, followed by a TEV protease site. BUB-1(1-689) was codon-optimised for expression in *E. Coli* (Genscript) and sub-cloned in the pHISTEV30a vector containing a 6xHis tag, followed by a TEV protease site. GEI-17(423-602) and BUB-1(2-551) were cloned into pLou3 by Gibson Assembly (NEBuilder HiFi, NEB). All constructs were verified by sequencing.

SMO-1, UBC-9, and GEI-17 purification. SMO-1, UBC-9, and GEI-17 were tagged in their N-terminus with a 6xHis tag followed by a TEV protease cleavage site and expressed and purified essentially as described (Pelisch and Hay, 2016; Pelisch et al., 2014). The basic protocol consisted in an initial purification over a Ni-NTA column (QIAGEN), followed by 6xHis-TEV protease treatment. Any uncleaved material, as well as the 6xHis tag, and the 6xHis-TEV protease were removed with Ni-NTA beads, and the resulting untagged proteins were further purified by size exclusion chromatography using a HiLoad 16/600 Superdex 75 pg column (for SMO-1 and UBC-9, GE Healthcare) or a HiLoad 16/600 Superdex 200 pg column (for GEI-17, GE Healthcare). Lysis was performed in 50 mM Tris, 500 mM NaCl, 10 mM imidazole, 0.5 mM TCEP, pH 7.5 (with protease inhibitors, Roche). Subsequent purification steps were performed with 50 mM Tris, 150 mM NaCl, 0.5 mM TCEP, pH 7.5, and varying imidazole concentrations.

KLP-19 purification. KLP-19 and fragments were expressed as N-terminal fusions to 6xHis-MBP, followed by a TEV protease cleavage site. Bacterial cultures were grown until OD_{600} = 0.8, cooled on ice, and induced with 0.1 mM IPTG for 16 hs at 20°C. The bacterial cells were harvested by centrifugation (5,000 g for 20 min at 4ºC) and the cell pellet was resuspended in 50 ml of lysis buffer per L of culture [50 mM Tris, 300 mM KCl, 2 mM $MgSO_4$, 0.5 mM EGTA, 0.5 mM TCEP, and Complete protease inhibitor cocktail tablet, EDTA-free (Roche), pH 7.5]. Bacteria were lysed by sonication (Digital Sonifier, Branson) for 8 X 20" pulses at 50% amplitude, with a 30-second cooling period between pulses. Samples were centrifuged (27,200 g for 45 min at 4ºC) to remove any insoluble material and the supernatant was loaded onto an amylose column (NEB) pre-equilibrated with lysis buffer. The column was washed successively with lysis buffer (~10 column volumes) and the fusion protein was then eluted with lysis buffer supplemented with 10 mM maltose. TEV protease was added (1 mg of TEV protease per 30 mg of the fusion protein) and incubated ~16 hours at 4°C. Samples were centrifuged (3,900 g for 15 min at 4°C) to remove any precipitated material and buffer was exchanged to 80 mM PIPES-KOH, 50 mM KCl, 2 mM $MgSO_4$, 0.5 mM EGTA, 0.5 mM TCEP, pH 6.9 using Zeba spin columns (Thermo Scientific). For full-length KLP-19, the protein was concentrated and purified over a Superose 6 prep grade XK 16/70 column (GE Healthcare). For the 651-1083 fragment, a Ni-NTA column was used to remove the 6xHis-TEV protease, the 6xHis-MBP tag, and any remaining uncleaved 6xHis-MBP-tagged KLP. KLP-19(651-1083) was further purified by size exclusion chromatography with a HiLoad 16/600 Superdex 200 pg column. Purified proteins were aliquoted, flash-frozen in liquid nitrogen, and stored at -80°C.

Pull-down experiments. For the pulls-down in Figures 7H and 7H, MBP and MBP fusion proteins were expressed in bacteria, bound to amylose beads and washed extensively before pull-down experiments. SUMO-modified GEI-17(133-509) (~2 μM) or KLP-19(651-1083) (~5 μM) were incubated for ~5 minutes on ice with MBP or MBP-fusion proteins as indicated (5 μM) and 30 μl of amylose beads in total volume of 70 μl. Samples were buffered in 50 mM Tris, 150 mM NaCl, 0.5 mM TCEP, 5% (v/v) glycerol, pH 7.5 (binding buffer). Subsequently, amylose beads were collected on

the bottom of the tube by centrifugation and the supernatant was aspired. Beads were resuspended in 0.5 ml of binding buffer and overlaid on 1.4 ml of 50 mM Tris, 150 mM NaCl, 0.5 mM TCEP, 5% (v/v) glycerol, 10% (w/v) sucrose, pH 7.5 in a new tube. Beads were then collected by centrifugation, the supernatant was aspired and the washing step was repeated. All washing procedures were carried out as quickly as possible using pre-chilled buffers. Bound material was eluted from the beads by addition of SDS-PAGE loading buffer and analyzed by Western blotting.

In vitro sumoylation. Conjugation assays contained 50 mM Tris-HCl, 5 mM dithiothreitol, 5 mM $MgCl_2$, 2 mM ATP, 20 μM SUMO, 100 nM of SAE1/SAE2, 140 nM UBC-9 (for GEI-17-dependent reactions), and 3.3 μM UBC-9 (for GEI-17-independent reactions). KLP-19 was used at 1 μM, KLP-19(651-1083) at 6 μM and GEI-17 ranged from 12.5 to 50 nM, as indicated. Reactions were incubated at 37°C for the indicated times. Reactions were analysed by coomassie staining, regular western blotting, or dual-color western blotting using an Odyssey CLx (LI-COR).

Duolink® *in situ* Proximity Ligation Assay (PLA®). Proximity ligation assays were performed using primary antibodies directly coupled to the PLA probes or using secondary antibody PLA probes (Sigma-Aldrich). For the direct PLA, ~35 worms were placed on a drop of 4 μl of M9 worm buffer in a poly-D-lysine-coated slide and a coverslip was gently laid on top. Once the worms extruded the embryos, slides were freeze-cracked: placed on a metal block on dry ice for >10 min., the coverslip taken off with a scalpel blade, and samples fixed in methanol at −20°C for 30 min. After sequential washes (5 min each) with PBS + 0.5% Triton X-100, PBS + 0.1% Tween-20, and PBS, slides were incubated with the monoclonal α-SMO-1 (6F2/D1, 10 μg/ml) and α-AIR-2, α-BUB-1, or α-KLP-19 (all at 10 μg/ml), previously coupled to the PLA oligonucleotide arms using the Duolink® in situ Probemaker overnight at 4°C. Ligation and amplification were performed as detailed by the manufacturer. Controls omitting either of the antibodies gave no PLA signal. For indirect PLA, the same primary antibodies were used (unlabeled) and after an overnight incubation at 4°C, slides were incubated with anti-mouse and anti-rabbit secondary antibodies coupled to the PLA oligonucleotide probes. Ligation and amplification were performed as detailed by the manufacturer. In both cases, slides were incubated in Hoechst 33258 at 1.5 μg/ml in PBS + 0.1% Tween-20 for 5 min. Slides were mounted in 4% n-propyl-gallate, 90% glycerol, in PBS and were imaged using a DeltaVision Elite microscope.

In vivo identification of SUMO conjugation sites. Worms expressing His_6-SUMO(L88K) were grown in liquid culture with S-medium (50 mM potassium phosphate, 100 mM NaCl, 10 mM potassium citrate, 3 mM $CaCl_2$, 3 mM $MgCl_2$, 5 µg/ml cholesterol, and trace metals, pH 6) supplemented with OP50 bacteria. Five different sets of synchronised liquid cultures were lysed 24 h after >75% of the population was at the L4 stage. Twenty five grams of worm pellet were lysed with 125 ml of lysis buffer (6 M Gu-HCl, 100 mM Na_2HPO_4/NaH_2PO_4, 10 mM Tris-HCl, 10 mM imidazole, 5 mM β-mercaptoethanol, pH 8). The sample was sonicated with 6 cycles of 20 sec on and 20 sec off in ice using a tip sonicator at 55% amplitude. After centrifugation at 45,000 g for 30 min at 4°C, the supernatant was passed through a 0.2 µm filter and protein concentration was measured using the BCA assay, with BSA as a standard. Protein concentration was 10 mg/ml in a total volume of 150 ml. Subsequently, 4 ml of packed Ni-NTA beads pre-equilibrated in lysis buffer were added and incubated overnight at 4°C in 4 50-ml tubes. At this stage, 100 µl of the mix were taken, beads were pelleted, and the bound and flow-through fractions were run on SDS-PAGE to perform anti-SUMO western blotting. For purification of the His_6-sumoylated proteins, the content of the 4 tubes were loaded on a column, and the Ni-NTA beads were washed with 5 column volumes (CVs) of lysis buffer, 10 CVs of Urea Buffer I (8 M Urea, 100 mM Na_2HPO_4/NaH_2PO_4, 10 mM Tris-HCl, 10 mM imidazole, 5 mM β-mercaptoethanol, pH 8), 10 CVs of Urea Buffer II (8 M Urea, 100 mM Na_2HPO_4/NaH_2PO_4, 10 mM Tris-HCl, 10 mM imidazole, 5 mM β-mercaptoethanol, pH 6.3), 5 CVs of Urea Buffer I, and eluted with 3 CVs of elution buffer (8 M Urea, 100 mM Na_2HPO_4/NaH_2PO_4, 10 mM Tris-HCl, 200 mM imidazole, 5 mM β-mercaptoethanol, pH 8). We obtained approximately 4 mg of protein, as judged by quantitation using the BCA method.

Proteins were concentrated on Vivacon 2 30-kDa-filter units at 3,000 g (20°C) until the filters were dry. The filter was washed twice with 3 ml of UA Buffer (8 M urea, 100 mM Tris-HCl pH 8.0). Then, 1.5 ml of 50 mM chloroacetamide (CAA) in UA buffer were added and incubated for 20 min in the dark. The filter was centrifuged at 3,000 g until dry, washed with 3 ml of UA buffer, and then twice with 3 ml of IP buffer (50 mM MOPS pH 7.2, 10 mM Na_2HPO_4, 50 mM NaCl). Lys-C was added at an enzyme to protein ratio of 1:50 in a final volume of 10 CVs IP buffer. Incubation proceeded at 37°C (in a wet chamber) for ~16 h. Filters were then centrifuged at 3,000 g until dry and washed twice with 270 µl of IP buffer. Peptide samples were incubated at 95 °C for 5 min to inactivate residual lys-C activity. Peptides were stored at -80°C for further processing. Then, Glu-C was added to the filters at an enzyme to protein ratio of 1:100 in IP buffer. Filter units were incubated at room temperature (in a wet chamber) for ~16 h and centrifuged at 3000 g until the filter was dry. Filters were washed twice with 270 µl of IP buffer and the peptides were then incubated at 95 °C for 5 min to inactivate residual Glu-C activity. Peptides were stored at -80°C.

For IP, 19 µg of BS^3 crosslinked anti-K-ε-GG antibody were added and incubated ON (~24 h) at 4 °C while rotating. Samples were centrifuged at 1,000 g for 1 min at 4°C and beads were allowed to settle for 5 min on ice. Supernatant was transfered to another tube, leaving approximately 100 µl of supernatant fraction on top of the beads. Beads were resuspended and transferred into an 0.5 ml eppendorf tube. The bead-supernatant slurry was centrifuged at 1,000 g for 1

min at 4°C and beads were allowed to settle for 5 min on ice. The rest of the supernatant fraction was transferred to the same flow-through tube used in the last step of the protocol. Flow-through fraction was stored at -80 °C. Beads were washed twice by adding 150 µl of cold IP buffer, centrifugation at 1,000 g for 1 min 4 °C and allowing the beads settle for 5 min on ice. Solutions were stored at -80 °C. K-ε-GG peptides were eluted twice with 50 µl of 0.15% TFA. Peptide solution was separated from beads and beads were stored in 3 µl of IP buffer at 4 °C (50% bead solution). Peptides were desalted on 3x C18 stagetips and eluted with K-ε-GG buffer B (80% acetonitrile, 0.1% TFA). Peptides were concentrated in a speed-vac and resuspended in 0.1% TFA.

Liquid chromatography tandem mass spectrometry (LC-MS/MS). Each desalted sample of peptides was analysed twice using EASY-nLC 1000 nano-flow UHPLC system, EASY-Spray ion source and Q Exactive hybrid quadrupole-Orbitrap mass spectrometer (all Thermo Scientific). Peptides were loaded onto 2 cm Acclaim PepMap 100 C18 nanoViper pre-column (75 µm inner diameter; 3 µm particles; 100 Å pore size) at a constant pressure of 800 bar and separated using 50 cm EASY-Spray PepMap RSLC C18 analytical column (75 µm inner diameter; 2 µm particles; 100 Å pore size) maintained at 45°C. Exploratory analysis was performed with 10% of the sample and peptides were separated during either a 35 or 60 min linear gradient of 5−25% or 5-22% (vol/vol) of acetonitrile in 0.1% (vol/vol) of formic acid, respectively, at a flow rate of 250 nL/min, followed by a 10 or 12 min linear increase of acetonitrile to 50 or 40% (vol/vol), respectively. Total length of the gradient including column washout and re-equilibration was 60 or 90 min, respectively. Comprehensive peptide analysis was performed using a 60 min linear gradient of 5−22% acetonitrile in 0.1% formic acid at a flow rate of 250 nL/min, with a subsequent 12 min linear increase of acetonitrile to 40%. The overall length of the gradient during comprehensive analysis was 90 min.

Peptides eluting from the LC column were charged using electrospray ionization and MS data was acquired online in a profile spectrum data format. Full MS scan covered a mass range of mass-to-charge ratio (m/z) 300−1800 or 300−1600 during exploratory or comprehensive peptide analysis, respectively. Target value was set to 1 000 000 ions with a maximum injection time (IT) of 20 ms and full MS was acquired at a mass resolution of 70 000 at m/z 200. Data dependent MS/MS scan was initiated if the intensity of a mass peak reached a minimum of 20 000 ions. During exploratory LC-MS/MS analysis, up to 10 most abundant ions were selected using 2 Th mass isolation range when centered at the parent ion of interest. For comprehensive analyses, the most abundant ion was exclusively picked for MS/MS. Selection of molecules with peptide-like isotopic distribution was preferred. Target value for MS/MS scan was set to 500 000 with a maximum IT of 60 ms and resolution of 17 500 at m/z 200 for exploratory, or maximum IT of 1000 ms and a resolution 35 000 at m/z 200 for comprehensive peptide analyses. Precursor ions were fragmented by higher energy collisional dissociation (HCD) using a normalised collision energy of 30 and fixed first mass was set to m/z 100. Precursor ions with undetermined, single, or high (>8) charge state were rejected. Ions triggering a data-dependent MS/MS scan were placed on the dynamic exclusion list for 40 s (exploratory analyses) or 60 s (comprehensive analyses) and isotope exclusion was enabled.

Analysis of MS data. Raw MS files were analysed using MaxQuant software package (version 1.3.0.5) (Cox and Mann, 2008) and peak lists were searched with an integrated Andromeda search engine (Cox et al., 2011) against an entire *C. elegans* UniProtKB proteome (Apweiler et al., 2004) including canonical and isoform sequences downloaded in a FASTA format in May 2014 or May 2016 and supplemented with the sequence of *C. elegans* SUMO(L88K) and the following *H. sapiens* proteins: UBC9 (P63279), SAE1 (Q9UBE0) and SAE2 (Q9UBT2). Raw files were divided into two parameter groups based on the specificity of proteolysis applied during sample preparation. Hydrolysis of peptide bonds C-terminal to Lys residues with a maximum of three missed cleavages was allowed for peptides processed exclusively with Lys-C. Samples acquired after an additional Glu-C digestion were analysed with enzyme specificity set to C-terminal to Lys, Glu or Asp with a maximum of five missed cleavages. Carbamidomethylation of cysteine residues was specified as a fixed modification and oxidation of methionines, acetylation of protein N-termini, and Gly-Gly adduct on internal lysine residues were selected as variable modifications. Maximum peptide mass of 10 000 Da was allowed, multiplicity was set to 1 and re-quantify option was disabled. Decoy sequence database was generated using Lys as a special amino acid. Default values were chosen for the rest of the parameters.

The mass spectrometry proteomics data have been deposited to the ProteomeXchange Consortium via the PRIDE (Vizcaino et al., 2016) partner repository with the dataset identifier PXD005202.

Statistical analysis. Bivalent-to-pole distances in monopolar spindles (Figure 1H) and non-RC KLP-19 localisation (Figure 3K) were analysed with the Kruskal-Wallis test, followed by Dunn's post-test. In Figures 3H and S5C, fluorescence intensity (corrected for background fluorescence) were measured in ImageJ and then analysed using Mann-Whitney tests. All tests were performed with GraphPad Prism version 6.00 for Mac OS X (GraphPad Software, La Jolla California USA).

Supplemental References

Apweiler, R., Bairoch, A., Wu, C.H., Barker, W.C., Boeckmann, B., Ferro, S., Gasteiger, E., Huang, H., Lopez, R., Magrane, M.*, et al.* (2004). UniProt: the Universal Protein knowledgebase. Nucleic Acids Res 32, D115-119.

Bembenek, J.N., Richie, C.T., Squirrell, J.M., Campbell, J.M., Eliceiri, K.W., Poteryaev, D., Spang, A., Golden, A., and White, J.G. (2007). Cortical granule exocytosis in C. elegans is regulated by cell cycle components including separase. Development 134, 3837-3848.

Brenner, S. (1974). The genetics of Caenorhabditis elegans. Genetics 77, 71-94.

Bruderer, R., Tatham, M.H., Plechanovova, A., Matic, I., Garg, A.K., and Hay, R.T. (2011). Purification and identification of endogenous polySUMO conjugates. EMBO Rep 12, 142-148.

Burrows, A.E., Sceurman, B.K., Kosinski, M.E., Richie, C.T., Sadler, P.L., Schumacher, J.M., and Golden, A. (2006). The C. elegans Myt1 ortholog is required for the proper timing of oocyte maturation. Development 133, 697-709.

Cheeseman, I.M., and Desai, A. (2005). A combined approach for the localization and tandem affinity purification of protein complexes from metazoans. Science's STKE : signal transduction knowledge environment 2005, pl1.

Cox, J., and Mann, M. (2008). MaxQuant enables high peptide identification rates, individualized p.p.b.-range mass accuracies and proteome-wide protein quantification. Nat Biotechnol 26, 1367-1372.

Cox, J., Neuhauser, N., Michalski, A., Scheltema, R.A., Olsen, J.V., and Mann, M. (2011). Andromeda: a peptide search engine integrated into the MaxQuant environment. J Proteome Res 10, 1794-1805.

Dickinson, D.J., Pani, A.M., Heppert, J.K., Higgins, C.D., and Goldstein, B. (2015). Streamlined Genome Engineering with a Self-Excising Drug Selection Cassette. Genetics 200, 1035-1049.

Kamath, R.S., and Ahringer, J. (2003). Genome-wide RNAi screening in Caenorhabditis elegans. Methods 30, 313-321.

Morawska, M., and Ulrich, H.D. (2013). An expanded tool kit for the auxin-inducible degron system in budding yeast. Yeast 30, 341-351.

Oegema, K., Desai, A., Rybina, S., Kirkham, M., and Hyman, A.A. (2001). Functional analysis of kinetochore assembly in Caenorhabditis elegans. J Cell Biol 153, 1209-1226.

Pelisch, F., and Hay, R.T. (2016). Tools to Study SUMO Conjugation in Caenorhabditis elegans. In SUMO: Methods and Protocols, S.M. Rodriguez, ed. (New York, NY: Springer New York), pp. 233-256.

Pelisch, F., Sonneville, R., Pourkarimi, E., Agostinho, A., Blow, J.J., Gartner, A., and Hay, R.T. (2014). Dynamic SUMO modification regulates mitotic chromosome assembly and cell cycle progression in Caenorhabditis elegans. Nat Commun 5, 5485.

Powers, J., Rose, D.J., Saunders, A., Dunkelbarger, S., Strome, S., and Saxton, W.M. (2004). Loss of KLP-19 polar ejection force causes misorientation and missegregation of holocentric chromosomes. J Cell Biol 166, 991-1001.

Praitis, V., Casey, E., Collar, D., and Austin, J. (2001). Creation of low-copy integrated transgenic lines in Caenorhabditis elegans. Genetics 157, 1217-1226.

Schumacher, J.M., Golden, A., and Donovan, P.J. (1998). AIR-2: An Aurora/Ipl1-related protein kinase associated with chromosomes and midbody microtubules is required for polar body extrusion and cytokinesis in Caenorhabditis elegans embryos. J Cell Biol 143, 1635-1646.

Vizcaino, J.A., Csordas, A., Del-Toro, N., Dianes, J.A., Griss, J., Lavidas, I., Mayer, G., Perez-Riverol, Y., Reisinger, F., Ternent, T., *et al.* (2016). 2016 update of the PRIDE database and its related tools. Nucleic Acids Res.

Wallenfang, M.R., and Seydoux, G. (2000). Polarization of the anterior-posterior axis of C. elegans is a microtubule-directed process. Nature 408, 89-92.

Zhang, L., Ward, J.D., Cheng, Z., and Dernburg, A.F. (2015). The auxin-inducible degradation (AID) system enables versatile conditional protein depletion in C. elegans. Development 142, 4374-4384.
