# Supplementary material for: A SUMO-Dependent Protein Network Regulates Chromosome Congression during Oocyte Meiosis
Source: Mol Cell. 2017 Jan 5;65(1):66–77. doi: 10.1016/j.molcel.2016.11.001 (PMC5222697; doi:10.1016/j.molcel.2016.11.001)
Supplement: Document S2. Article plus Supplemental Information [file mmc2.pdf]

Molecular Cell

Article

A SUMO-Dependent Protein Network Regulates Chromosome Congression during Oocyte Meiosis

Graphical Abstract

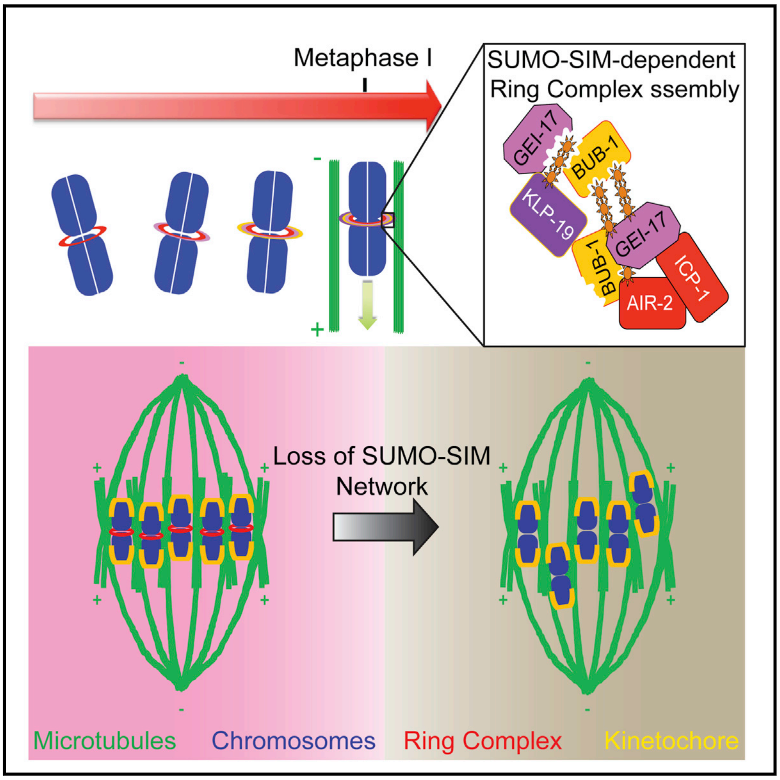

Highlights

- SUMO conjugates accumulate in the ring complex within the midbivalent during meiosis
- Ring complex assembly requires active sumoylation
- The SUMO E3 ligase GEI-17/PIAS sumoylates the chromokinesin KLP-19 in vivo and in vitro
- Meiotic chromosome congression depends on sumoylation and non-covalent SUMO binding

Authors

Federico Pelisch, Triin Tammsalu, Bin Wang, Ellis G. Jaffray, Anton Gartner, Ronald T. Hay

Correspondence

r.t.hay@dundee.ac.uk

In Brief

Pelisch et al. provide evidence that highly dynamic, coordinated, and spatially constrained sumoylation regulates chromosome congression during meiosis in *C. elegans* oocytes.

Pelisch et al., 2017, Molecular Cell *65*, 66–77
January 5, 2017 © 2017 The Author(s). Published by Elsevier Inc.
http://dx.doi.org/10.1016/j.molcel.2016.11.001

A SUMO-Dependent Protein Network Regulates Chromosome Congression during Oocyte Meiosis

Federico Pelisch,[1] Triin Tammsalu,[1] Bin Wang,[1] Ellis G. Jaffray,[1] Anton Gartner,[1] and Ronald T. Hay[1,2,*]
[1]Centre for Gene Regulation and Expression, Sir James Black Centre, School of Life Sciences, University of Dundee, Dundee, DD1 5EH, UK
[2]Lead Contact
*Correspondence: r.t.hay@dundee.ac.uk
http://dx.doi.org/10.1016/j.molcel.2016.11.001

SUMMARY

During *Caenorhabditis elegans* oocyte meiosis, a multi-protein ring complex (RC) localized between homologous chromosomes, promotes chromosome congression through the action of the chromokinesin KLP-19. While some RC components are known, the mechanism of RC assembly has remained obscure. We show that SUMO E3 ligase GEI-17/PIAS is required for KLP-19 recruitment to the RC, and proteomic analysis identified KLP-19 as a SUMO substrate in vivo. In vitro analysis revealed that KLP-19 is efficiently sumoylated in a GEI-17-dependent manner, while GEI-17 undergoes extensive auto-sumoylation. GEI-17 and another RC component, the kinase BUB-1, contain functional SUMO interaction motifs (SIMs), allowing them to recruit SUMO modified proteins, including KLP-19, into the RC. Thus, dynamic SUMO modification and the presence of SIMs in RC components generate a SUMO-SIM network that facilitates assembly of the RC. Our results highlight the importance of SUMO-SIM networks in regulating the assembly of dynamic protein complexes.

INTRODUCTION

Meiosis is a specialized division in which a single round of DNA replication is followed by two consecutive segregation steps. Homologous chromosomes segregate in Meiosis I, while sister chromatids segregate in Meiosis II, giving rise to haploid gametes (Duro and Marston, 2015). In contrast to mitotic spindles, meiotic spindles in many animal species (including humans and nematodes) lack centrosomes (Dumont and Desai, 2012), and how these spindles are organized is poorly understood (Ohkura, 2015). As meiotic spindles vary across the animal kingdom, identification of common and unique mechanisms of spindle assembly and chromosome orientation, congression, and segregation will contribute to the fundamental understanding of these processes (Dumont and Desai, 2012; Severson et al., 2016). In *C. elegans* oocytes, chromosome movement along lateral microtubule bundles is facilitated by plus-end directed forces exerted by the kinesin KLP-19 (Powers et al., 2004; Wignall and Villeneuve, 2009), which requires the kinase BUB-1 for localization to the ring complex (RC). BUB-1, in turn, requires the chromosomal passenger complex (CPC) components AIR-2/Aurora B and ICP-1/INCENP for RC localization (Dumont et al., 2010; Wignall and Villeneuve, 2009). However, the mechanism of RC assembly has remained elusive.

The small ubiquitin-related modifier (SUMO) conjugation pathway is conserved in *C. elegans*, and it is composed of one SUMO ortholog, SMO-1 (hereafter, SUMO), an E1 activating enzyme, and the E2 conjugating enzyme (UBC-9). The specificity and dynamic nature of this modification is achieved by SUMO-specific E3 ligases and SUMO-specific isopeptidases (Flotho and Melchior, 2013; Gareau and Lima, 2010; Hay, 2007). In *C. elegans*, these include the Siz/PIAS-type SUMO E3 ligase GEI-17 (Kim and Michael, 2008; Pelisch et al., 2014) and the isopeptidases ULP-1, 2, 4, and 5 (Pelisch et al., 2014; Sapir et al., 2014; Tsur et al., 2015; Zhang et al., 2004). SUMO E3 ligases often target groups of functionally related proteins (Psakhye and Jentsch, 2012) and, once this modification has taken place, the presence of SIMs in PIAS type E3 ligases has the potential to prolong engagement on substrate(s) leading to a dramatic amplification of the signal. Thus, SUMO-SIM networks provide a means to rapidly and reversibly regulate protein interactions.

While sumoylation regulates chromosome synapsis during meiosis in budding yeast (Cahoon and Hawley, 2016), it is not known whether this is conserved in other organisms. We addressed this using the nematode *C. elegans,* as it provides an excellent model to study meiosis (Hillers et al., 2015). In nematodes, synaptonemal complex (SC) assembly is unperturbed in the absence of SUMO, while bivalent differentiation and SC disassembly are affected (Bhalla et al., 2008). Here, we show that highly dynamic, GEI-17-mediated SUMO conjugation during fertilization and non-covalent SUMO binding facilitates the assembly of a complex containing AIR-2/Aurora B, BUB-1, and the chromokinesin KLP-19. We identified GEI-17/PIAS as the key SUMO E3 ligase required for this complex to assemble and show that it is directly involved in SUMO modification of KLP-19. SIMs present in BUB-1 and GEI-17 allow them to bind SUMO-modified forms of KLP-19, providing a mechanism for SUMO-dependent RC assembly. These results highlight the requirement for spatially and temporally regulated SUMO modification during *C. elegans* oocyte meiosis and illustrate how post-translational modifications can regulate chromosome congression on acentrosomal spindles.

 © 2017 The Author(s). Published by Elsevier Inc.
This is an open access article under the CC BY license (http://creativecommons.org/licenses/by/4.0/).

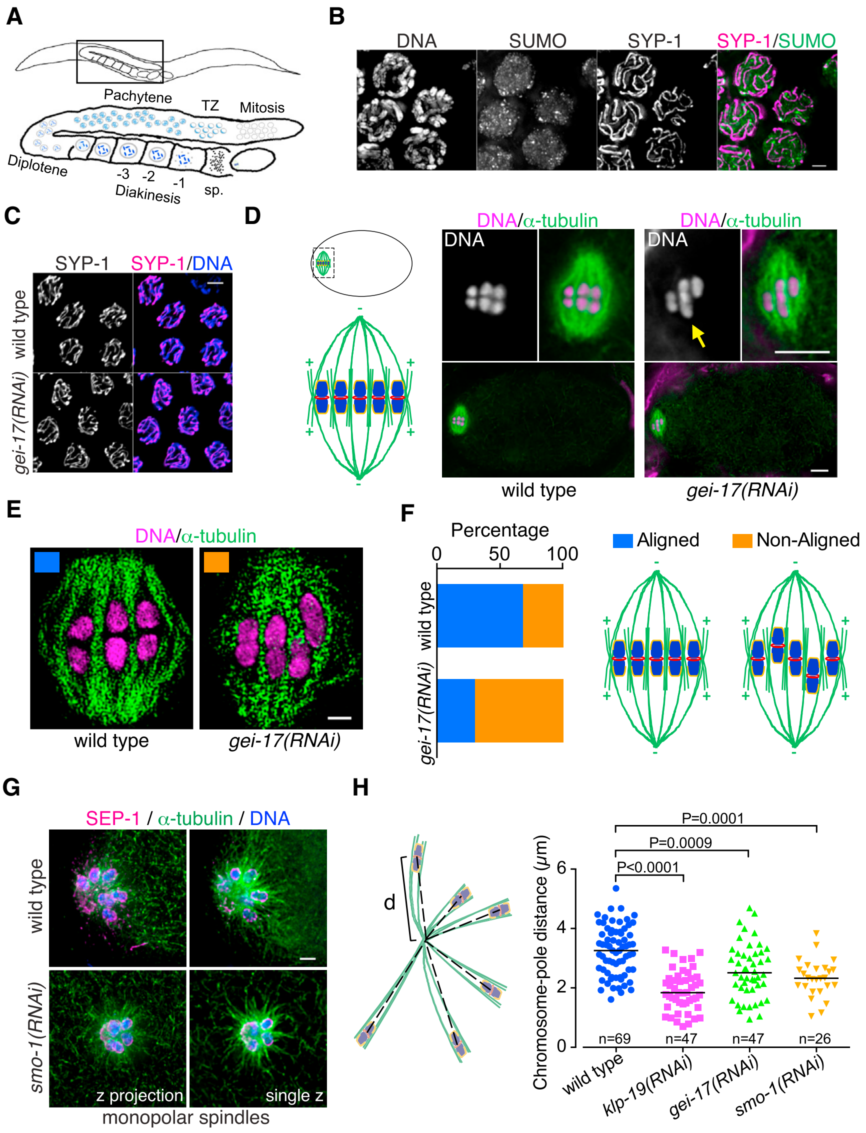

Figure 1. Sumoylation Is Required for Meiotic Chromosome Congression

(A) Schematic of a worm highlighting the germline and the different stages of prophase. transition zone, TZ; spermatheca, sp.

(B) Nuclei in the pachytene stage of meiosis showing SUMO localization in green and the synaptonemal complex component SYP-1 in magenta. The scale bar represents 2 μm. See also Figure S1B.

(C) SYP-1 staining in pachytene nuclei from wild-type and *gei-17(RNAi)* worms. The scale bar represents 3 μm. See also Figure S1E.

(D) The schematic shows a fertilized oocyte (top left) and highlights its anterior end and the meiotic spindle (bottom left). The metaphase I-arrested oocytes from *mat-1* wild-type or *gei-17(RNAi)* worms were stained for α-tubulin (green) and DNA (magenta) and widefield images are displayed. The yellow arrow points to a misaligned bivalent. The scale bar represents 5 μm. The bottom images display a view of the entire oocyte for each condition.

(E) Same as in (F), but 3D-structured illumination (SIM) images were acquired using an OMX microscope. Note that the wild-type spindle in (E) is the same as the wild-type spindle in (D). The scale bar represents 1 μm.

(F) Spindles were characterized as either "aligned" or "non-aligned" (at least one chromosome away from the metaphase plate) and results from 25 oocytes for each condition are shown as % of total.

(G) Monopolar spindles arrested at metaphase were obtained from *klp-18(ok2519)* worms (KLP-18 is an essential protein to achieve bipolarity) in the presence of *emb-30(RNAi)*, either in the absence or presence of *smo-1(RNAi)*. α-tubulin is shown in green, DNA in blue, and SEP-1 (as a marker for metaphase) in magenta. The scale bar represents 2 μm.

(H) The 3D distance (d) between the spindle pole and the middle of each bivalent was measured (number of measurements are shown for each condition as "n"), and the results are represented in a dot plot with the median shown as a black horizontal line. The results were analyzed with the Kruskal-Wallis test, followed by Dunn's post-test.

RESULTS

SUMO in the *C. elegans* Germline

While sumoylation plays a role in meiotic chromosome pairing during prophase in yeast, this is not conserved in nematodes, where synapsis occurs normally in the absence of SUMO (Bhalla et al., 2008). While SUMO knockout worms have severe germline defects (Broday et al., 2004), we found that in SUMO E3 ligase *gei-17* −/− worms, some oocytes mature in spite of the defective germline and accumulate the CPC protein ICP-1 in the mid-bivalent and the cohesin REC-8 between homologous chromosomes and sister chromatids (Figure S1A). As opposed to the SC components SYP-1 and HTP-3, SUMO displays a diffuse localization in pachytene nuclei (Figures 1A, 1B, and S1B). As expected (Bhalla et al., 2008), RNAi-mediated depletion of GEI-17 affected neither chromosome synapsis, as evidenced by SYP-1 staining (Figure 1C), nor crossover designation (Figures S1C and S1D). The diffuse SUMO signal in pachytene nuclei remained after GEI-17 depletion (Figure S1E), likely corresponding to unconjugated SUMO and/or SUMO conjugated by the action of other E3 ligase(s). Enzymes of the SUMO pathway do not co-localize with DNA during pachytene: UBC-9 co-localizes with the nuclear envelope, while GEI-17 accumulates on the inner side of the nuclear envelope (Figure S1F). The SUMO protease ULP-1 also localizes in the nuclear envelope (data not shown). These results show that meiosis can proceed through pachytene when GEI-17-mediated SUMO conjugation is compromised.

Lack of Sumoylation Affects Chromosome Congression

Live imaging experiments using mCherry-H2B showed that in the absence of GEI-17, chromosomes often failed to align during metaphase I (Figure S2A). To robustly characterize this

phenotype, we used an anaphase promoting complex temperature-sensitive allele: *emb-27* (Golden et al., 2000), focusing on the oocyte closest to the spermatheca to avoid indirect effects derived from prolonged arrest. In the absence of GEI-17, chromosome alignment was compromised, as evidenced by the presence of one or two chromosomes closer to one of the spindle poles (Figures 1D, yellow arrows, 1E, and 1F). This phenotype is reminiscent of *klp-19(RNAi)* (Wignall and Villeneuve, 2009), consistent with a plus-end-directed force defect. As monopolar spindles allow the contributions of plus- and minus-end-directed forces to be evaluated (Muscat et al., 2015; Wignall and Villeneuve, 2009), we generated metaphase I-arrested monopolar spindles. Metaphase I chromosomes localize close to the plus-end of microtubules (Figure 1G) (Wignall and Villeneuve, 2009), but knock down of SUMO, UBC-9, or GEI-17 reduced the chromosomes-to-pole distance (Figures 1G and 1H). Separase (SEP-1) staining was used to confim that oocytes were in metaphase I (Bembenek et al., 2007; Muscat et al., 2015). Thus chromosome congression fails in the absence of sumoylation due to a defect in plus-end directed forces.

SUMO Localizes to a Ring-Shaped Structure during Meiosis

mCherry-SUMO strongly concentrates at the midbivalent in metaphase I and within sister chromatids in metaphase II (Figures 2A and S2B). During anaphase I and II, SUMO is detected between the segregating chromosomes and chromatids, respectively (Figures 2A and S2B) and becomes diffuse within the spindle in late anaphase (Figure 2A). Endogenous SUMO also concentrates in the midbivalent during metaphase I in a ring-shaped pattern (Figures 2B, 2C, and S2C) and partially co-localizes with microtubule bundles (Figures 2D and S2D). The same localization pattern was observed for UBC-9 (Figure 2E) and GEI-17 (Figure 2F). Endogenous, GFP-tagged GEI-17 localization is also dynamic being present in the midbivalent before localizing to the spindle midzone and then fading away by late anaphase (Figure 2G). This suggests that active sumoylation takes place within the RC and that one or more microtubule-associated proteins are SUMO substrates.

SUMO Conjugation within the Ring Complex

Knock down of GEI-17 dramatically reduces SUMO localization at the midbivalent (Figures 2H and 2I), although residual SUMO remains at the midbivalent and spindle (Figure 2I; mCherry channel “re-scaled”). To test if conjugation is required for the formation of the midbivalent SUMO ring, we expressed a GFP-SUMO mutant with the C-terminal Gly-Gly sequence mutated to Gly-Ala [“GFP-SUMO(GA)”] to block conjugation to substrates (Pelisch et al., 2014). GFP-SUMO(GA) localization to the midbivalent is dramatically reduced (Figure 2J), and residual localization can only be observed by rescaling the GFP fluorescence (Figure 2K). Thus, conjugation is the primary determinant for SUMO localization in the RC during meiosis, although a role for non-covalent SUMO interactions is also suggested. Supporting this argument, knock down of the SUMO E2 enzyme, UBC-9, not only inhibits SUMO concentration in the midbivalent, but also abolishes recruitment of the E3 ligase GEI-17 (Figure 2L). Thus, SUMO conjugation is required for E3 ligase recruitment and for SUMO to concentrate on the ring.

The Kinesin KLP-19 Is a Substrate for SUMO Modification and Exhibits SUMO-Dependent Localization

To search for SUMO substrates during meiosis, we adapted to *C. elegans* a proteomics approach successfully employed in human cells (Tammsalu et al., 2014, 2015). To identify sumoylation sites in vivo, we generated worms expressing His_6-tagged SUMO with a Leu to Lys substitution preceding the C-terminal diGly motif (L88K) in the germline and early embryos (Figure S3A). After Ni-NTA purification and Lys-C digestion, the mutant SUMO leaves a GG remnant on substrate lysines that facilitates peptide enrichment with an anti-K-ε-GG antibody (Figure S3B) (Tammsalu et al., 2014, 2015). Conjugation of SUMO(L88K) to substrates in vitro is indistinguishable from wild-type SUMO (Figures S3C and S3D) and is also conjugated in vivo (Figure S3E). Among the in vivo substrates, we obtained evidence for modification of KLP-19 within its C-terminal region (Figure S3F). Mass spectrometry analysis of in vitro sumoylated KLP-19 showed that Lys 873, contained in the peptide identified in vivo, is a SUMO modification site in KLP-19 (Figures S3G–S3I), although other lysines were identified as SUMO acceptors (Figure S7). SUMO and KLP-19 both localize within the midbivalent (Figures 3A and 3B, orange arrowhead), while a small fraction of KLP-19 also localizes to kinetochores (Figures 3A and 3B, yellow arrowhead). Juxtaposition of SUMO and KLP-19 in vivo was confirmed by proximity ligation assays (PLA) and is dependent on the SUMO E3 ligase GEI-17 (Figure 3C). To confirm that KLP-19 is a SUMO substrate, we performed in vitro sumoylation assays using bacterially expressed, purified proteins (Figures S4A–S4D). Full-length, untagged KLP-19 is efficiently modified by SUMO in a GEI-17-dependent manner (Figures 3D and S4E). As the identified SUMO modification site in vivo localizes within the C-terminal region of KLP-19, we performed in vitro sumoylation reactions using KLP-19(651–1,083), which excludes the motor and coiled-coil domains. KLP-19(651–1,083) is efficiently sumoylated in a GEI-17-dependent manner (Figures 3E and S4F), and we confirmed that the slower migrating species corresponded to SUMO-modified KLP-19 using two-color western blot (Figures 3F and S4G). Importantly, mutation of lysine 873 to arginine within KLP-19 (K873R) drastically reduced SUMO modification of KLP-19 (Figures 3F, S4G, and S4H). Co-depletion of GEI-17 and KLP-19 followed by quantification of the congression defect indicated that the two proteins act on the same genetic pathway (Figure S5A). Accumulation of KLP-19 in the RC is drastically reduced in the absence of GEI-17 (Figures 3G, orange arrow, and 3H), while the faint kinetochore signal is unaffected (Figure 3G, yellow arrows). GEI-17 or UBC-9 depletion revealed that in the absence of sumoylation, KLP-19 localized partially in kinetochores (Figure 3I, yellow arrows) and in thread-like structures that did not co-localize with microtubule bundles (Figure 3I, cyan arrows). Interestingly, kinetochore proteins have been shown to concentrate not only in the classical cup-shaped structures surrounding the bivalents, but also in

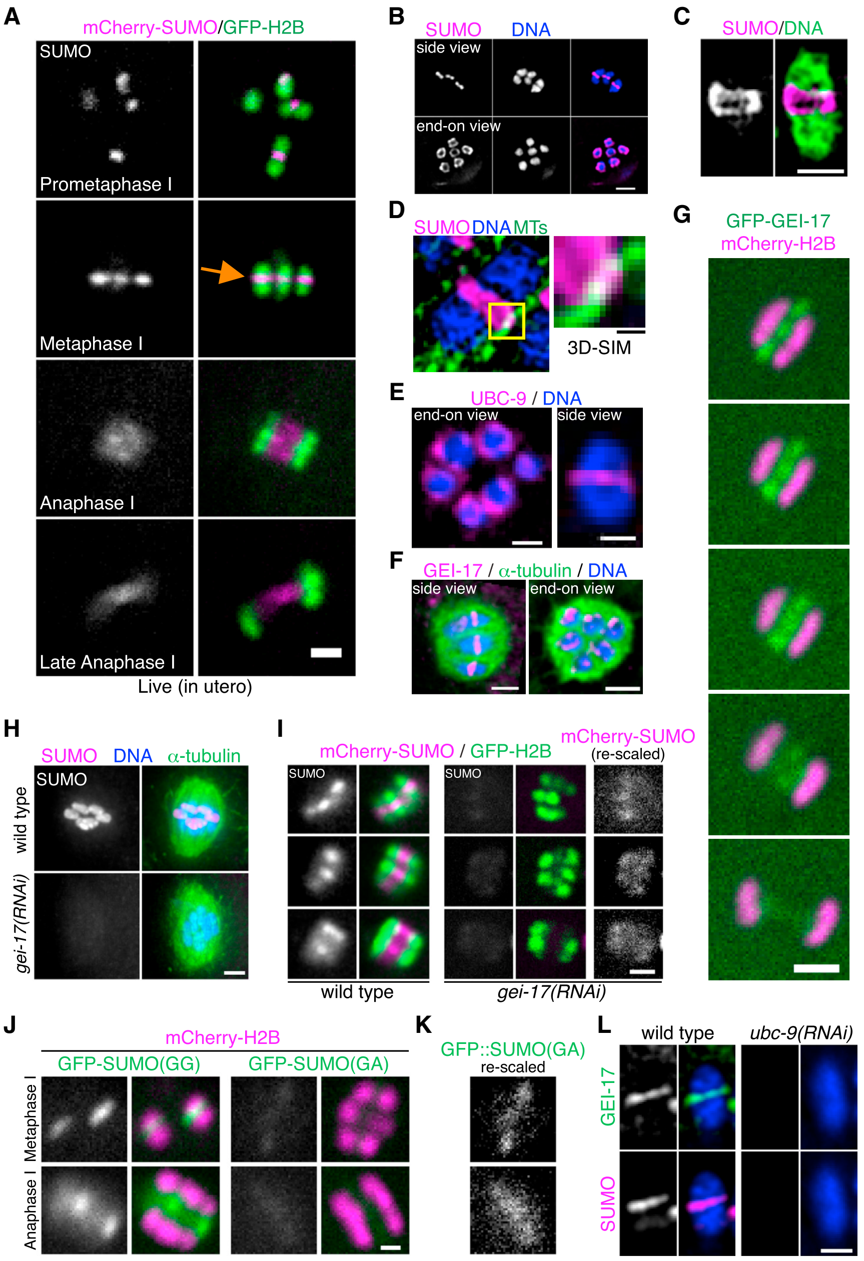

Figure 2. SUMO Conjugation during Meiosis

(A) Meiosis I in worms expressing mCherry-SUMO/GFP-H2B was followed by in utero time lapse. The still images of the different stages are shown with SUMO colored in magenta and H2B in green. The scale bar represents 2 μm. See also Figure S2B.

(B) The upper row displays a side view of the bivalents in metaphase I with SUMO shown in magenta and DNA in blue in the merged image. The lower row displays another set of bivalents in metaphase I from an end-on view. The scale bar represents 2 μm.

(C) 3D-SIM image of a single bivalent stained with SUMO (magenta) and DNA (green) is shown. The scale bar represents 1 μm. See also Figure S2C.

(D) 3D-SIM was used to analyzed a metaphase I meiotic spindle showing SUMO in magenta, α-tubulin in green, and DNA in blue. The area delimited by the yellow square is enlarged on the right. The scale bar represents 0.2 μm. See also Figure S2D.

(E) The SUMO E2 conjugating enzyme UBC-9 also localizes to the ring-shaped midbivalent structure. An end-on view of the whole set of chromosomes is displayed on the left and a side view on a single bivalent is shown on the right. The scale bar represents 1 μm.

(F) GEI-17 is shown in magenta, along with microtubules in green, and DNA in blue. The left image shows a side view of the spindle, while the right image displays an end-on view. The scale bar represents 2 μm.

(G) The localization of GEI-17 was followed through anaphase I by tagging the endogenous protein with GFP (see Experimental Procedures). Meiosis was followed as explained in (A). The scale bar represents 2 μm.

(H) Wild-type and *gei-17(RNAi)* embryos were fixed and stained with SUMO (magenta), DNA (blue), and α-tubulin (green). The scale bar represents 2 μm.

(I) Worms expressing mCherry-SUMO and GFP-H2B were fed control (wild-type) or *gei-17(RNAi)* and oocyte meiosis was recorded in utero. The scale bar represents 2 μm. The mCherry signal from *gei-17(RNAi)* oocytes was re-scaled and shown on the right.

(J) The conjugation dependency on SUMO localization was analyzed by comparing the localization of GFP-tagged SUMO(GG) and the non-conjugatable form SUMO(GA). The stills from live imaging experiments are shown for metaphase and anaphase from meiosis I. The scale bar represents 1 μm.

(K) The GFP fluorescence from GFP-SUMO(GA) was re-scaled to enhance detection of the small amount of SUMO remaining.

(L) GEI-17 recruitment to the midbivalent is UBC-9-dependent. The worms were fed control or *ubc-9(RNAi)* and stained for GEI-17 (green), SUMO (magenta), and DNA (blue). The channel corresponding to SUMO fluorescence was re-scaled as in (D). The scale bar represents 1 μm.

the so-called "linear elements" within the spindle and the cell cortex (Figure 3J) (Dumont et al., 2010; Monen et al., 2005). KLP-19 is not only present in the linear elements of the spindle, but also at the cell cortex (Figure S5D, cyan arrows). While mutation of Lys 873 to Arg in KLP-19 did not significantly affect its localization, general perturbation of the SUMO conjugation pathway lead to significant re-localization of KLP-19 away from the RC toward kinetochores and linear elements. Consistent with KLP-19 being a major SUMO substrate, *klp-19(RNAi)* leads to reduced ring localized SUMO (Figures S5B and S5C). Thus, KLP-19 is a SUMO substrate and sumoylation controls KLP-19 recruitment to the RC in vivo.

Sumoylation Is Essential for the Assembly of the Ring Complex

We then tested whether two other central RC components, BUB-1 and AIR-2, relied on sumoylation as a recruitment signal. As reported (Dumont et al., 2010; Wignall and Villeneuve, 2009), midbivalent localization of BUB-1 is dependent on ICP-1, but not on KLP-19 (Figure 4A). Depletion of *ubc-9* leads to a complete

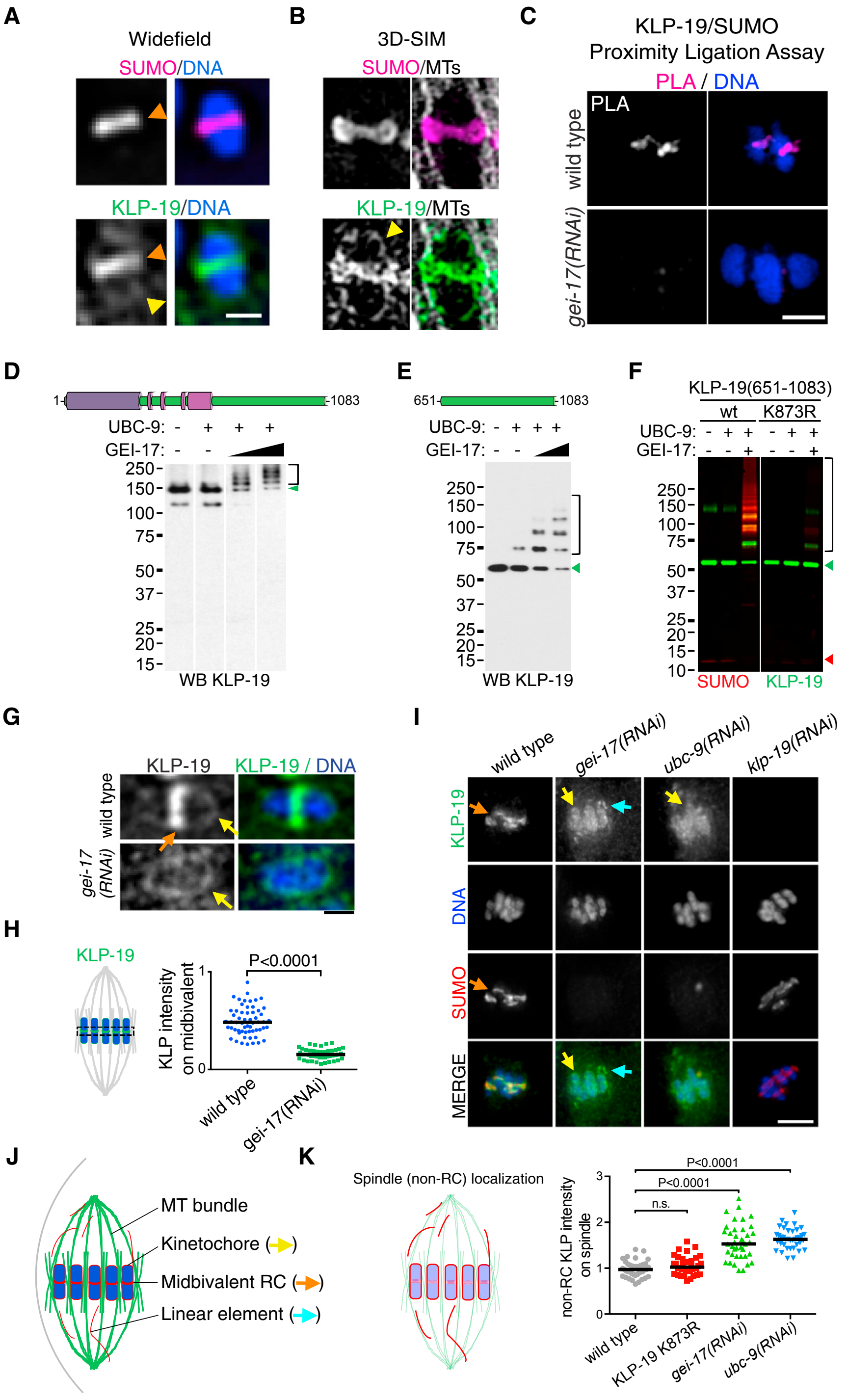

Figure 3. KLP-19 Is a SUMO Substrate and Its Ring Localization Depends on SUMO Conjugation

(A) Widefield images of a single bivalent stained for SUMO (top) and KLP-19 (bottom). The yellow arrow points to the kinetochore, while the orange arrow marks the midbivalent. The scale bar represents 1 μm.

(B) 3D-SIM images of a single bivalent stained for SUMO (magenta) or KLP-19 (green) and α-tubulin (gray).

(C) Proximity ligation assay of the KLP-19-SUMO interaction in control and *gei-17(RNAi)* worms. The bivalents during the first meiotic division are shown. The scale bar represents 2 μm.

(D) The cartoon depicts the domain architecture of KLP-19. The motor domain is shown in purple and the putative coiled-coil domains in pink. Full-length, recombinant KLP-19 was incubated in the presence of SUMO (20 μM), E1 enzyme (100 nM), UBC-9 (140 nM), and GEI-17 (12.5 and 25 nM). An aliquot of the reaction was run on an SDS-PAGE, and western blot was performed with an anti-KLP-19 antibody. The green arrowhead points to unmodified KLP-19, and the square bracket denotes SUMO-modified KLP-19. See also Figures S4A, S4B, S4D, and S4E.

(E) In vitro SUMO conjugation reactions were performed with KLP-19(651–1,083) as substrate. The reactions were incubated in the presence of SUMO (20 μM), E1 enzyme (100 ng), UBC-9 (140 nM), and increasing amounts of GEI-17 (12.5 and 25 mM). The reactions were developed as in (A). The green arrowhead points to unmodified KLP-19, and the square bracket denotes SUMO-modified KLP-19. See also Figures S4A, S4C, S4D, and S4F.

(F) Wild-type and K873R KLP-19(651–1,083) were subject to GEI-17-dependent in vitro SUMO conjugation, using the same conditions as above and 12.5 mM GEI-17. The reactions were then run on a gel, transferred to a membrane, and developed by two-color near-infrared western blotting (LICOR). KLP-19 is shown in green and SUMO in red. The red arrowhead indicates free SUMO; the green arrowhead points to unmodified KLP-19; and the square bracket denotes SUMO-modified KLP-19. See also Figures S3A–S3I, S4G, and S4H.

(G) KLP-19 localization in the midbivalent is dependent on GEI-17. Metaphase I-arrested oocytes were stained for KLP-19, SUMO, and DNA as indicated. The yellow arrow points to the kinetochore, while the orange arrow marks the midbivalent.

(H) Quantitation of the KLP-19 intensity in the midbivalent was analyzed using the Mann-Whitney test. The black lines indicate the median.

(I) KLP-19 localization was analyzed after GEI-17 or UBC-9 depletion. An orange arrow signals KLP-19 on the midbivalent. The yellow arrow indicates kinetochore localization. The blue arrow indicates the linear elements (see main text for details). The scale bar represents 4 μm. See also Figure S5D.

(J) Schematic depicting the procedure for quantifying KLP-19 residing outside the RC (“non-RC”). The microtubule bundles, kinetochores, midbivalents, and linear elements are shown next to the colored arrows used throughout the figure to highlight them. The whole spindle area was selected using α-tubulin as a guide, and the midbivalents were selected from the DNA channel. After substracting the background-corrected midbivalent signal to the background-corrected spindle signal, we obtained the non-RC KLP-19 intensity.

(K) KLP-19 re-localizes to kinetochores and linear elements after UBC-9 or GEI-17 depletion. The data for non-RC KLP-19 intensity are shown as a dot plot, and the samples were compared using a Kruskal-Wallis test, followed by Dunn’s post-test. The black lines denote the medians.

Cell Press

loss of BUB-1 at the midbivalent without affecting its kinetochore localization (Figures 4A, S6A, and S6B). In the absence of BUB-1, SUMO can still be detected in the midbivalent (Figure 4B). Sumoylation does not regulate kinetochore assembly as assessed by NDC-80 localization (Figures 4C and S6C). PLA assays show that SUMO is in close proximity to both BUB-1 (Figure 4D) and AIR-2 (Figure 4E) within the RC. In the absence of sumoylation, AIR-2 still localized between homologous chromosomes, although to a lesser extent (Figures 4F and 4G). Another CPC component, ICP-1/INCENP and the CPC substrate phospho-H3(S10), re-localize from the midbivalent to chromosomes in the absence of GEI-17 (Figures 4H and 4I). The midbivalent is also strongly stained with the anti-mitotic phospho-proteins MPM-2 antibody (Kitagawa and Rose, 1999), where it specifically recognizes the RC (Muscat et al., 2015). Depletion of GEI-17 leads to a dispersion of the MPM-2 signal, further showing that abolishing GEI-17-mediated sumoylation leads to RC disruption (Figure S6D). Additionally, completely disassembling the CPC by means of *icp-1(RNAi)* leads to the loss of the SUMO signal in the midbivalent (Figure 4J). Thus, the CPC and sumoylation are required for the RC to assemble, and we propose that this SUMO-dependent CPC assembly provides the basic platform for other components to associate with the RC. Initial or "seed" SUMO modification is thus CPC-dependent and likely to occur within the CPC itself, possibly with AIR-2 as a substrate.

Acute, Germline-Specific Loss of GEI-17 Affects KLP-19 Recruitment to the Ring

While we propose that SUMO affects KLP-19 directly, this interpretation is complicated by the fact that BUB-1, required for KLP-19 recruitment, is absent from the RC upon GEI-17 depletion. To overcome this, we used CRISPR/Cas9-mediated genome editing to tag the endogenous copies of *gei-17* with a fragment encoding a fusion between GFP, FLAG, and an auxin-responsive degron sequence (Zhang et al., 2015) (Figure 5A). Addition of auxin for 1.5 hr leads to the loss of the GFP-GEI-17 signal from the germline (Figure 5B) and embryo (Figure 5C) in worms specifically co-expressing mRuby-TIR1

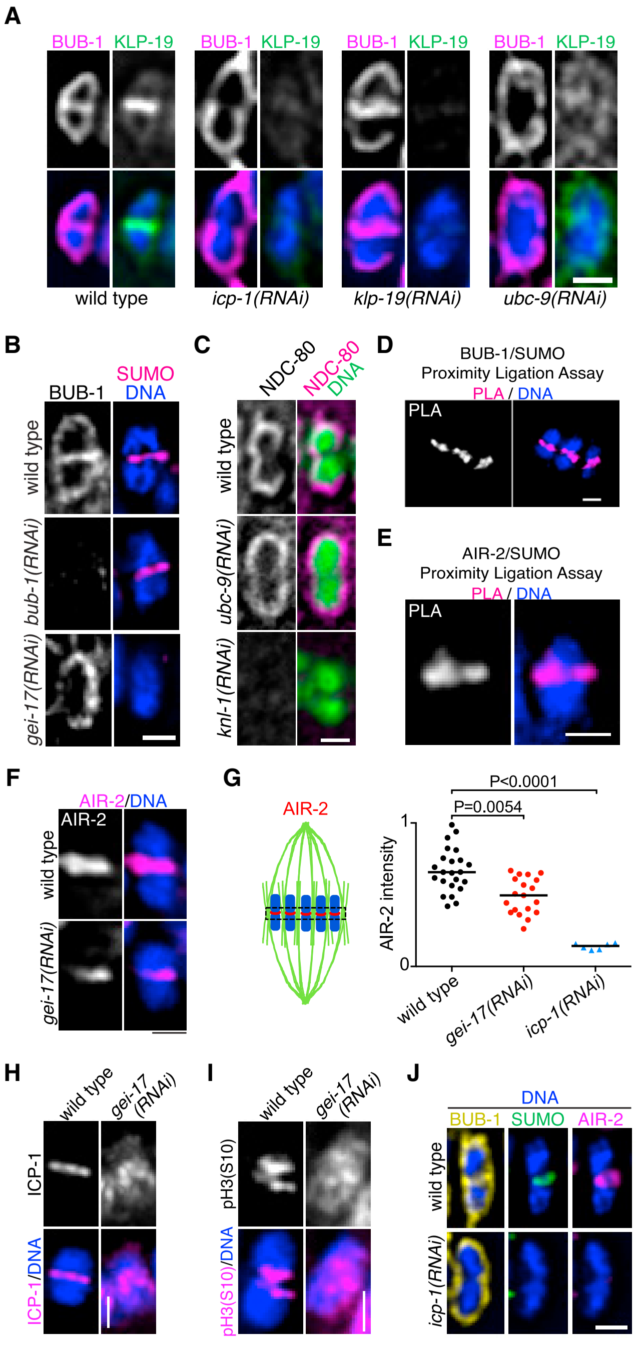

Figure 4. Ring Complex Assembly Depends on Sumoylation

(A) BUB-1 and KLP-19 localization were analyzed in control (wild-type), *ubc-9(RNAi)*, *klp-19(RNAi)*, and *icp-1(RNAi)* in *emb-27* oocytes arrested at metaphase I. The scale bar represents 1 μm. See also Figures S6A and S6B.

(B) The localization of BUB-1 and SUMO was analyzed as in (A) in control (wild-type), *bub-1(RNAi)*, and *gei-17(RNAi)* in *emb-27* oocytes arrested at metaphase I. The scale bar represents 1 μm.

(C) Localization of the kinetochore component NDC-80 was analyzed in the presence of control RNAi (wild-type), *ubc-9(RNAi)*, or *knl-1(RNAi)* a known regulator of kinetochore assembly. The scale bar represents 1 μm. See also Figure S6C.

(D) Proximity ligation assays were performed using BUB-1 and SUMO specific antibodies, and the PLA signal is shown in magenta and DNA in blue. The scale bar represents 1 μm.

(E) Proximity ligation assays were performed using AIR-2 and SUMO specific antibodies, and the PLA signal is shown in magenta and DNA in blue. The scale bar represents 1 μm.

(F) AIR-2 localization was in metaphase I-arrested oocytes from *emb-27* worms in the presence of control (wild-type) or *gei-17(RNAi)*.

(G) AIR-2 fluorescence intensity in the midbivalent was quantified in wild-type, *gei-17(RNAi)*, and *icp-1(RNAi)* oocytes. The AIR-2 intensity is shown in the dot plot graph, and the samples were compared using a Kruskal-Wallis test, followed by Dunn's post-test. The black lines denote the medians.

(H) The absence of GEI-17 affects ICP-1/INCENP localization. ICP-1 localization was analyzed in wild-type or *gei-17(RNAi)* oocytes as above. The scale bar represents 1 μm.

(I) The absence of GEI-17 affects phospho-Ser10-H3 localization. H3 phosphorylated on serine 10, known to be a CPC substrate, was analyzed in wild-type or *gei-17(RNAi)* oocytes as above. The scale bar represents 1 μm.

(J) The CPC is required for ring assembly. Single bivalents co-stained for BUB-1 (yellow), SUMO (green), AIR-2 (magenta), and DNA (blue), either from wild-type or *icp-1(RNAi)* oocytes are shown. The scale bar represents 1 μm.

(Zhang et al., 2015). Addition of auxin for 4 hr was sufficient to inhibit recruitment of KLP-19 to the midbivalent ring (Figures 5D and 5E) even though BUB-1 was still present (Figure 5F). However, re-scaling the SUMO channel fluorescence revealed that a small amount of SUMO could still be detected in ring-like structures, consistent with the persistence of other substrate(s) and/or non-covalent SUMO binding proteins (Figure 5E). Thus, sumoylation directly regulates KLP-19 recruitment to the midbivalent ring.

Ring Components Are Recruited in a Stepwise Fashion from Diakinesis to Prometaphase

Live imaging and immunostaining revealed that SUMO shifted from diffusely chromosomal to the midbivalent concomitant with oocyte NEBD (Figures 6A and 6B). Whereas ICP-1 was detected at the midbivalent in the −2 oocyte (Bishop and Schumacher, 2002; Schumacher et al., 1998), SUMO only concentrated in the midbivalent in the −1 oocyte (Figure 6C). This concentration of SUMO was dependent on conjugation as it was abrogated by *ubc-9(RNAi)* (Figure 6D). GEI-17 was also recruited to the midbivalent in post-NEBD oocytes, suggesting that GEI-17-mediated sumoylation within the ring was initiated during fertilization (Figure 6E) and precedes KLP-19 concentration in the midbivalent (Figure 6F) (Powers et al., 2004). In fixed −1 oocytes, BUB-1 is predominantly localized in kinetochores (Figure 6G), while live imaging on oocytes expressing mCherry-BUB-1 and GFP-SUMO showed that BUB-1 was initially recruited to the kinetochore, while SUMO was already concentrated in the midbivalent (Figure 6H, top image). By metaphase I, however, BUB-1 has been recruited to the RC (Figure 6H, bottom image). Thus SUMO modification of RC components precedes BUB recruitment to the midbivalent. These results show that the RC is assembled in a stepwise manner with assembly initiated prior to fertilization.

BUB-1 Interacts with SUMO-Modified KLP-19 and GEI-17

The observation that a mutant SUMO incapable of conjugating to substrates partially localizes to the RC points to the existence of non-covalent SUMO interactions occurring within the RC. Having shown that KLP-19, AIR-2, and GEI-17 are conjugated to SUMO (Figure 7A), we searched for an RC component that could interact non-covalently with SUMO. An obvious candidate is the SUMO E3 ligase GEI-17, whose yeast and mammalian orthologs have SIMs (Jentsch and Psakhye, 2013). Another candidate is BUB-1, a protein that localizes in kinetochores and the RC and is essential for KLP-19 recruitment to the RC. Inspection of BUB-1 amino acid sequence revealed putative SIMs,

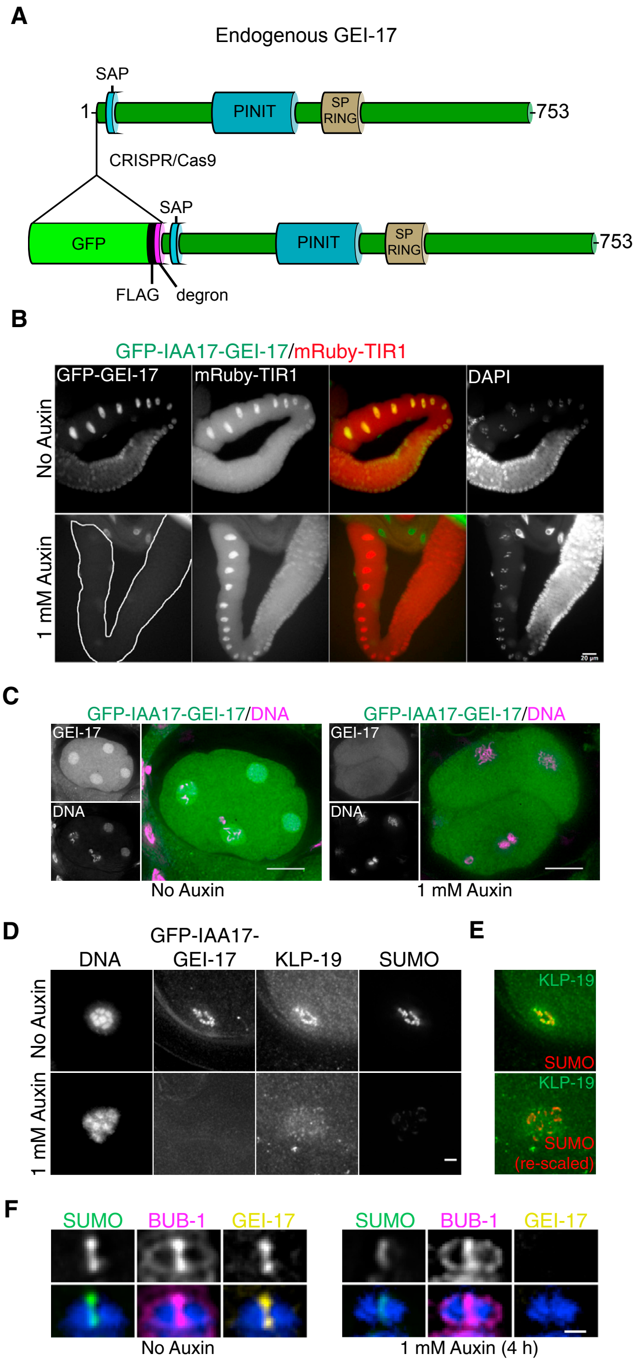

Figure 5. Tissue-Specific, Auxin-Induced Degradation of GEI-17

(A) Schematic of the generation of GFP-FLAG-degron-tagged endogenous GEI-17 by CRISPR/Cas9-mediated genome editing. The SAP and PINIT domains are highlighted, as well as the SP-RING. The numbering corresponds to GEI-17 isoform f (Uniprot Q94361-2).

(B) Gonads from untreated or auxin-treated (1.5 hr) worms were dissected, fixed, and imaged for GFP (GEI-17) and mRuby (TIR1) fluorescence. The scale bar represents 20 μm.

(C) Embryos from untreated or auxin-treated (1.5 hr) worms were fixed and imaged for GFP-GEI-17 (green) and DNA (magenta). The scale bar represents 10 μm.

(D) Metaphase I oocytes from untreated or auxin-treated worms were fixed and imaged for GFP-GEI-17, KLP-19, and SUMO, along with DNA. Treatment with auxin for 4 hr is enough to reduce SUMO levels and also leads to a diffuse KLP-19 localization.

(E) Re-scaling of the SUMO fluorescence shows that while some SUMO is still present, there is no specific co-localization with KLP-19. The scale bar represents 2 μm.

(F) Metaphase I oocytes from untreated or auxin-treated worms were fixed and imaged for GFP-GEI-17 (yellow), BUB-1 (magenta), and SUMO (green), along with DNA. After treatment with auxin for 4 hr, BUB-1 is still present in the midbivalent. The scale bar represents 1 μm.

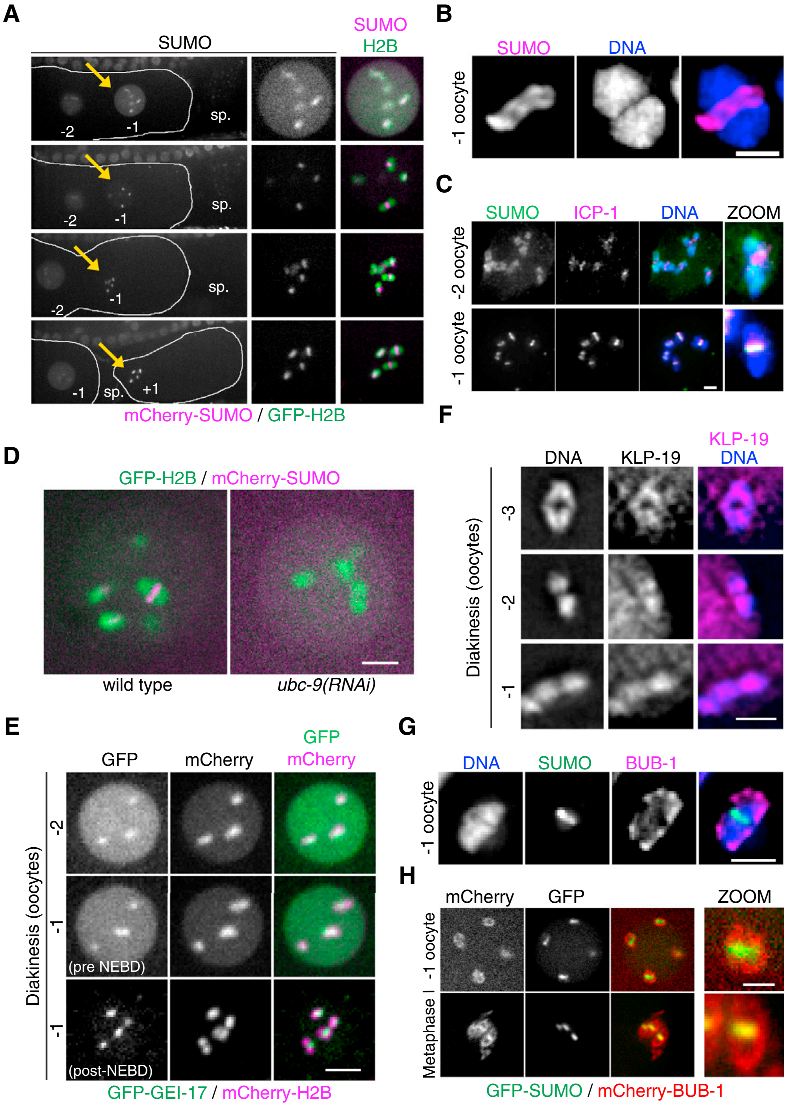

Figure 6. Ring Complex Assembly Starts during Diakinesis

(A) An oocyte from a worm expressing mCherry-SUMO and GFP-H2B was recorded during fertilization. The yellow arrow in each image points to the oocyte that was followed. "−2" and "−1" stand for, the −2 and −1 oocytes, respectively; spermatheca, sp.; "+1" is the fertilized oocyte.

(B) SUMO concentrates in the short axis of the bivalent (midbivalent). A bivalent within a −1 oocyte is shown with SUMO in magenta and DNA in blue. The scale bar represents 1 μm.

(C) The CPC component ICP-1 (magenta) localizes to the midbivalent as early as the −2 oocyte, as opposed to SUMO (green) that only concentrates in the midbivalent in the −1 oocyte. The scale bar represents 2 μm.

(D) SUMO concentration in the midbivalent in early oocytes is dependent on UBC-9. The −1 oocyte was followed as in (A) in wild-type or *ubc-9(RNAi)* worms. An image after NEBD is shown. The scale bar represents 2 μm.

(E) GEI-17 concentrates on the midbivalent after oocyte NEBD. Worms expressing endogenous GEI-17 tagged with a GFP-FLAG-degron cassette together with mCherry-H2B were analyzed as in (A) and (E). The scale bar represents 5 μm.

(F) KLP-19 (magenta) localization during diakinesis was analyzed by immunostaining of dissected gonads. The single bivalents from the three most mature oocytes are shown. The scale bar represents 2 μm.

(G) BUB-1 is first recruited to the kinetochores. BUB-1 (magenta) localization along with that of SUMO (green) was analyzed by immunostaining of dissected gonads. A single bivalent from the −1 oocyte is shown. The scale bar represents 2 μm.

(H) An oocyte from worms expressing GFP-SUMO and mCherry-BUB-1 was followed as in (A). In the upper image, the −1 oocyte has gone through NEBD (as judged by the SUMO staining), while the lower image shows bivalents in metaphase of meiosis I. The scale bar represents 1 μm.

mostly concentrated outside of the C-terminal kinase domain (Figure 7B). We expressed the fragment (1–689) containing the putative SIMs with a His_6 tag to perform pull-down assays (Figure 7B). BUB-1(1–689) preferentially interacts with SUMO-modified KLP-19 (Figure 7C) and GEI-17 (Figure 7D). When analyzing binding reactions with a SUMO antibody, it was apparent that BUB-1 binds high molecular weight SUMO conjugates, but not free SUMO (Figure 7E). We then tested the functionality of the putative SIMs in BUB-1 and GEI-17 using MBP-fusion proteins (Figure 7F) in pull-down assays with sumoylated GEI-17 or KLP-19(651–1,083). While BUB-1(2–551) readily interacted with SUMO-modified GEI-17 and SUMO-modified KLP-19, mutation of all five putative SIMs abolished this interaction (Figures 7G and 7H). Additionally, a fragment containing the two predicted high-affinity SIMs in GEI-17 (aa 423–602 in isoform f), pulled down higher molecular weight forms of both sumoylated GEI-17 and sumoylated KLP-19 and these interactions were strictly SIM-dependent (Figures 7G and 7H). Thus, both SUMO substrates and non-covalent SUMO binders co-exist within the ring. If BUB-1 is important for non-covalent SUMO binding in vivo, then depletion of BUB-1 is predicted to affect non-covalent SUMO recruitment to the RC. To test this, we used a mutant version of SUMO that cannot conjugate to substrate proteins (SUMO(GA), see Figures 2J and 2K) and thus provides a readout for non-covalent SUMO interactions. Consistent with BUB-1 interacting non-covalently with SUMO/sumoylated proteins, GFP-SUMO(GA) recruitment to the RC during meiosis I was greatly diminished in the absence of BUB-1 (Figure 7I, yellow arrows). We then sought to test whether KLP-19 localization in the RC is SIM dependent. To this end, we injected a SIM containing peptide (Bruderer et al., 2011) (or a control peptide) into the gonads of *emb-27* worms and metaphase I-arrested oocytes were analyzed 24 hr later (Figure 7J). While SIM injection did not dramatically affect KLP-19 recruitment to the RC, an increase in KLP-19 in kinetochores and linear elements was observed (Figure 7J). This result, while smaller in magnitude, resembles the effect obtained after knocking down either GEI-17 or UBC-9 (Figures 3I–3K). Thus, we conclude that both covalent SUMO conjugation and

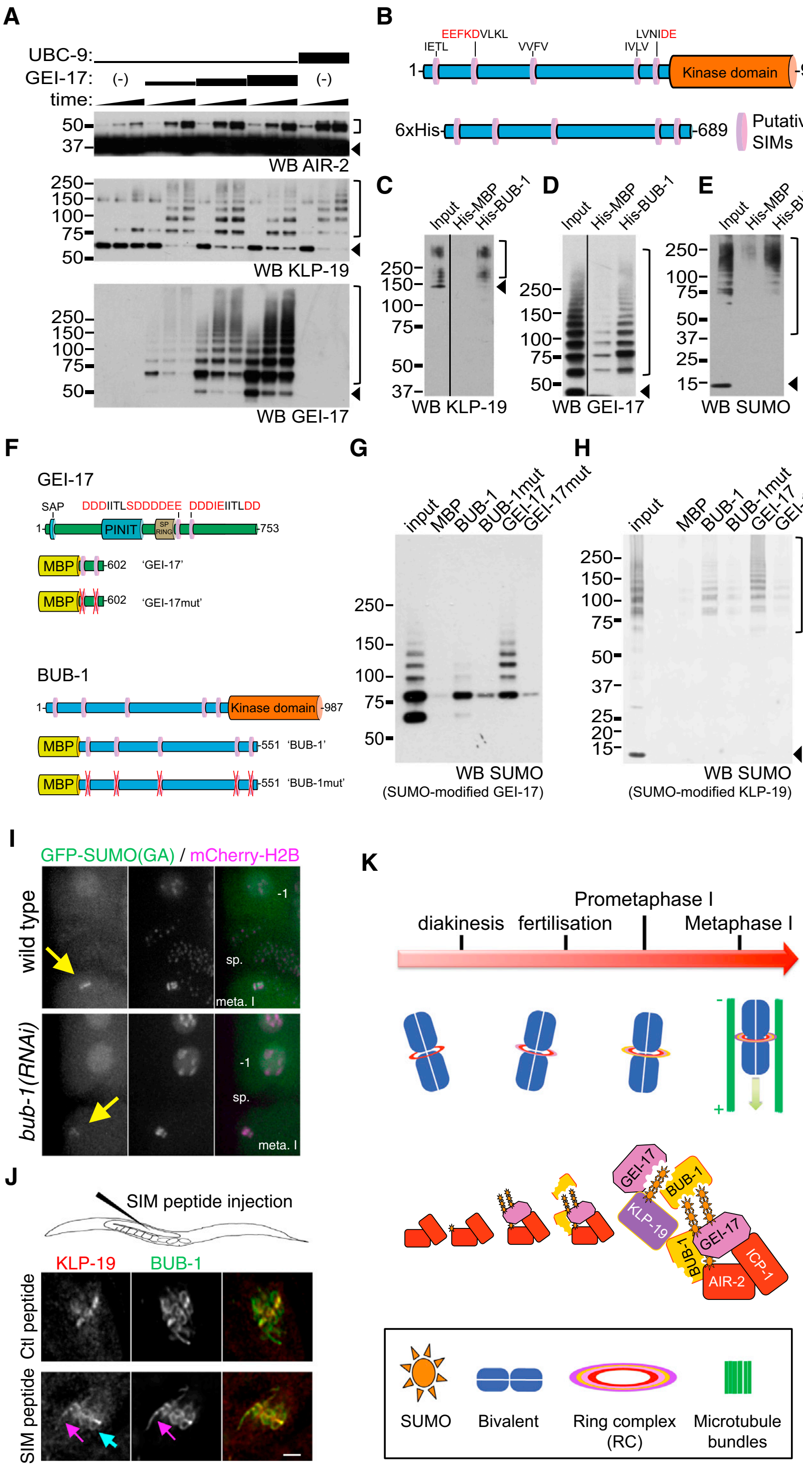

Figure 7. BUB-1 Binds to SUMO-Modified KLP-19 and GEI-17

(A) Reconstituted in vitro sumoylation reactions were performed as described in Experimental Procedures. While AIR-2 is modified by a single SUMO (doublet corresponds to two mono-sumoylated forms; Pelisch et al., 2014), KLP-19 and GEI-17 undergo multiple modifications. The arrowheads point to unmodified substrates, while open brackets indicate the SUMO-modified substrate.

(B) Schematic of BUB-1 with its C-terminal kinase domain (top) and the hexahistidine-tagged BUB-1 fragment used for the pull-down assays shown in images (C)–(E).

(C) 6xHis-MBP ("His-MBP", control) and 6xHis-BUB-1(1–689) ("His-BUB-1") were used in Ni-NTA pull-down assays using SUMO-modified full-length KLP-19 as input. The binding reactions were run on SDS-PAGE, and western blotting was performed with an antiKLP-19 specific antibody. The arrowhead points to the position of unmodified KLP-19, and the square bracket show the bands corresponding to SUMO-modified KLP-19.

(D) Same as (C), but using SUMO-modified GEI-17 as input material. In this case, a GEI-17 specific antibody was used. The arrowhead points to the position of unmodified GEI-17, and the square bracket show the bands corresponding to SUMO-modified GEI-17.

(E) Pull-down assays were performed using GEI-17-mediated SUMO-modified full-length KLP-19 as input. The reactions were analyzed as above using a SUMO-specific antibody. The arrowhead points to the position of unconjugated SUMO, and the square bracket indicates the position of conjugated SUMO (to KLP-19 and GEI-17).

(F) Schematic of the GEI-17 and BUB-1 fragments containing the putative SIM motifs used in the pull-down experiments.

(G) SUMO-modified GEI-17 was used for pull-down assays with MBP-tagged BUB-1, BUB-1mut, GEI-17, and GEI-17mut. Input and pulled-down materia were analyzed by western blot with an anti-SUMO antibody.

(H) SUMO-modified KLP-19 was used for pull-down assays with MBP-tagged BUB-1, BUB-1mut, GEI-17, and GEI-17mut. Input and pulled-down materia were analyzed by western blot with an anti-SUMO antibody. The arrowhead indicates the presence of free SUMO, and the square bracket denotes SUMO-conjugated KLP-19(651–1,083).

(I) Meiosis was followed in utero in control (wild-type) or *bub-1(RNAi)* worms expressing GFP-SUMO(GA) and mCherry-tagged H2B. The oocytes at metaphase I are pointed out by a yellow arrow. "sp." denotes the location of the spermatheca; "meta. I" indicates the location of the oocyte at metaphase I; and "−1" shows the location of the maturing oocyte closest to the spermatheca before fertilization, the −1 oocyte.

(J) *emb-27* worms were injected with a SIM-containing or a control peptide, and KLP-19 and BUB-1 localization was assessed in metaphase I-arrested oocytes. The pink arrow points to a linear element, and the blue arrow points to a cup-shaped kinetochore. The scale bar represents 2 μm.

(K) Proposed model for SUMO-mediated control of chromosome congression.

non-covalent SUMO interaction contribute to stable RC assembly. Notably, the dynamic and reversible nature of these interactions guarantees that the ring can be easily disassembled, an event required as anaphase progresses.

DISCUSSION

We provide evidence that SUMO modification plays an important role during female meiotic chromosome congression in *C. elegans* by regulating RC assembly (Figure 7K). AIR-2/Aurora B and ICP-1/INCENP localize to the midbivalent during diakinesis in a SUMO-independent manner, providing the basic platform of the RC. The SUMO E3 ligase GEI-17 then joins the complex during oocyte nuclear envelope breakdown and triggers SUMO conjugation, likely of AIR-2 (and/or other yet to be identified RC components). Then BUB-1, which is already in kinetochores, joins the RC in a GEI-17-dependent manner. Finally, the kinesin KLP-19 is modified by SUMO in a GEI-17-dependent manner and is recruited to the midbivalent RC in a sumoylation-dependent fashion. As the SIMs in both GEI-17 and BUB-1 allow them to interact with SUMO-modified GEI-17 and KLP-19 (and likely other SUMO-modified proteins), they act as central players in the formation of this meiosis-specific SUMO-SIM network.

Both SUMO conjugation and non-covalent SUMO interaction are required for proper RC assembly. Indeed, when sumoylation is inhibited by UBC-9 or GEI-17 depletion, KLP-19 ‘‘diffuses away’’ from the RC and displays a localization pattern characteristic of outer kinetochore proteins, localizing in cup-shaped structures surrounding the bivalents and in linear elements in the spindle and cell cortex (Dumont et al., 2010; Monen et al., 2005). In this model, SUMO could be key in regulating the partitioning of proteins between the RC and other neighboring structures.

Many aspects of the RC function during meiosis remain to be elucidated. This structure is functioning as a signaling hub, where phospho-proteins, as well as SUMO-modified proteins concentrate. The enzymes that catalyze these modifications, like the kinases AIR-2/Aurora B and BUB-1 and the SUMO E3 ligase GEI-17, localize within the RC themselves. This suggests that active protein modification takes place within the RC. The RC was shown to disassemble during anaphase, so future studies are needed to address the role of SUMO proteases in RC disassembly. Interestingly, it was recently reported that protein phosphatase 1 recruitment by the nucleoporin MEL-28 directs outer kinetochore disassembly, an event required for proper meiotic chromosome segregation (Hattersley et al., 2016). We propose that PTMs, and interactions among them, will be key regulators of the highly dynamic changes that take place within the meiotic spindle.

A remarkable feature of the RC is that within a 30-min period, it undergoes two cycles of assembly/disassembly linked to two waves of SUMO modification/deconjugation that are regulated with exquisite precision both temporally and spatially. During oocyte nuclear envelope breakdown, RC becomes SUMO modified and an assembly feedforward cycle starts. However, SUMO is removed and the RC disassembles during anaphase I, and this is followed by SUMO conjugation/RC assembly during prometaphase II and SUMO deconjugation/RC disassembly during anaphase II. However, this is not the end of the dynamic behavior, as we have shown that SUMO modification/deconjugation takes place during the first embryonic mitotic division (Pelisch et al., 2014). The ability of SUMO to function as a reversible molecular ‘‘glue’’ satisfies the need for this rapid assembly-disassembly cycles. Since the introduction of the protein group sumoylation concept (Psakhye and Jentsch, 2012), SUMO-mediated RC assembly provides the first example of a specialized complex within a multi-cellular organism assembled as a SUMO-SIM network under physiological conditions. Just as a balance of forces exists between kinesin-driven plus-end movement and dynein-mediated minus end forces (Muscat et al., 2015), a similar equilibrium may be mediated by SUMO E3 ligases and SUMO proteases. Indeed, the SUMO protease ULP-1 localizes to the RC (data not shown). In this context, while E3 activity prevails until metaphase, SUMO proteases are likely to predominate during anaphase, leading to ring disassembly. While it remains to be shown what signal(s) regulate the balance between E3 and protease activities, the presence of both E3 ligases and SUMO proteases would facilitate the assembly/disassembly cycles.

In budding yeast, SUMO co-localizes with the synaptonemal complex during pachytene and plays a role in chromosome synapsis (Cahoon and Hawley, 2016). However, SUMO is not essential for SC assembly in early pachytene in nematodes (Bhalla et al., 2008) and localizes mainly at the midbivalent, where key regulators of chromosome congression (like KLP-19) reside. As in nematodes, it has been shown in rat spermatocytes that SUMO does not co-localize with the synaptonemal complex during pachytene (Rogers et al., 2004), while depletion of SUMO or UBC9 caused abnormal spindle organization, and led to chromosome misalignment, segregation defects, and aneuploidy in rat oocytes (Yuan et al., 2014). Additionally, SUMO-1 concentrates in spindle poles and between segregating chromosomes in anaphase I, while SUMO-2/3 co-localized with condensed chromatin in mouse oocytes (Wang et al., 2010). This localization pattern is reminiscent of that of SUMO-1 and SUMO-2/3 during mitosis (Zhang et al., 2008), and the only SUMO isoform in nematodes displays a combination of these localization patterns during the first embryonic mitotic division (Pelisch et al., 2014). Many SUMO substrates are involved in chromatin structure and function (Cubeñas-Potts and Matunis, 2013), and KIF4A, the human ortholog of KLP-19, has been identified as a SUMO substrate in mitotic chromatin (Cubeñas-Potts et al., 2015). These observations support the notion that while precise mechanisms that guarantee proper chromosome orientation, congression, and segregation might differ between meiosis and mitosis and also among species, SUMO is likely a key contributor to a timely and accurate regulation of protein interactions within narrow spatial and temporal windows. In line with this, AIR-2/Aurora B shifts its localization from chromatin to the spindle midzone during mitotic anaphase and this transition is dependent on the SUMO protease ULP-4 (Pelisch et al., 2014). SUMO-SIM networks are likely to predominate when the equilibrium of a protein between two or more cell structures/protein complexes is subject to a fast dynamic regulation. Overall, we have provided evidence that

highly dynamic, coordinated, and spatially constrained sumoylation regulates chromosome congression during meiosis in *C. elegans* oocytes.

EXPERIMENTAL PROCEDURES

Worms

C. elegans strains were maintained according to standard procedures (Brenner, 1974). The strains used are listed in Supplemental Experimental Procedures. For RNAi treatment, bacterial clones expressing dsRNAs were obtained from a commercial library (Kamath and Ahringer, 2003).

CRISPR/Cas9

GEI-17 fused to GFP-FLAG-degron was generated by CRISPR (Dickinson et al., 2015). The degron sequence consisted of the 44-amino acid (aa) fragment of the *Arabidopsis thaliana* IAA17 protein (Morawska and Ulrich, 2013; Zhang et al., 2015).

Auxin Treatment

Auxin (IAA, Sigma-Aldrich) was used at 1 mM final concentration in standard NGM plates.

Antibodies

Antibodies against SMO-1, GEI-17, and UBC-9 were reported previously (Pelisch and Hay, 2016; Pelisch et al., 2014). AIR-2 and ICP-1 peptide antibodies were produced and affinity purified using previously described peptides (Burrows et al., 2006; Schumacher et al., 1998). Anti-KLP-19 serum (Powers et al., 2004) was subject to protein-A purification before use.

In Utero Embryo Live Imaging

Worms were picked into a solution of tricaine (0.1%) and tetramisole (0.01%), pipetted onto a 4% agar pad, covered with a coverslip, and imaged with a spinning-disk confocal microscope (MAG Biosystems) mounted on a microscope (IX81; Olympus) with a 100 × /1.45 Plan Apochromat oil immersion lens (Olympus), a camera (Cascade II; Photometrics), spinning-disk head (CSU-X1; Yokogawa Electric), and MetaMorph software (Molecular Devices).

Immunostaining

Immunofluorescence analysis was performed essentially as described (Pelisch and Hay, 2016; Pelisch et al., 2014).

Duolink In Situ Proximity Ligation Assay

Proximity ligation assays were performed as described (Pelisch and Hay, 2016; Pelisch et al., 2014).

Analysis of Mass Spectrometry Data

Raw mass spectrometry (MS) files were analyzed using MaxQuant software package (version 1.3.0.5) (Cox and Mann, 2008) and peak lists were searched with an integrated Andromeda search engine (Cox et al., 2011) against an entire *C. elegans* UniProtKB proteome.

Statistical Analysis

The different tests used throughout the study are detailed in Supplemental Experimental Procedures.

SUPPLEMENTAL INFORMATION

Supplemental Information includes Supplemental Experimental Procedures and seven figures and can be found with this article online at http://dx.doi.org/10.1016/j.molcel.2016.11.001.

AUTHOR CONTRIBUTIONS

F.P. conceived the project; designed, performed, and interpreted experiments; and wrote the manuscript. T.T. performed the diGly peptide purification experiments and mass spectrometry analysis. B.W. performed injections. E.G.J. purified recombinant proteins for pull-down assays. A.G. interpreted experiments and revised the manuscript. R.T.H. designed and interpreted experiments, supervised the project, and wrote the manuscript.

ACKNOWLEDGMENTS

We would like to thank Sadie Wignall for advice, strains, and for communicating results prior to publication and S. Köhler and A. Dernburg for sharing unpublished Cas9-mediated genome editing and auxin-induced degradation protocols. We also thank S. Stromme, M. Zetka, A. Desai, T. Hyman, B. Meyer, and A. Golden for reagents. This work was supported by the Wellcome Trust (098391/Z/12/7 to R.T.H. and 090944/Z/09/Z to A.G.), Cancer Research UK (C434/A13067 to R.T.H.), and the BBSRC (BB/J015199/1 to A.G. and R.T.H.). F.P. was the recipient of a Marie Curie fellowship (grant agreement 297881) and is a career investigator of the Argentinean Research Council (CONICET). T.T. was funded by the EU Initial Training Network (ITN) "UPSTREAM" (PITN-GA-2011-290257) and an ISSF grant funded by the Wellcome Trust (105606/Z/14/Z). Some nematode strains were provided by the CGC, which is funded by NIH Office of Research Infrastructure Programs (P40 OD010440). We acknowledge the Dundee Imaging Facility, which is supported by the "Wellcome Trust Technology Platform" award (097945/B/11/Z) and the "MRC Next Generation Optical Microscopy" award (MR/K015869/1). We also acknowledge the Tissue Imaging Facility, which is funded by a Wellcome Trust award (101468/Z/13/Z). A.G. is a Wellcome Trust Senior Biomedical Research Fellow. R.T.H. is a Senior Investigator of the Wellcome Trust.

Received: June 14, 2016
Revised: September 29, 2016
Accepted: October 31, 2016
Published: December 8, 2016

REFERENCES

Bembenek, J.N., Richie, C.T., Squirrell, J.M., Campbell, J.M., Eliceiri, K.W., Poteryaev, D., Spang, A., Golden, A., and White, J.G. (2007). Cortical granule exocytosis in C. elegans is regulated by cell cycle components including separase. Development *134*, 3837–3848.

Bhalla, N., Wynne, D.J., Jantsch, V., and Dernburg, A.F. (2008). ZHP-3 acts at crossovers to couple meiotic recombination with synaptonemal complex disassembly and bivalent formation in C. elegans. PLoS Genet. *4*, e1000235.

Bishop, J.D., and Schumacher, J.M. (2002). Phosphorylation of the carboxyl terminus of inner centromere protein (INCENP) by the Aurora B Kinase stimulates Aurora B kinase activity. J. Biol. Chem. *277*, 27577–27580.

Brenner, S. (1974). The genetics of Caenorhabditis elegans. Genetics *77*, 71–94.

Broday, L., Kolotuev, I., Didier, C., Bhoumik, A., Gupta, B.P., Sternberg, P.W., Podbilewicz, B., and Ronai, Z. (2004). The small ubiquitin-like modifier (SUMO) is required for gonadal and uterine-vulval morphogenesis in Caenorhabditis elegans. Genes Dev. *18*, 2380–2391.

Bruderer, R., Tatham, M.H., Plechanovova, A., Matic, I., Garg, A.K., and Hay, R.T. (2011). Purification and identification of endogenous polySUMO conjugates. EMBO Rep. *12*, 142–148.

Burrows, A.E., Sceurman, B.K., Kosinski, M.E., Richie, C.T., Sadler, P.L., Schumacher, J.M., and Golden, A. (2006). The C. elegans Myt1 ortholog is required for the proper timing of oocyte maturation. Development *133*, 697–709.

Cahoon, C.K., and Hawley, R.S. (2016). Regulating the construction and demolition of the synaptonemal complex. Nat. Struct. Mol. Biol. *23*, 369–377.

Cox, J., and Mann, M. (2008). MaxQuant enables high peptide identification rates, individualized p.p.b.-range mass accuracies and proteome-wide protein quantification. Nat. Biotechnol. *26*, 1367–1372.

Cox, J., Neuhauser, N., Michalski, A., Scheltema, R.A., Olsen, J.V., and Mann, M. (2011). Andromeda: a peptide search engine integrated into the MaxQuant environment. J. Proteome Res. *10*, 1794–1805.

Cubeñas-Potts, C., and Matunis, M.J. (2013). SUMO: a multifaceted modifier of chromatin structure and function. Dev. Cell *24*, 1–12.

Cubeñas-Potts, C., Srikumar, T., Lee, C., Osula, O., Subramonian, D., Zhang, X.D., Cotter, R.J., Raught, B., and Matunis, M.J. (2015). Identification of SUMO-2/3-modified proteins associated with mitotic chromosomes. Proteomics *15*, 763–772.

Dickinson, D.J., Pani, A.M., Heppert, J.K., Higgins, C.D., and Goldstein, B. (2015). Streamlined genome engineering with a self-excising drug selection cassette. Genetics *200*, 1035–1049.

Dumont, J., and Desai, A. (2012). Acentrosomal spindle assembly and chromosome segregation during oocyte meiosis. Trends Cell Biol. *22*, 241–249.

Dumont, J., Oegema, K., and Desai, A. (2010). A kinetochore-independent mechanism drives anaphase chromosome separation during acentrosomal meiosis. Nat. Cell Biol. *12*, 894–901.

Duro, E., and Marston, A.L. (2015). From equator to pole: splitting chromosomes in mitosis and meiosis. Genes Dev. *29*, 109–122.

Flotho, A., and Melchior, F. (2013). Sumoylation: a regulatory protein modification in health and disease. Annu. Rev. Biochem. *82*, 357–385.

Gareau, J.R., and Lima, C.D. (2010). The SUMO pathway: emerging mechanisms that shape specificity, conjugation and recognition. Nat. Rev. Mol. Cell Biol. *11*, 861–871.

Golden, A., Sadler, P.L., Wallenfang, M.R., Schumacher, J.M., Hamill, D.R., Bates, G., Bowerman, B., Seydoux, G., and Shakes, D.C. (2000). Metaphase to anaphase (mat) transition-defective mutants in Caenorhabditis elegans. J. Cell Biol. *151*, 1469–1482.

Hattersley, N., Cheerambathur, D., Moyle, M., Stefanutti, M., Richardson, A., Lee, K.Y., Dumont, J., Oegema, K., and Desai, A. (2016). A nucleoporin docks protein phosphatase 1 to direct meiotic chromosome segregation and nuclear assembly. Dev. Cell *38*, 463–477.

Hay, R.T. (2007). SUMO-specific proteases: a twist in the tail. Trends Cell Biol. *17*, 370–376.

Hillers, K.J., Jantsch, V., Martinez-Perez, E., and Yanowitz, J.L. (2015). Meiosis. WormBook. http://dx.doi.org/10.1895/wormbook.1.178.1.

Jentsch, S., and Psakhye, I. (2013). Control of nuclear activities by substrate-selective and protein-group SUMOylation. Annu. Rev. Genet. *47*, 167–186.

Kamath, R.S., and Ahringer, J. (2003). Genome-wide RNAi screening in Caenorhabditis elegans. Methods *30*, 313–321.

Kim, S.H., and Michael, W.M. (2008). Regulated proteolysis of DNA polymerase eta during the DNA-damage response in C. elegans. Mol. Cell *32*, 757–766.

Kitagawa, R., and Rose, A.M. (1999). Components of the spindle-assembly checkpoint are essential in Caenorhabditis elegans. Nat. Cell Biol. *1*, 514–521.

Monen, J., Maddox, P.S., Hyndman, F., Oegema, K., and Desai, A. (2005). Differential role of CENP-A in the segregation of holocentric C. elegans chromosomes during meiosis and mitosis. Nat. Cell Biol. *7*, 1248–1255.

Morawska, M., and Ulrich, H.D. (2013). An expanded tool kit for the auxin-inducible degron system in budding yeast. Yeast *30*, 341–351.

Muscat, C.C., Torre-Santiago, K.M., Tran, M.V., Powers, J.A., and Wignall, S.M. (2015). Kinetochore-independent chromosome segregation driven by lateral microtubule bundles. eLife *4*, e06462.

Ohkura, H. (2015). Meiosis: an overview of key differences from mitosis. Cold Spring Harb. Perspect. Biol. *7*, 7.

Pelisch, F., and Hay, R.T. (2016). Tools to study SUMO conjugation in caenorhabditis elegans. In SUMO: Methods and Protocols, S.M. Rodriguez, ed. (Springer New York), pp. 233–256.

Pelisch, F., Sonneville, R., Pourkarimi, E., Agostinho, A., Blow, J.J., Gartner, A., and Hay, R.T. (2014). Dynamic SUMO modification regulates mitotic chromosome assembly and cell cycle progression in Caenorhabditis elegans. Nat. Commun. *5*, 5485.

Powers, J., Rose, D.J., Saunders, A., Dunkelbarger, S., Strome, S., and Saxton, W.M. (2004). Loss of KLP-19 polar ejection force causes misorientation and missegregation of holocentric chromosomes. J. Cell Biol. *166*, 991–1001.

Psakhye, I., and Jentsch, S. (2012). Protein group modification and synergy in the SUMO pathway as exemplified in DNA repair. Cell *151*, 807–820.

Rogers, R.S., Inselman, A., Handel, M.A., and Matunis, M.J. (2004). SUMO modified proteins localize to the XY body of pachytene spermatocytes. Chromosoma *113*, 233–243.

Sapir, A., Tsur, A., Koorman, T., Ching, K., Mishra, P., Bardenheier, A., Podolsky, L., Bening-Abu-Shach, U., Boxem, M., Chou, T.F., et al. (2014). Controlled sumoylation of the mevalonate pathway enzyme HMGS-1 regulates metabolism during aging. Proc. Natl. Acad. Sci. USA *111*, E3880–E3889.

Schumacher, J.M., Golden, A., and Donovan, P.J. (1998). AIR-2: An Aurora/Ipl1-related protein kinase associated with chromosomes and midbody microtubules is required for polar body extrusion and cytokinesis in Caenorhabditis elegans embryos. J. Cell Biol. *143*, 1635–1646.

Severson, A.F., von Dassow, G., and Bowerman, B. (2016). Oocyte meiotic spindle assembly and function. Curr. Top. Dev. Biol. *116*, 65–98.

Tammsalu, T., Matic, I., Jaffray, E.G., Ibrahim, A.F., Tatham, M.H., and Hay, R.T. (2014). Proteome-wide identification of SUMO2 modification sites. Sci. Signal. *7*, rs2.

Tammsalu, T., Matic, I., Jaffray, E.G., Ibrahim, A.F., Tatham, M.H., and Hay, R.T. (2015). Proteome-wide identification of SUMO modification sites by mass spectrometry. Nat. Protoc. *10*, 1374–1388.

Tsur, A., Bening Abu-Shach, U., and Broday, L. (2015). ULP-2 SUMO protease regulates E-cadherin recruitment to adherens junctions. Dev. Cell *35*, 63–77.

Wang, Z.B., Ou, X.H., Tong, J.S., Li, S., Wei, L., Ouyang, Y.C., Hou, Y., Schatten, H., and Sun, Q.Y. (2010). The SUMO pathway functions in mouse oocyte maturation. Cell Cycle *9*, 2640–2646.

Wignall, S.M., and Villeneuve, A.M. (2009). Lateral microtubule bundles promote chromosome alignment during acentrosomal oocyte meiosis. Nat. Cell Biol. *11*, 839–844.

Yuan, Y.F., Zhai, R., Liu, X.M., Khan, H.A., Zhen, Y.H., and Huo, L.J. (2014). SUMO-1 plays crucial roles for spindle organization, chromosome congression, and chromosome segregation during mouse oocyte meiotic maturation. Mol. Reprod. Dev. *81*, 712–724.

Zhang, H., Smolen, G.A., Palmer, R., Christoforou, A., van den Heuvel, S., and Haber, D.A. (2004). SUMO modification is required for in vivo Hox gene regulation by the Caenorhabditis elegans Polycomb group protein SOP-2. Nat. Genet. *36*, 507–511.

Zhang, X.D., Goeres, J., Zhang, H., Yen, T.J., Porter, A.C., and Matunis, M.J. (2008). SUMO-2/3 modification and binding regulate the association of CENP-E with kinetochores and progression through mitosis. Mol. Cell *29*, 729–741.

Zhang, L., Ward, J.D., Cheng, Z., and Dernburg, A.F. (2015). The auxin-inducible degradation (AID) system enables versatile conditional protein depletion in C. elegans. Development *142*, 4374–4384.

Molecular Cell, Volume *65*

Supplemental Information

A SUMO-Dependent Protein Network Regulates Chromosome Congression during Oocyte Meiosis

Federico Pelisch, Triin Tammsalu, Bin Wang, Ellis G. Jaffray, Anton Gartner, and Ronald T. Hay

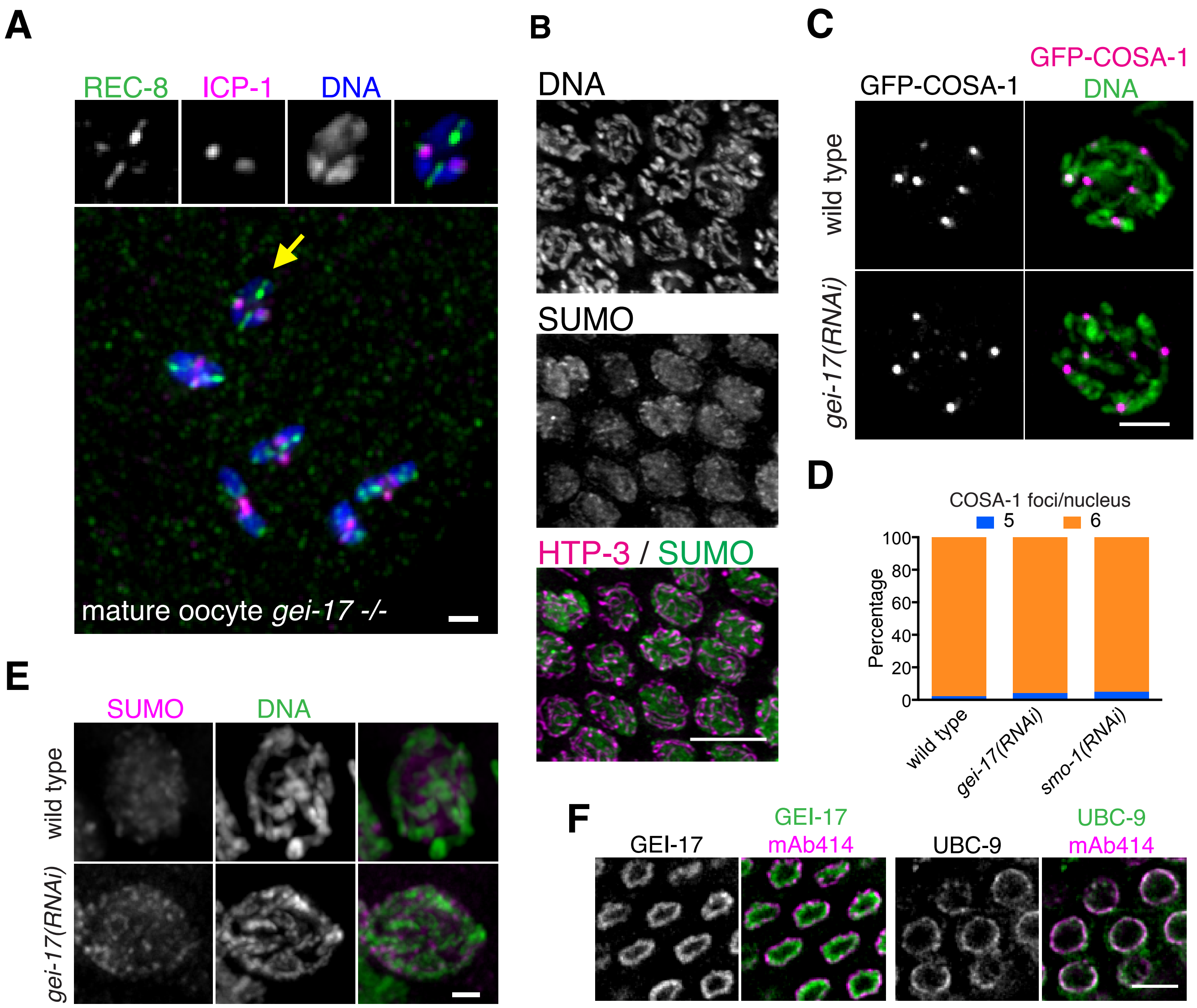
A
REC-8
ICP-1
DNA
mature oocyte gei-17 -/-
B
DNA
SUMO
HTP-3 / SUMO
C
GFP-COSA-1
GFP-COSA-1
DNA
wild type
gei-17(RNAi)
D
COSA-1 foci/nucleus
5
6
Percentage
100
80
60
40
20
0
wild type
gei-17(RNAi)
smo-1(RNAi)
E
SUMO
DNA
wild type
gei-17(RNAi)
F
GEI-17
GEI-17
mAb414
UBC-9
UBC-9
mAb414

Pelisch et al. Figure S2

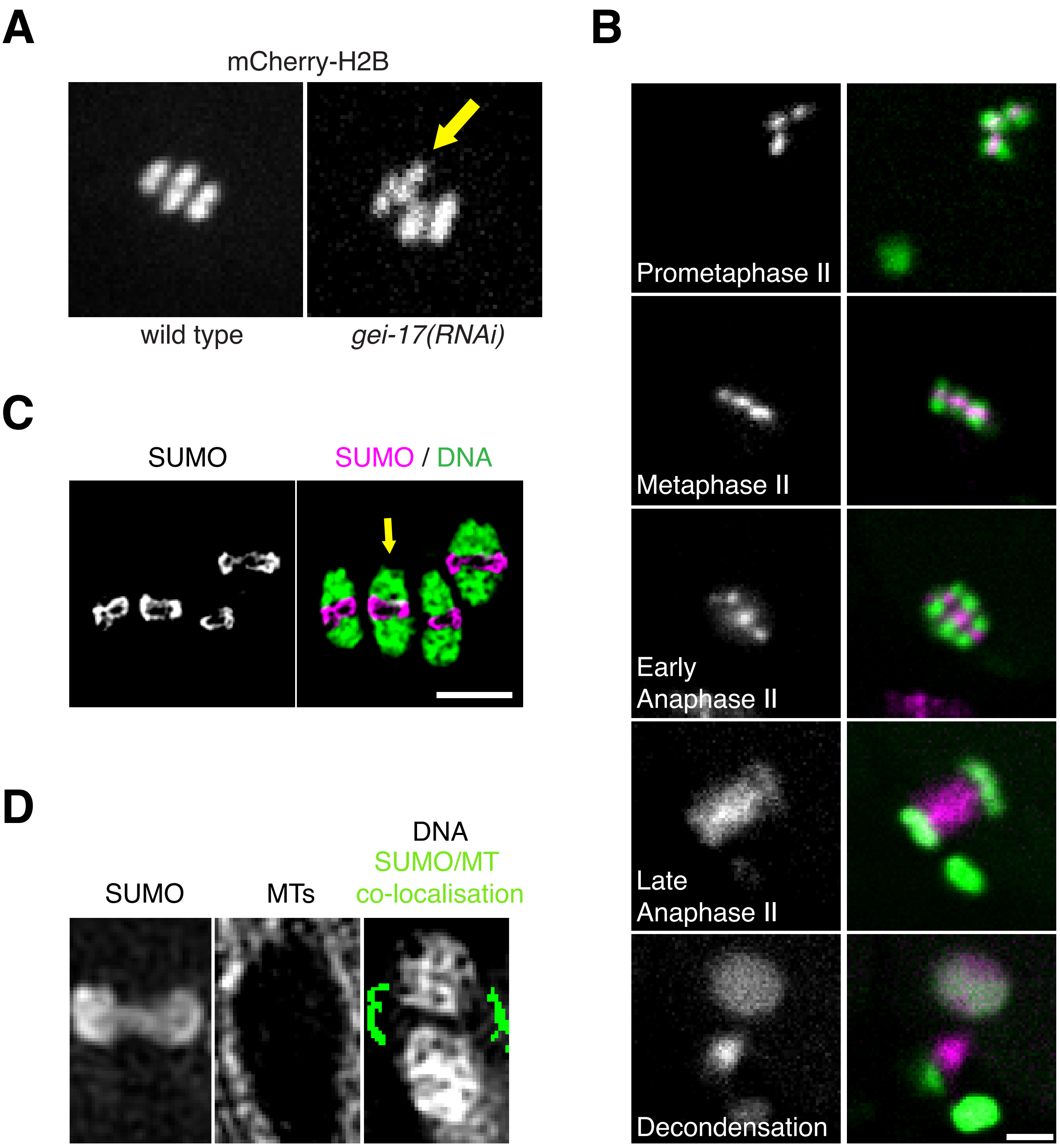

Pelisch et al. Figure S3

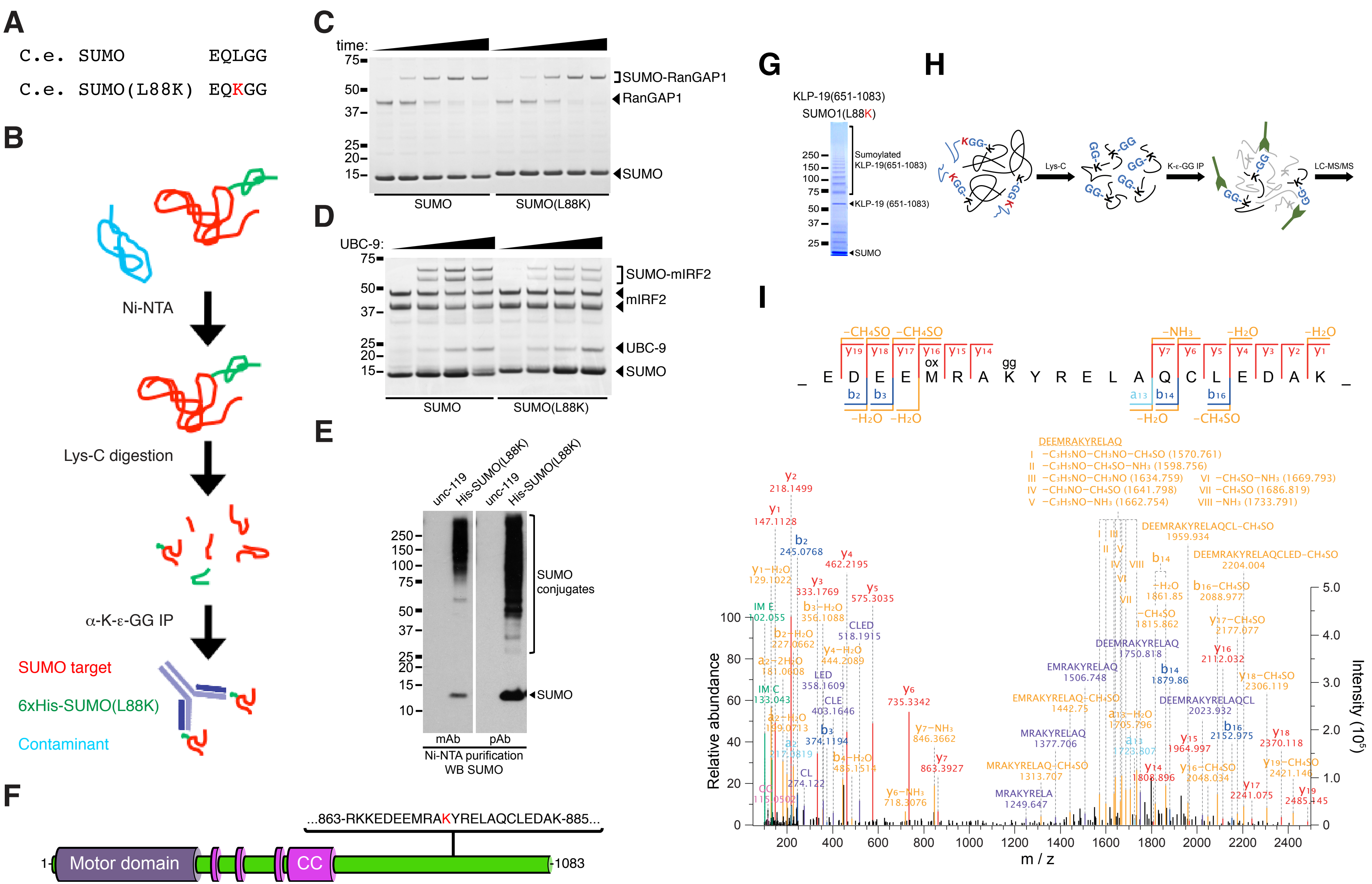

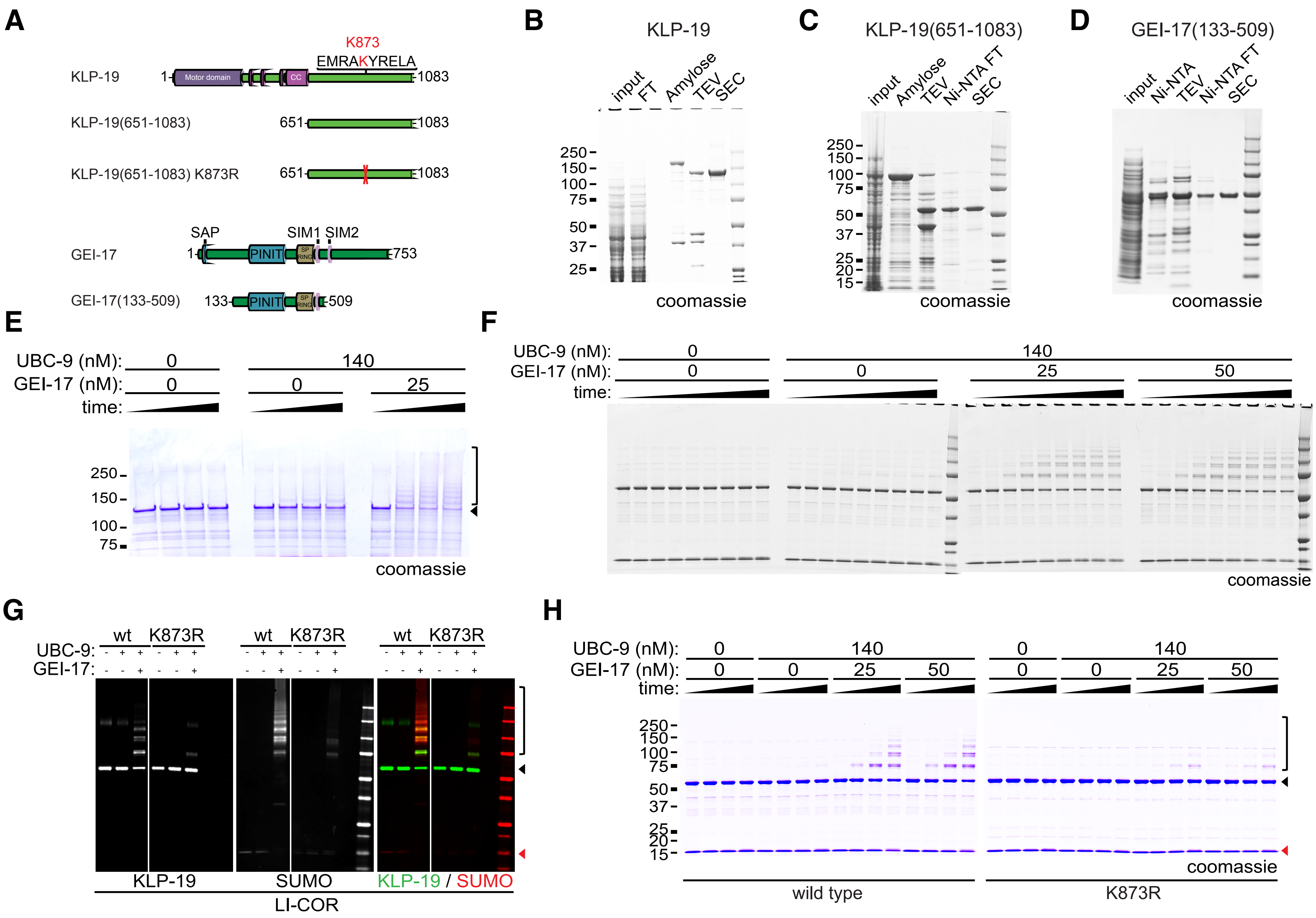
A
K873
EMRAKYRELA
KLP-19
1
Motor domain
CC
1083
KLP-19(651-1083)
651
1083
KLP-19(651-1083) K873R
651
1083
SAP
SIM1 SIM2
GEI-17
1
PINIT
SP RING
753
GEI-17(133-509)
133
PINIT
SP RING
509
B
KLP-19
input
FT
Amylose
TEV
SEC
250
150
100
75
50
37
25
coomassie
C
KLP-19(651-1083)
input
Amylose
TEV
Ni-NTA FT
SEC
250
150
100
75
50
37
25
20
15
coomassie
D
GEI-17(133-509)
input
Ni-NTA
TEV
Ni-NTA FT
SEC
coomassie
E
UBC-9 (nM):
0
140
GEI-17 (nM):
0
0
25
time:
250
150
100
75
coomassie
F
UBC-9 (nM):
0
140
GEI-17 (nM):
0
0
25
50
time:
coomassie
G
wt
K873R
UBC-9:
GEI-17:
KLP-19
SUMO
KLP-19 / SUMO
LI-COR
H
UBC-9 (nM):
0
140
0
140
GEI-17 (nM):
0
0
25
50
0
0
25
50
time:
250
150
100
75
50
37
25
20
15
coomassie
wild type
K873R

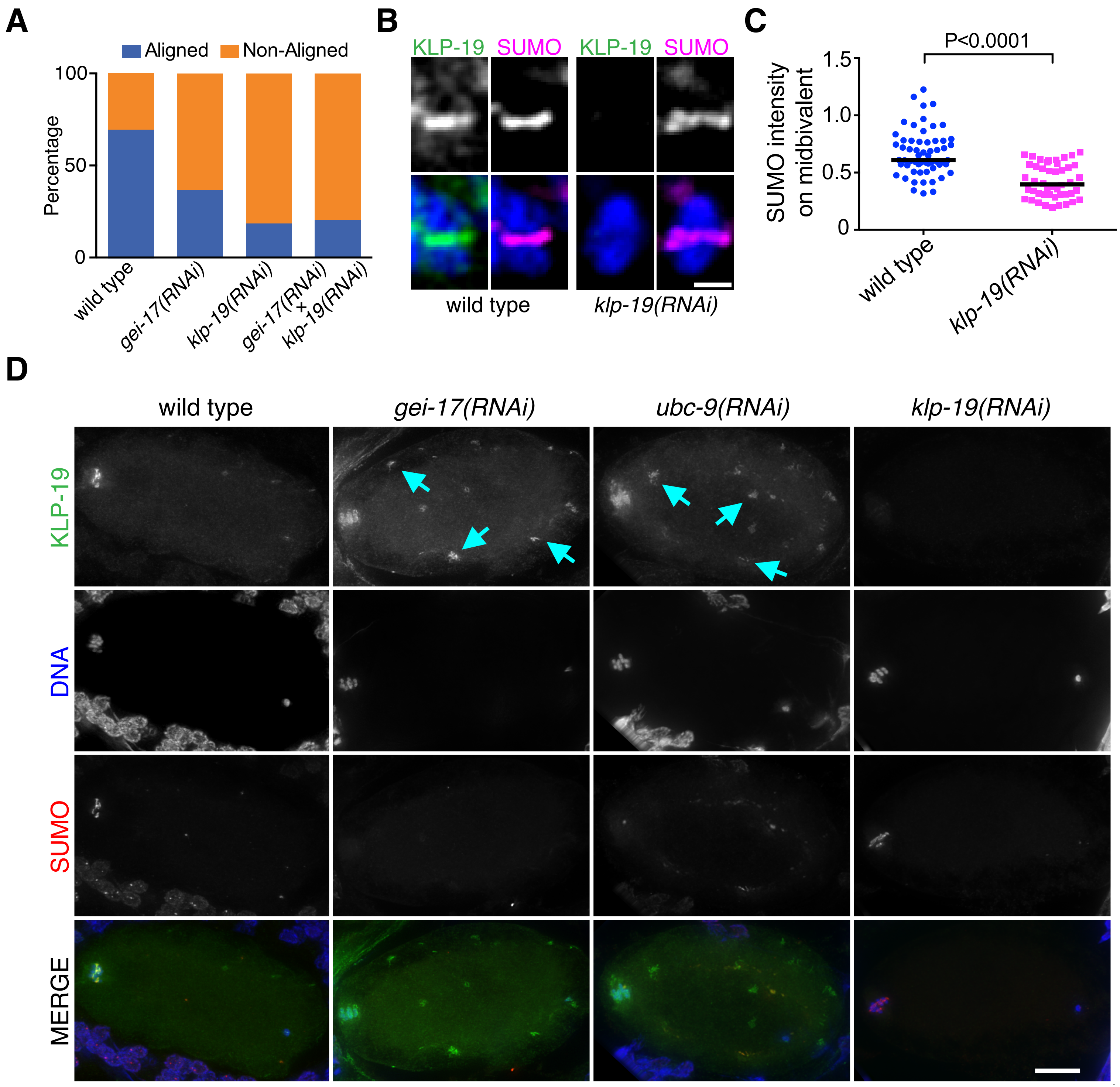
A
Aligned
Non-Aligned
Percentage
100
50
0
wild type
gei-17(RNAi)
klp-19(RNAi)
gei-17(RNAi) x klp-19(RNAi)
B
KLP-19
SUMO
KLP-19
SUMO
wild type
klp-19(RNAi)
C
P<0.0001
SUMO intensity on midbivalent
1.5
1.0
0.5
0
wild type
klp-19(RNAi)
D
wild type
gei-17(RNAi)
ubc-9(RNAi)
klp-19(RNAi)
KLP-19
DNA
SUMO
MERGE

Pelisch et al. Figure S6

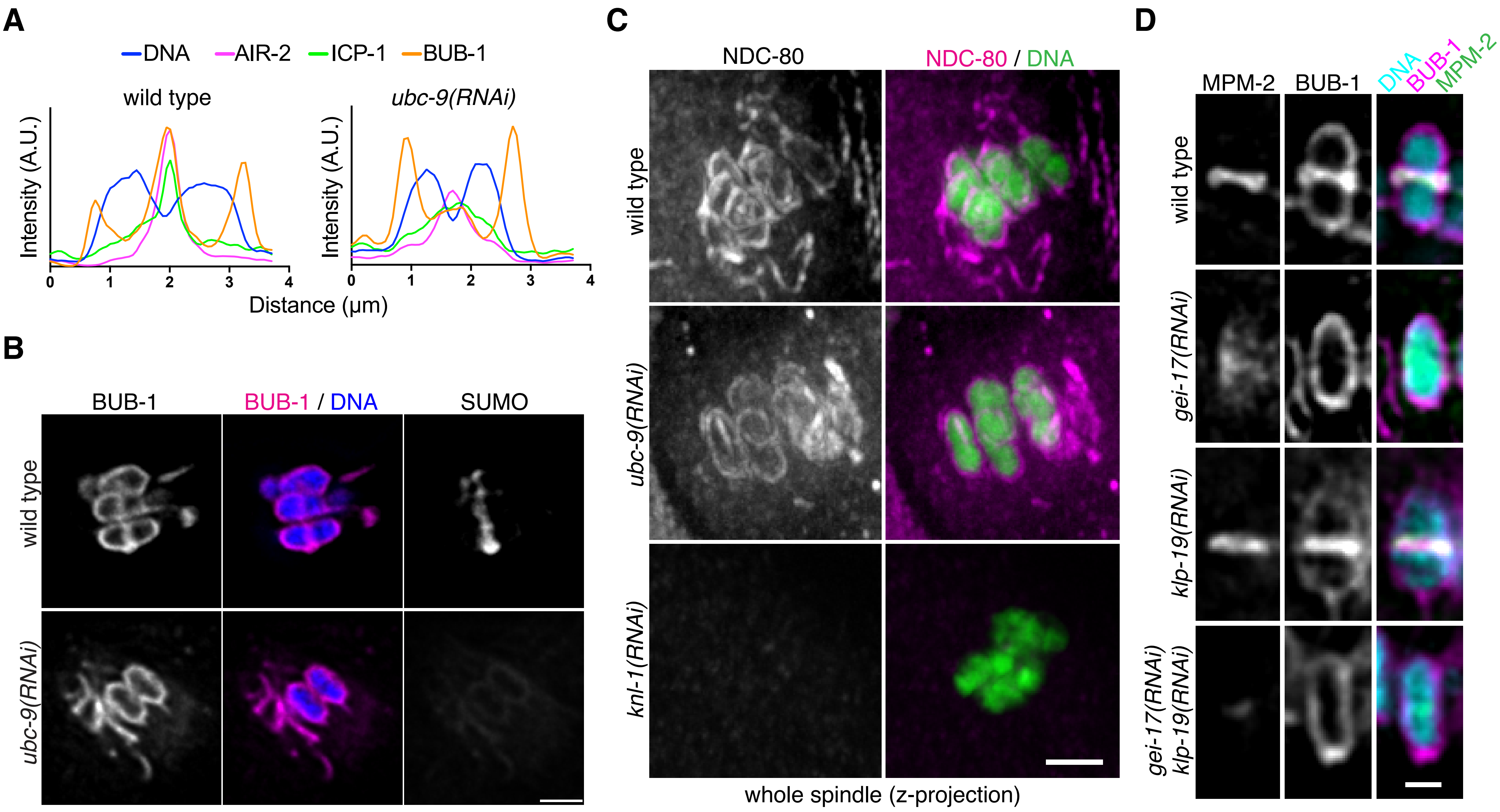

Pelisch et al. Figure S7

SMO-1

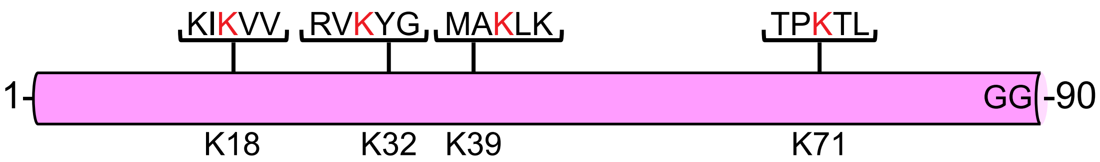


```
1-    MADDAAQAGD   NAEYIKIKVV   GQDSNEVHFR   VKYGTSMAKL
41-   KKSYADRTGV   AVNSLRFLFD   GRRINDDDTP   KTLEMEDDDV
81-   IEVYQEQLGG   F
```

GEI-17 (isoform f)

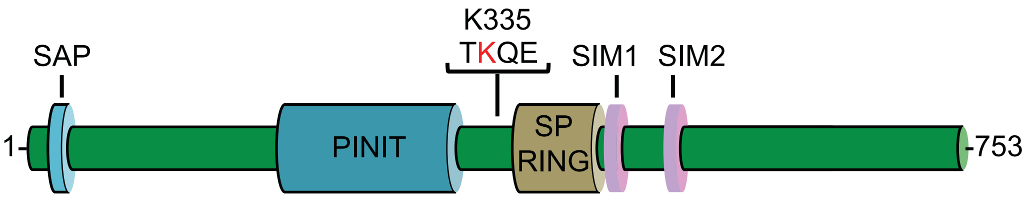


```
133-   QQMMASHHSH    LQQQHHPSTP    KKMYADNFEP    LPLPFYDVIS
173-   VLLKPVELHS    SDSPTLKQTK    QLQFPFLLTA    EHISKISYRA
213-   DVTPLPRYEL    QLRFFNLTEP    VQGPQKDDFP    LNCYARVDDS
253-   VVQLPNVIPT    NKTNAEPKRP    SRPVNITSNM    NRYKKEHTVA
293-   VEWLADKRVW    AAGVYFVHRV    NSDILFKRLN    QNVSRHRSLE
333-   VTKQEVIKKL    SGGEDDIAMD    RLNISLLDPL    CKTRMTTPSR
373-   CQDCTHLQCF    DLLSYLMMNE    KKPTWQCPVC    SSNCPYDRLI
413-   VDDYFLDMLA    KVDKNTTEVE    LKEDGSYDVI    KEEAFCISDD
453-   DDDDVVPATV    NGTASCSSTN    GNGLANEAAK    KKPADDDIIT
493-   LSDDDDEELN    RGIMNSL
```

KLP-19

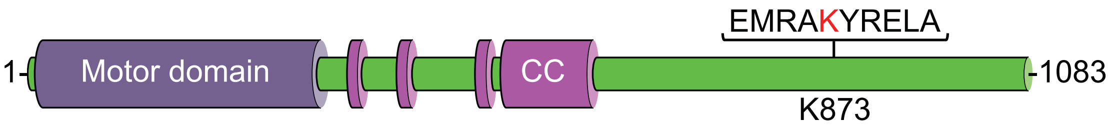


```
651-   VAIQKRMTDQ   KLTVLQMRLT   EANRANKTLR   ELNLKRANRK
691-   SSPTNASALQ   NMIEEELEHE   MCAQRSHWLC   EDLRRQRHDL
731-   MQNINTVESM   KFEGGKRRRI   SASADPNVSV   VIEGEEEFEV
771-   KRQKELTFLR   ASLETLNEEI   KDSLRNETIA   GNEERANSRW
811-   EKVPAEMRPA   FEAVYAQAVA   HIRKEIELEF   KLARTKSEFT
851-   AKIASKASHE   EKRKKEDEEM   RAKYRELAQC   LEDAKSGLHE
891-   KIAFLLCLIK   ENRVDENAIQ   QFESLKNQFC   DVEQKVKKAS
931-   RRKTTNFMGG   LTPKPELQRN   ERARRAVKYY   GNVVNSEDVT
971-   MDDSRHQKRK   DHSLLAVEMN   RTTDDNVKRR   VAMSPIKCDD
1011-  DTRLTEEDED   IENEAMNNAT   FVKDSFNSAT   IVLDDSQPSP
1051-  SNSTFVIGAA   PTSEADGVPP   IKRKSRRTDL   GPL
```

Supplemental Figure Legends

Figure S1 (related to Figure 1). *C. elegans* sumoylation pathway in pachytene.
A. An oocyte from a *gei-17 -/-* was stained with ICP-1 in magenta, REC-8 in green, and DNA in blue. The bottom panel corresponds to a zoomed image of the bivalent marked with a yellow arrow. Scale bar, 1 µm.
B. Nuclei in the pachytene stage of meiosis showing SUMO localisation in green and HTP-3 in magenta. Scale bar, 5 µm.
C. GFP-COSA-1 foci in late pachytene were counted from dissected gonads of wild type and *gei-17(RNAi)* worms. GFP-COSA-1 is shown in magenta and DNA in green. Scale bar, 2 µm.
D. Quantitation of COSA-1 foci in late pachytene in wild type, *gei-17(RNAi)*, or *smo-1(RNAi)* worms.
E. SUMO localisation pattern in pachytene nuclei was assessed in control ('wild type') and GEI-17-depleted worms ['*gei-17(RNAi)*']. Scale bar, 1 µm.
F. GEI-17 and UBC-9 localisation in pachytene nuclei was assessed by immunofluorescence. The nuclear pore complex marker mAb414 was used to co-stain the nuclear envelope. Scale bar, 5 µm.

Figure S2 (related to Figure 2).
A. Metaphase I images of oocytes expressing mCherry-H2B, either from wild type or *gei-17(RNAi)* worms. The yellow arrow points to one misaligned bivalent.
B. Meiosis II in worms expressing mCherry-SUMO/GFP-H2B was followed by in utero time lapse. Still images of the different stages are shown with SUMO coloured in magenta and H2B in green. Scale bar, 2 µm.
C. The image corresponds to a bigger field of view to that showed in Figure 2C. The bivalent shown in the Figure 2C is indicated by a yellow arrow. Scale bar, 2 µm.
D. 3D-SIM image of a meiosis I spindle region showing staining for SUMO, microtubules ('MTs') and their co-localisation, highlighted in green in the panel on the right.

Figure S3 (related to Figure 3). Mass spectrometry-based SUMO site identification strategy, setup, and results.
A. The C-terminus of the nematode processed SUMO is indicated, with the Leu to Lys substitution highlighted in red.
B. A schematic of the protocol used for the identification of sumoylation sites in vivo. Briefly, worms expressing 6xHis-SUMO(L88K) are lysed and a denaturing Ni-NTA purification is performed. After Lys-C digestion, GG-K-modified peptides are immunoprecipitated using an anti-K-e-GG specific antibody, before analysis by mass spectrometry.
C. A time course analysis of RanGAP1 sumoylation using wild type and L88K mutant SUMO. Proteins were visualised by coomassie staining.
D. mIRF2 sumoylation with wild type and L88K mutant SUMO was analysed using increasing UBC-9 concentrations.
E. Hexahistidine-tagged SUMO(L88K) is conjugated to substrates in vivo. Worms expressing 6xHis-SUMO(L88K) were lysed denaturing Ni-NTA chromatography was performed. Eluted material was analysed by western blotting using a monoclonal ('mAb') or a polyclonal ('pAb') anti-SUMO antibodies.
F. Diagram of KLP-19 highlighting the motor domain, the predicted coiled-coil domains (in pink, 'CC'), and the peptide identified as SUMO modified in vivo.
G. Recombinant KLP-19(651-1083) was SUMO modified in vitro and an aliquot of the reaction was run on an SDS-PAGE and analysed by coomassie staining.
H. Diagram of the workflow for identifying SUMO modification sites from in vitro reactions.
I. KLP-19 is sumoylated in vitro. A representative annotated MS/MS spectrum of diGly remnant-containing peptide of KLP-19 (Lysine 873 is modified). Fragment ions extending from the N-terminus of the peptide are named as b- (in dark blue) or a-ions (in light blue), and the individual a-b ion pairs have a mass difference of a carbonyl group (C=O, 27.995 Da). C-terminal y-ions of the peptide are illustrated in red. Peptide internal fragments, ions with a loss of neutral molecule(s) or immonium ions are reported in purple, yellow or green, respectively. Ions diagnostic for diGly modification are shown in pink. This annotated MS/MS spectrum has an Andromeda score of 104.86 with a posterior error probability of 5.63 x 10-9.

Figure S4 (related to Figure 3). Detailed in vitro analysis of KLP-19 sumoylation.

A. Schematic of the different KLP-19 and GEI-17 proteins and fragments used for in vitro SUMO modification assays.
B. Summary of the purification process of full-length KLP-19. The protein was expressed as a 6xHis-MBP fusion with a TEV protease site between the tag and KLP-19, and purified using amylose beads. After TEV cleavage, the protein was purified by size exclusion chromatography using a Superose 6 column. Input, soluble lysate; FT, flow-through of the amylose column; Amylose, maltose-eluted material; TEV, sample after TEV cleavage; SEC, sample after size exclusion chromatography.
C. Summary of the purification process of KLP-19(651-1083). The protein was expressed as a 6xHis-MBP fusion with a TEV protease site between the tag and KLP-19, and purified using amylose beads. After TEV cleavage, the 6xHis-MBP tag and the 6xHis-TEV were removed using a Ni-NTA column. The flow through (FT, containing KLP-19(651-1083)) was further purified by size exclusion chromatography using a Superdex 200 column. Input, soluble lysate; Amylose, maltose-eluted material; TEV, sample after TEV cleavage; Ni-NTA FT, Ni-NTA column flow-through of TEV treated sample; SEC, sample after size exclusion chromatography.
D. Summary of the purification process of GEI-17(133-509). The protein was expressed as a 6xHis fusion with a TEV protease site between the tag and GEI-17, and purified using Ni-NTA beads. After TEV cleavage, the 6xHis tag and the 6xHis-TEV were removed using a Ni-NTA column. The flow through (FT, containing untagged GEI-17) was further purified by size exclusion chromatography using a Superdex 200 column. Input, soluble lysate; Ni-NTA, imidazole-eluted material; TEV, sample after TEV cleavage; Ni-NTA FT, Ni-NTA column flow-through of TEV treated sample; SEC, sample after size exclusion chromatography.
E. KLP-19 was subject to in vitro sumoylation. Reactions contained 1 μM KLP-19, 20 μM SUMO, and the indicated amounts of UBC-9 and GEI-17 and were incubated for 0, 30, 60, and 120 minutes.
F. KLP-19(651-1083) was subject to in vitro sumoylation. Reactions contained 6 μM KLP-19, 20 μM SUMO, and the indicated amounts of UBC-9 and GEI-17 and were incubated for 0, 15, 30, 45, 60, 75, 90, 105, and 120 minutes. Red arrowhead, free SUMO. Black arrowhead, unmodified KLP-19. Square bracket, SUMO-modified KLP-19.
G. KLP-19(651-1083) and KLP-19(651-1083) K873R were subject to in vitro sumoylation. Reactions contained 3 μM KLP-19, 20 μM SUMO, 140 nM UBC-9, and 12.5 nM GEI-17. Reactions were incubated for 60 minutes and analysed by two-color western blotting using a LI-COR system. Red arrowhead, free SUMO. Black arrowhead, unmodified KLP-19. Square bracket, SUMO-modified KLP-19. Note that this is the same blot shown in Figure 3F, showing the individual channels in addition to the merged image.
H. KLP-19(651-1083) and KLP-19(651-1083) K873R were subject to in vitro sumoylation. Reactions contained 6 μM KLP-19, 20 μM SUMO, and the indicated amounts of UBC-9 and GEI-17 and were incubated for 0, 15, 30, and 60 minutes. Red arrowhead, free SUMO. Black arrowhead, unmodified KLP-19. Square bracket, SUMO-modified KLP-19.

Figure S5 (related to Figure 3).
A. Spindles were characterised as either ‘aligned’ or ‘non-aligned’ (at least one chromosome away from the metaphase plate) and results from wild type, *gei-17(RNAi)*, *klp-19(RNAi)*, and *gei-17(RNAi)* + *klp-19(RNAi)* oocytes are shown as % of total.
B. Metaphase I-arrested wild type or *klp-19(RNAi)* oocytes were stained for KLP-19, SUMO, and DNA as indicated.
C. Quantitation of the SUMO signal form the experiment shown in B. Results were analysed by a Mann-Whitney test and black lines indicate the median.
D. Whole oocyte corresponding to the spindle images shown in Figure 3I. The cyan arrows point to the cortical linear elements.

Figure S6 (related to Figure 4). RC but not kinetochores are affected in the absence of sumoylation.
A. Representative intensity profiles of single bivalents from wild type or *ubc-9(RNAi)* oocytes that stained with alexa-labelled AIR-2, ICP-1, and BUB-1 antibodies.
B. Single z slice from wild type or *ubc-9(RNAi)* oocytes stained for BUB-1 (magenta) and DNA (blue). Scale bar, 2 μm.
C. Localisation of the kinetochore component NDC-80 was analysed in the presence of control RNAi (‘wild type’), *ubc-9(RNAi)*, or *knl-1(RNAi)*, a known regulator of kinetochore assembly. The images correspond to whole spindle, z-projections of the single bivalents shown in Figure 4C. Scale bar, 2 μm.

D. MPM-2 reactive antigens and BUB-1 localisation were analysed in control ('wild type'), *gei-17(RNAi)*, *klp-19(RNAi)*, *and gei-17(RNAi) + klp-19(RNAi)* in metaphase I-arrested oocytes. Scale bar, 1 μm.

Figure S7 (Related to Figure 3, see main text). SUMO conjugation sites identified in SUMO, GEI-17, and KLP-19.
Lysines found to be SUMO modified in GEI-17, KLP-19, or SUMO itself during in vitro assays.

Supplemental Experimental Procedures

Worms. *C. elegans* strains were maintained according to standard procedures (Brenner, 1974). Where indicated, transgenic worms were generated by particle bombardment (Praitis et al., 2001). To generate the GFP-tagged fusion protein, the respective full-length cDNAs were amplified from N2 worms and cloned into PIE-1 regulatory element (Wallenfang and Seydoux, 2000) in a pIC26 vector (Cheeseman and Desai, 2005). The complete set of strains used in the present paper is listed below. Please note that plasmid names in FGP strains have been corrected and differ from the ones used in previous papers (Pelisch and Hay, 2016; Pelisch et al., 2014).

Strain	Genotype	
FGP1	*fgpIs20[pFGP79; Ppie-1 mCherry::smo-1(GG) unc-119(+)], unc-119 (ed3)*	FGP Lab
FGP2	*fgpIs21[pFGP80; Ppie-1 mCherry::smo-1(GA) unc-119(+)], unc-119 (ed3)*	FGP Lab
FGP3	*fgpIs23[pFGP78; pie-1/GFP-TEV-S-Tag::smo-1(GG) unc-119(+)], unc-119 (ed3)*	FGP Lab
FGP4	*fgpIs24[pFGP77; pie-1/GFP-TEV-S-Tag::smo-1(GA) unc-119(+)], unc-119 (ed3)*	FGP Lab
FGP8	*ruIs32 [pie-1::GFP::H2B + unc-119(+)], fgpIs20[pFGP79; Ppie-1 mCherry::smo-1(GG) unc-119(+)], unc-119 (ed3)*	FGP Lab
FGP17	*fgpIs37[pFGP60; Psmo-1::6xHis::smo-1(L88K,GG)::smo-1 3'UTR unc-119(+)], unc-119 (ed3)*	this study
FGP9	*fgpIs23[pFGP78; pie-1/GFP-TEV-S-Tag::smo-1(GG) unc-119(+)], unc-119 (ed3); ltIs37 [pAA64; pie-1p::mCherry::his-58 + unc-119(+)]*	this study
FGP10	*fgpIs24[pFGP77; pie-1/GFP-TEV-S-Tag::smo-1(GA) unc-119(+)], unc-119 (ed3); ltIs37 [pAA64; pie-1p::mCherry::his-58 + unc-119(+)]*	this study
EU630	*air-2(or207) I*	CGC
HY604	*mat-1(ye121) I*	CGC
GG48	*emb-27(g48) II*	CGC
OD987	*ltSi264[pOD1949/pTK011; Pbub-1::Bub1 reencoded::mCherry; cb-unc-119(+)]II;unc-119(ed3)III*	OD Lab
VC1915	*klp-18(ok2519) IV/nT1 [qIs51] (IV;V)*	CGC
OD987	*ltSi264[pOD1949/pTK011; Pbub-1::Bub1 reencoded::mCherry; cb-unc-119(+)]II;unc-119(ed3)III*	OD lab
FGP24	*ltSi264[pOD1949/pTK011; Pbub-1::Bub1 reencoded::mCherry; cb-unc-119(+)]II;unc-119(ed3)III; ruIs32 [pie-1::GFP::H2B + unc-119(+)], unc-119 (ed3)*	this study
FGP26	*ltSi264[pOD1949/pTK011; Pbub-1::Bub1 reencoded::mCherry; cb-unc-119(+)]II;unc-119(ed3)III; fgpIs23[pFGP78; pie-1/GFP-TEV-S-Tag::smo-1(GG) unc-119(+)], unc-119 (ed3)*	this study
FGP28	*gei-17(fgp1[GFP::FLAG::degron::loxP::gei-17])*	this study
FGP29	*gei-17(fgp1[GFP::FLAG::degron::loxP::gei-17]); ieSi38[Psun-1::TIR1::mRuby::sun-1 3'UTR,cb-unc-119(+)] IV*	this study
FGP30	*gei-17(fgp1[GFP::FLAG::degron::loxP::gei-17]); ltIs37 [pAA64; pie-1p::mCherry::his-58 + unc-119(+)]*	this study
FGP36	*klp-19(fgp3[klp-19 A2618>G, A2623>C, A2625>T, T2629>C, G2631>T, T2634>C, A2637>G, T2640>C, G2643>C, A2646>G])*	this study

Generation of GFP-FLAG-degron-GEI-17. GEI-17 fused to GFP-FLAG-degron was generated by CRISPR exactly as described (Dickinson et al., 2015), using a pDD282-based plasmid as a repair template generated by Gibson assembly (NEBuilder HiFi DNA Assembly Master Mix, New England Biolabs). We targeted the N-terminus of GEI-17 as this is the same in all the GEI-17 isoforms. Two gRNAs were used (5'-GTCGTTTCGAGACACAGCGG-3' and 5'-GAGACACAGCGGAGGATCGG-3'). Both sequences target PAM motifs upstream of the initial ATG codon, thus in order to avoid Cas9 cleavage on the repaired locus, we made a G>A mutation in the –15 position, relative to the ATG within the *gei-17* promoter region. Injections and screening were performed by Knudra Transgenics. The degron sequence consisted of the 44-aa fragment of the Arabidopsis thaliana IAA17 protein (Morawska and Ulrich, 2013; Zhang et al., 2015). Sequences of all plasmids are available upon request.

Generation of KLP-19 K873R. Point mutation in the endogenous *klp-19* locus was achieved through ALT-R CRISPR-Cas9 system (IDT), with a protocol provided by Simone Köhler and Abby Dernburg. Cas9 protein was produced in-house or purchased from PNA Bio. Cas9 (30 μM) was incubated for 5 min with a 1:1 mixture of crRNA:tracrRNA (30 μM). The injection mix also contained the repair template (100 ng/μl), and marker plasmids pCFJ90 (2.5 ng/μl) and pCFJ104 (5 ng/μl). gRNA sequence was 5'-CAGAGAGUUGGCUCAAUGUC-3', and the repair template sequence (ssDNA, Ultramer oligo, IDT) was the following:

```
aagttggcgcgaacaaagtccgagttcacggcgaaaattgcttcgaaagccagccacgaggagaagagaaagaaggagga
tgaggagatgagagcaaGatacCgTgagCtTgcCcaGtgCctCgaGgatgccaagtctggattgcatgaaaagatcgctt
tcctcctgtgcttgatcaaggaaaatcgggtc
```

G: point mutation to generate an Arg in position 873.

X: Synonymous mutations to avoid Cas9 cleavage of repaired sequence.

X: Synonymous mutations to generate an Xho I site for screening (in Leu 881 and Asp 882).

Primers klp-19screenfwd: 5'-gagacgtatatctgcgag-3' and klp-19screenrev: 5'-cctcccatgaagtttgtc-3' were used for screening and sequencing.

Auxin treatment. Auxin (IAA, Sigma-Aldrich I5148) was used at 1 mM final concentration in standard NGM plates, unless otherwise noted. All plates for auxin treatment were prepared, allowed to dry for 2 days and a lawn of concentrated OP50 bacteria was seeded, as auxin inhibits bacterial growth. For auxin treatment, worms were placed on auxin-containing plates for the indicated time.

RNAi. Bacterial (HT115) clones expressing dsRNA for feeding strains were obtained from a commercial library (Kamath and Ahringer, 2003). Bacteria were grown at 37°C to OD_{600} = 1 and then spread on nematode growth media plates supplemented with 1 mM IPTG and incubated for 24 h at 20°C. For most experiments, L4 worms were then added to plates and fed for 24–32 h at 20°C, before analysis. For experiments involving APC alleles, worms were shifted to 25°C for 5-7 h before fixing or imaging. For experiments with monopolar spindles, L1 *klp-18(ok2519)* worms were fed with bacteria expressing *emb-30(RNAi)* for 3 days at 15°C and then changed to plates containing *emb-30(RNAi)* plus control or SUMO pathway RNAi (1:1 mix) for a further 2 days at 15°C.

Peptide injection. The experiment in Figure 7J was performed by injecting either a SIM consensus peptide (VDVIDLTIEEDE) (Bruderer et al., 2011) or a control peptide (YGSFQDSVSMREDC), both at 700 μM, into the gonads of *emb-27* young adults. After 24 recovery at 15°C, worms were shifted to 25°C for 5 h to induce metaphase I arrest. Then, worms were processed for embryo immunofluorescence using FITC-labelled anti-α-tubulin (DM1A, Abcam, ab64503), Alexa 594-labelled anti-KLP-19, and Alexa 647-labelled anti-BUB-1 antibodies.

Antibodies. Antibodies against SMO-1, GEI-17, and UBC-9 were reported previously (Pelisch et al., 2014). An anti-SMO-1 monoclonal antibody is available from the Developmental Studies Hybridoma Bank (DSHB, http://dshb.biology.uiowa.edu/SUMO-6F2). The All sera were adsorbed with HT115 bacterial lysate and affinity purified with the antigenic peptide/protein (except for KLP-19, see below). AIR-2 (CQKIEKEASLRNH) and ICP-1 (VKVKKRGSSAVWK) peptide antibodies were produced and affinity purified by Moravian Biotech using previously described peptides (Burrows et al., 2006; Schumacher et al., 1998). Anti-KLP-19 serum (Powers et al., 2004) was subject to protein A purification before use. The resulting antibody was used at 2 μg/ml. A monoclonal antibody recognising alpha-tubulin was used at 2.5 μg/ml (clone DM1A, Sigma). Anti-BUB-1 (Oegema et al., 2001) and anti SEP-1 (Bembenek et al., 2007) antibodies were used at 0.5 μg/ml. A complete list of the antibodies used in the present study is included below:

antibody	species	WB	IF
UBC-9 (full length)	Sheep	1 μg/ml	10 μg/ml
GEI-17 (aa 133-509)	Rabbit	1 μg/ml	10 μg/ml
ICP-1 (aa 591-604)	Rabbit	N/A	1 μg/ml
AIR-2 (aa 294-305)	Rabbit	1 μg/ml	1 μg/ml
pH3 (Merck-Millipore, #06-570)	Rabbit	N/A	0.2 μg/ml
MPM-2 (Thermo 0.T.181)	Mouse	N/A	2 μg/ml
NDC-80 (Novus Biologicals, #42000002)	Rabbit	N/A	1 μg/ml
SMO-1 (full length)	Mouse	1 μg/ml	1 μg/ml
SMO-1 (full length)	Sheep	1 μg/ml	1 μg/ml
Tubulin (DM1A, Sigma-Aldrich #T6199)	Mouse	N/A	2.5 μg/ml
Tubulin (DM1A, FITC, Abcam #ab64503)	Mouse		
SYP-1	Rabbit	N/A	1/100
REC-8	Mouse	N/A	1/100
HTP-3 (Monique Zetka)	Rabbit	N/A	1/100
HCP-6 (Barbara Meyer)	Rabbit	N/A	5 μg/ml
KLP-19 (Susan Stromme)	Rabbit	1 μg/ml	2 μg/ml
BUB-1 (Tony Hyman)	Rabbit	N/A	0.5 μg/ml
SEP-1 (Andy Golden)	Rabbit	N/A	0.5 μg/ml

Primary antibody labelling. For all experiments involving fluorescence intensity measurements, antibodies were labelled with Alexa fluorophores. The APEX Alexa Fluor labelling kits (Thermo Scientific) were used and antibodies

were labelled with Alexa 488, Alexa 594, and Alexa 647, following the manufacturer's indications. Antibodies were buffer exchanged to PBS using Zeba™ Spin Desalting Columns (Thermo Scientific) and were stored in small aliquots at -20°C in PBS containing 10% glycerol. Labelled antibodies were used at 1-5 μg/ml for immunofluorescence.

In utero embryo live imaging. For live imaging, GFP- or mCherry-expressing worms were picked into a solution of tricaine (0.1%) and tetramisole (0.01%), and incubated for 20 min. Worms were then pipetted onto a 4% agar pad, covered with a coverslip, and imaged with a spinning-disk confocal microscope (MAG Biosystems) mounted on a microscope (IX81; Olympus) with a 100×/1.45 Plan Apochromat oil immersion lens (Olympus), a camera (Cascade II; Photometrics), spinning-disk head (CSU-X1; Yokogawa Electric Corporation), and MetaMorph software (Molecular Devices). Image stacks were obtained at 1.5 μm z-steps and 15- or 30-s intervals using 2x2 binning.

Immunostaining. Worms were placed on 4 μl of M9 worm buffer in a poly-D-lysine (Sigma, P1024)-coated slide and a 24x24-cm coverslip was gently laid on top. Once the worms extruded the embryos, slides were placed on a metal block on dry ice for >10 min. The coverslip was then flicked off with a scalpel blade, and the samples were fixed in methanol at −20°C for 30 min (except for GFP, where the methanol treatment lasted for 5 min). Embryos were stained using standard procedures. Secondary antibodies were anti–sheep, anti-mouse, or anti-rabbit conjugated to Alexa Fluor 488, Alexa Fluor 594, and Alexa Fluor 647 (1:1,000, Thermo Scientific). DNA was visualized with Hoechst 33258 (Thermo Scientific, 1.5 μg/ml final concentration in PBS, 0.05% Tween-20) or DAPI (Sigma Aldrich, 1 μg/ml final concentration in PBS, 0.05% Tween-20). Embryos were mounted in ProLong Diamond antifade mountant (Thermo Scientific).

Plasmids. *C. elegans smo-1*, *ubc-9, gei-17, and air-2* cDNAs were cloned in the pHISTEV30a vector that includes an N-terminal hexahistidine tag followed by a TEV protease recognition site (Pelisch and Hay, 2016; Pelisch et al., 2014). Full length KLP-19 and fragments were codon-optimised for expression in *E. Coli* (Genscript) and sub-cloned in the pLou3 vector containing a 6xHis-MBP tag, followed by a TEV protease site. BUB-1(1-689) was codon-optimised for expression in *E. Coli* (Genscript) and sub-cloned in the pHISTEV30a vector containing a 6xHis tag, followed by a TEV protease site. GEI-17(423-602) and BUB-1(2-551) were cloned into pLou3 by Gibson Assembly (NEBuilder HiFi, NEB). All constructs were verified by sequencing.

SMO-1, UBC-9, and GEI-17 purification. SMO-1, UBC-9, and GEI-17 were tagged in their N-terminus with a 6xHis tag followed by a TEV protease cleavage site and expressed and purified essentially as described (Pelisch and Hay, 2016; Pelisch et al., 2014). The basic protocol consisted in an initial purification over a Ni-NTA column (QIAGEN), followed by 6xHis-TEV protease treatment. Any uncleaved material, as well as the 6xHis tag, and the 6xHis-TEV protease were removed with Ni-NTA beads, and the resulting untagged proteins were further purified by size exclusion chromatography using a HiLoad 16/600 Superdex 75 pg column (for SMO-1 and UBC-9, GE Healthcare) or a HiLoad 16/600 Superdex 200 pg column (for GEI-17, GE Healthcare). Lysis was performed in 50 mM Tris, 500 mM NaCl, 10 mM imidazole, 0.5 mM TCEP, pH 7.5 (with protease inhibitors, Roche). Subsequent purification steps were performed with 50 mM Tris, 150 mM NaCl, 0.5 mM TCEP, pH 7.5, and varying imidazole concentrations.

KLP-19 purification. KLP-19 and fragments were expressed as N-terminal fusions to 6xHis-MBP, followed by a TEV protease cleavage site. Bacterial cultures were grown until OD_{600} = 0.8, cooled on ice, and induced with 0.1 mM IPTG for 16 hs at 20°C. The bacterial cells were harvested by centrifugation (5,000 g for 20 min at 4ºC) and the cell pellet was resuspended in 50 ml of lysis buffer per L of culture [50 mM Tris, 300 mM KCl, 2 mM $MgSO_4$, 0.5 mM EGTA, 0.5 mM TCEP, and Complete protease inhibitor cocktail tablet, EDTA-free (Roche), pH 7.5]. Bacteria were lysed by sonication (Digital Sonifier, Branson) for 8 X 20" pulses at 50% amplitude, with a 30-second cooling period between pulses. Samples were centrifuged (27,200 g for 45 min at 4ºC) to remove any insoluble material and the supernatant was loaded onto an amylose column (NEB) pre-equilibrated with lysis buffer. The column was washed successively with lysis buffer (~10 column volumes) and the fusion protein was then eluted with lysis buffer supplemented with 10 mM maltose. TEV protease was added (1 mg of TEV protease per 30 mg of the fusion protein) and incubated ~16 hours at 4°C. Samples were centrifuged (3,900 g for 15 min at 4°C) to remove any precipitated material and buffer was exchanged to 80 mM PIPES-KOH, 50 mM KCl, 2 mM $MgSO_4$, 0.5 mM EGTA, 0.5 mM TCEP, pH 6.9 using Zeba spin columns (Thermo Scientific). For full-length KLP-19, the protein was concentrated and purified over a Superose 6 prep grade XK 16/70 column (GE Healthcare). For the 651-1083 fragment, a Ni-NTA column was used to remove the 6xHis-TEV protease, the 6xHis-MBP tag, and any remaining uncleaved 6xHis-MBP-tagged KLP. KLP-19(651-1083) was further purified by size exclusion chromatography with a HiLoad 16/600 Superdex 200 pg column. Purified proteins were aliquoted, flash-frozen in liquid nitrogen, and stored at -80°C.

Pull-down experiments. For the pulls-down in Figures 7H and 7H, MBP and MBP fusion proteins were expressed in bacteria, bound to amylose beads and washed extensively before pull-down experiments. SUMO-modified GEI-17(133-509) (~2 μM) or KLP-19(651-1083) (~5 μM) were incubated for ~5 minutes on ice with MBP or MBP-fusion proteins as indicated (5 μM) and 30 μl of amylose beads in total volume of 70 μl. Samples were buffered in 50 mM Tris, 150 mM NaCl, 0.5 mM TCEP, 5% (v/v) glycerol, pH 7.5 (binding buffer). Subsequently, amylose beads were collected on

the bottom of the tube by centrifugation and the supernatant was aspired. Beads were resuspended in 0.5 ml of binding buffer and overlaid on 1.4 ml of 50 mM Tris, 150 mM NaCl, 0.5 mM TCEP, 5% (v/v) glycerol, 10% (w/v) sucrose, pH 7.5 in a new tube. Beads were then collected by centrifugation, the supernatant was aspired and the washing step was repeated. All washing procedures were carried out as quickly as possible using pre-chilled buffers. Bound material was eluted from the beads by addition of SDS-PAGE loading buffer and analyzed by Western blotting.

In vitro sumoylation. Conjugation assays contained 50 mM Tris-HCl, 5 mM dithiothreitol, 5 mM $MgCl_2$, 2 mM ATP, 20 μM SUMO, 100 nM of SAE1/SAE2, 140 nM UBC-9 (for GEI-17-dependent reactions), and 3.3 μM UBC-9 (for GEI-17-independent reactions). KLP-19 was used at 1 μM, KLP-19(651-1083) at 6 μM and GEI-17 ranged from 12.5 to 50 nM, as indicated. Reactions were incubated at 37°C for the indicated times. Reactions were analysed by coomassie staining, regular western blotting, or dual-color western blotting using an Odyssey CLx (LI-COR).

Duolink® *in situ* Proximity Ligation Assay (PLA®). Proximity ligation assays were performed using primary antibodies directly coupled to the PLA probes or using secondary antibody PLA probes (Sigma-Aldrich). For the direct PLA, ~35 worms were placed on a drop of 4 μl of M9 worm buffer in a poly-D-lysine-coated slide and a coverslip was gently laid on top. Once the worms extruded the embryos, slides were freeze-cracked: placed on a metal block on dry ice for >10 min., the coverslip taken off with a scalpel blade, and samples fixed in methanol at −20°C for 30 min. After sequential washes (5 min each) with PBS + 0.5% Triton X-100, PBS + 0.1% Tween-20, and PBS, slides were incubated with the monoclonal α-SMO-1 (6F2/D1, 10 μg/ml) and α-AIR-2, α-BUB-1, or α-KLP-19 (all at 10 μg/ml), previously coupled to the PLA oligonucleotide arms using the Duolink® in situ Probemaker overnight at 4°C. Ligation and amplification were performed as detailed by the manufacturer. Controls omitting either of the antibodies gave no PLA signal. For indirect PLA, the same primary antibodies were used (unlabeled) and after an overnight incubation at 4°C, slides were incubated with anti-mouse and anti-rabbit secondary antibodies coupled to the PLA oligonucleotide probes. Ligation and amplification were performed as detailed by the manufacturer. In both cases, slides were incubated in Hoechst 33258 at 1.5 μg/ml in PBS + 0.1% Tween-20 for 5 min. Slides were mounted in 4% n-propyl-gallate, 90% glycerol, in PBS and were imaged using a DeltaVision Elite microscope.

In vivo identification of SUMO conjugation sites. Worms expressing His_6-SUMO(L88K) were grown in liquid culture with S-medium (50 mM potassium phosphate, 100 mM NaCl, 10 mM potassium citrate, 3 mM $CaCl_2$, 3 mM $MgCl_2$, 5 µg/ml cholesterol, and trace metals, pH 6) supplemented with OP50 bacteria. Five different sets of synchronised liquid cultures were lysed 24 h after >75% of the population was at the L4 stage. Twenty five grams of worm pellet were lysed with 125 ml of lysis buffer (6 M Gu-HCl, 100 mM Na_2HPO_4/NaH_2PO_4, 10 mM Tris-HCl, 10 mM imidazole, 5 mM β-mercaptoethanol, pH 8). The sample was sonicated with 6 cycles of 20 sec on and 20 sec off in ice using a tip sonicator at 55% amplitude. After centrifugation at 45,000 g for 30 min at 4°C, the supernatant was passed through a 0.2 µm filter and protein concentration was measured using the BCA assay, with BSA as a standard. Protein concentration was 10 mg/ml in a total volume of 150 ml. Subsequently, 4 ml of packed Ni-NTA beads pre-equilibrated in lysis buffer were added and incubated overnight at 4°C in 4 50-ml tubes. At this stage, 100 µl of the mix were taken, beads were pelleted, and the bound and flow-through fractions were run on SDS-PAGE to perform anti-SUMO western blotting. For purification of the His_6-sumoylated proteins, the content of the 4 tubes were loaded on a column, and the Ni-NTA beads were washed with 5 column volumes (CVs) of lysis buffer, 10 CVs of Urea Buffer I (8 M Urea, 100 mM Na_2HPO_4/NaH_2PO_4, 10 mM Tris-HCl, 10 mM imidazole, 5 mM β-mercaptoethanol, pH 8), 10 CVs of Urea Buffer II (8 M Urea, 100 mM Na_2HPO_4/NaH_2PO_4, 10 mM Tris-HCl, 10 mM imidazole, 5 mM β-mercaptoethanol, pH 6.3), 5 CVs of Urea Buffer I, and eluted with 3 CVs of elution buffer (8 M Urea, 100 mM Na_2HPO_4/NaH_2PO_4, 10 mM Tris-HCl, 200 mM imidazole, 5 mM β-mercaptoethanol, pH 8). We obtained approximately 4 mg of protein, as judged by quantitation using the BCA method.

Proteins were concentrated on Vivacon 2 30-kDa-filter units at 3,000 g (20°C) until the filters were dry. The filter was washed twice with 3 ml of UA Buffer (8 M urea, 100 mM Tris-HCl pH 8.0). Then, 1.5 ml of 50 mM chloroacetamide (CAA) in UA buffer were added and incubated for 20 min in the dark. The filter was centrifuged at 3,000 g until dry, washed with 3 ml of UA buffer, and then twice with 3 ml of IP buffer (50 mM MOPS pH 7.2, 10 mM Na_2HPO_4, 50 mM NaCl). Lys-C was added at an enzyme to protein ratio of 1:50 in a final volume of 10 CVs IP buffer. Incubation proceeded at 37°C (in a wet chamber) for ~16 h. Filters were then centrifuged at 3,000 g until dry and washed twice with 270 µl of IP buffer. Peptide samples were incubated at 95 °C for 5 min to inactivate residual lys-C activity. Peptides were stored at -80°C for further processing. Then, Glu-C was added to the filters at an enzyme to protein ratio of 1:100 in IP buffer. Filter units were incubated at room temperature (in a wet chamber) for ~16 h and centrifuged at 3000 g until the filter was dry. Filters were washed twice with 270 µl of IP buffer and the peptides were then incubated at 95 °C for 5 min to inactivate residual Glu-C activity. Peptides were stored at -80°C.

For IP, 19 µg of BS^3 crosslinked anti-K-ε-GG antibody were added and incubated ON (~24 h) at 4 °C while rotating. Samples were centrifuged at 1,000 g for 1 min at 4°C and beads were allowed to settle for 5 min on ice. Supernatant was transfered to another tube, leaving approximately 100 µl of supernatant fraction on top of the beads. Beads were resuspended and transferred into an 0.5 ml eppendorf tube. The bead-supernatant slurry was centrifuged at 1,000 g for 1

min at 4°C and beads were allowed to settle for 5 min on ice. The rest of the supernatant fraction was transferred to the same flow-through tube used in the last step of the protocol. Flow-through fraction was stored at -80 °C. Beads were washed twice by adding 150 µl of cold IP buffer, centrifugation at 1,000 g for 1 min 4 °C and allowing the beads settle for 5 min on ice. Solutions were stored at -80 °C. K-ε-GG peptides were eluted twice with 50 µl of 0.15% TFA. Peptide solution was separated from beads and beads were stored in 3 µl of IP buffer at 4 °C (50% bead solution). Peptides were desalted on 3x C18 stagetips and eluted with K-ε-GG buffer B (80% acetonitrile, 0.1% TFA). Peptides were concentrated in a speed-vac and resuspended in 0.1% TFA.

Liquid chromatography tandem mass spectrometry (LC-MS/MS). Each desalted sample of peptides was analysed twice using EASY-nLC 1000 nano-flow UHPLC system, EASY-Spray ion source and Q Exactive hybrid quadrupole-Orbitrap mass spectrometer (all Thermo Scientific). Peptides were loaded onto 2 cm Acclaim PepMap 100 C18 nanoViper pre-column (75 µm inner diameter; 3 µm particles; 100 Å pore size) at a constant pressure of 800 bar and separated using 50 cm EASY-Spray PepMap RSLC C18 analytical column (75 µm inner diameter; 2 µm particles; 100 Å pore size) maintained at 45°C. Exploratory analysis was performed with 10% of the sample and peptides were separated during either a 35 or 60 min linear gradient of 5−25% or 5-22% (vol/vol) of acetonitrile in 0.1% (vol/vol) of formic acid, respectively, at a flow rate of 250 nL/min, followed by a 10 or 12 min linear increase of acetonitrile to 50 or 40% (vol/vol), respectively. Total length of the gradient including column washout and re-equilibration was 60 or 90 min, respectively. Comprehensive peptide analysis was performed using a 60 min linear gradient of 5−22% acetonitrile in 0.1% formic acid at a flow rate of 250 nL/min, with a subsequent 12 min linear increase of acetonitrile to 40%. The overall length of the gradient during comprehensive analysis was 90 min.

Peptides eluting from the LC column were charged using electrospray ionization and MS data was acquired online in a profile spectrum data format. Full MS scan covered a mass range of mass-to-charge ratio (m/z) 300−1800 or 300−1600 during exploratory or comprehensive peptide analysis, respectively. Target value was set to 1 000 000 ions with a maximum injection time (IT) of 20 ms and full MS was acquired at a mass resolution of 70 000 at m/z 200. Data dependent MS/MS scan was initiated if the intensity of a mass peak reached a minimum of 20 000 ions. During exploratory LC-MS/MS analysis, up to 10 most abundant ions were selected using 2 Th mass isolation range when centered at the parent ion of interest. For comprehensive analyses, the most abundant ion was exclusively picked for MS/MS. Selection of molecules with peptide-like isotopic distribution was preferred. Target value for MS/MS scan was set to 500 000 with a maximum IT of 60 ms and resolution of 17 500 at m/z 200 for exploratory, or maximum IT of 1000 ms and a resolution 35 000 at m/z 200 for comprehensive peptide analyses. Precursor ions were fragmented by higher energy collisional dissociation (HCD) using a normalised collision energy of 30 and fixed first mass was set to m/z 100. Precursor ions with undetermined, single, or high (>8) charge state were rejected. Ions triggering a data-dependent MS/MS scan were placed on the dynamic exclusion list for 40 s (exploratory analyses) or 60 s (comprehensive analyses) and isotope exclusion was enabled.

Analysis of MS data. Raw MS files were analysed using MaxQuant software package (version 1.3.0.5) (Cox and Mann, 2008) and peak lists were searched with an integrated Andromeda search engine (Cox et al., 2011) against an entire *C. elegans* UniProtKB proteome (Apweiler et al., 2004) including canonical and isoform sequences downloaded in a FASTA format in May 2014 or May 2016 and supplemented with the sequence of *C. elegans* SUMO(L88K) and the following *H. sapiens* proteins: UBC9 (P63279), SAE1 (Q9UBE0) and SAE2 (Q9UBT2). Raw files were divided into two parameter groups based on the specificity of proteolysis applied during sample preparation. Hydrolysis of peptide bonds C-terminal to Lys residues with a maximum of three missed cleavages was allowed for peptides processed exclusively with Lys-C. Samples acquired after an additional Glu-C digestion were analysed with enzyme specificity set to C-terminal to Lys, Glu or Asp with a maximum of five missed cleavages. Carbamidomethylation of cysteine residues was specified as a fixed modification and oxidation of methionines, acetylation of protein N-termini, and Gly-Gly adduct on internal lysine residues were selected as variable modifications. Maximum peptide mass of 10 000 Da was allowed, multiplicity was set to 1 and re-quantify option was disabled. Decoy sequence database was generated using Lys as a special amino acid. Default values were chosen for the rest of the parameters.

The mass spectrometry proteomics data have been deposited to the ProteomeXchange Consortium via the PRIDE (Vizcaino et al., 2016) partner repository with the dataset identifier PXD005202.

Statistical analysis. Bivalent-to-pole distances in monopolar spindles (Figure 1H) and non-RC KLP-19 localisation (Figure 3K) were analysed with the Kruskal-Wallis test, followed by Dunn's post-test. In Figures 3H and S5C, fluorescence intensity (corrected for background fluorescence) were measured in ImageJ and then analysed using Mann-Whitney tests. All tests were performed with GraphPad Prism version 6.00 for Mac OS X (GraphPad Software, La Jolla California USA).

Supplemental References

Apweiler, R., Bairoch, A., Wu, C.H., Barker, W.C., Boeckmann, B., Ferro, S., Gasteiger, E., Huang, H., Lopez, R., Magrane, M., *et al.* (2004). UniProt: the Universal Protein knowledgebase. Nucleic Acids Res 32, D115-119.

Bembenek, J.N., Richie, C.T., Squirrell, J.M., Campbell, J.M., Eliceiri, K.W., Poteryaev, D., Spang, A., Golden, A., and White, J.G. (2007). Cortical granule exocytosis in C. elegans is regulated by cell cycle components including separase. Development 134, 3837-3848.

Brenner, S. (1974). The genetics of Caenorhabditis elegans. Genetics 77, 71-94.

Bruderer, R., Tatham, M.H., Plechanovova, A., Matic, I., Garg, A.K., and Hay, R.T. (2011). Purification and identification of endogenous polySUMO conjugates. EMBO Rep 12, 142-148.

Burrows, A.E., Sceurman, B.K., Kosinski, M.E., Richie, C.T., Sadler, P.L., Schumacher, J.M., and Golden, A. (2006). The C. elegans Myt1 ortholog is required for the proper timing of oocyte maturation. Development 133, 697-709.

Cheeseman, I.M., and Desai, A. (2005). A combined approach for the localization and tandem affinity purification of protein complexes from metazoans. Science's STKE : signal transduction knowledge environment 2005, pl1.

Cox, J., and Mann, M. (2008). MaxQuant enables high peptide identification rates, individualized p.p.b.-range mass accuracies and proteome-wide protein quantification. Nat Biotechnol 26, 1367-1372.

Cox, J., Neuhauser, N., Michalski, A., Scheltema, R.A., Olsen, J.V., and Mann, M. (2011). Andromeda: a peptide search engine integrated into the MaxQuant environment. J Proteome Res 10, 1794-1805.

Dickinson, D.J., Pani, A.M., Heppert, J.K., Higgins, C.D., and Goldstein, B. (2015). Streamlined Genome Engineering with a Self-Excising Drug Selection Cassette. Genetics 200, 1035-1049.

Kamath, R.S., and Ahringer, J. (2003). Genome-wide RNAi screening in Caenorhabditis elegans. Methods 30, 313-321.

Morawska, M., and Ulrich, H.D. (2013). An expanded tool kit for the auxin-inducible degron system in budding yeast. Yeast 30, 341-351.

Oegema, K., Desai, A., Rybina, S., Kirkham, M., and Hyman, A.A. (2001). Functional analysis of kinetochore assembly in Caenorhabditis elegans. J Cell Biol 153, 1209-1226.

Pelisch, F., and Hay, R.T. (2016). Tools to Study SUMO Conjugation in Caenorhabditis elegans. In SUMO: Methods and Protocols, S.M. Rodriguez, ed. (New York, NY: Springer New York), pp. 233-256.

Pelisch, F., Sonneville, R., Pourkarimi, E., Agostinho, A., Blow, J.J., Gartner, A., and Hay, R.T. (2014). Dynamic SUMO modification regulates mitotic chromosome assembly and cell cycle progression in Caenorhabditis elegans. Nat Commun 5, 5485.

Powers, J., Rose, D.J., Saunders, A., Dunkelbarger, S., Strome, S., and Saxton, W.M. (2004). Loss of KLP-19 polar ejection force causes misorientation and missegregation of holocentric chromosomes. J Cell Biol 166, 991-1001.

Praitis, V., Casey, E., Collar, D., and Austin, J. (2001). Creation of low-copy integrated transgenic lines in Caenorhabditis elegans. Genetics 157, 1217-1226.

Schumacher, J.M., Golden, A., and Donovan, P.J. (1998). AIR-2: An Aurora/Ipl1-related protein kinase associated with chromosomes and midbody microtubules is required for polar body extrusion and cytokinesis in Caenorhabditis elegans embryos. J Cell Biol 143, 1635-1646.

Vizcaino, J.A., Csordas, A., Del-Toro, N., Dianes, J.A., Griss, J., Lavidas, I., Mayer, G., Perez-Riverol, Y., Reisinger, F., Ternent, T., *et al.* (2016). 2016 update of the PRIDE database and its related tools. Nucleic Acids Res.

Wallenfang, M.R., and Seydoux, G. (2000). Polarization of the anterior-posterior axis of C. elegans is a microtubule-directed process. Nature 408, 89-92.

Zhang, L., Ward, J.D., Cheng, Z., and Dernburg, A.F. (2015). The auxin-inducible degradation (AID) system enables versatile conditional protein depletion in C. elegans. Development 142, 4374-4384.
